# Nested whole-genome duplications coincide with diversification and high morphological disparity in Brassicaceae

**DOI:** 10.1038/s41467-020-17605-7

**Published:** 2020-07-30

**Authors:** Nora Walden, Dmitry A. German, Eva M. Wolf, Markus Kiefer, Philippe Rigault, Xiao-Chen Huang, Christiane Kiefer, Roswitha Schmickl, Andreas Franzke, Barbara Neuffer, Klaus Mummenhoff, Marcus A. Koch

**Affiliations:** 1 Centre for Organismal Studies, University of Heidelberg, Im Neuenheimer Feld 345, 69120 Heidelberg, Germany; 2 GYDLE, 1135 Grande Allée Ouest, Québec, QC G1S 1E7 Canada; 3 Department of Botany, Faculty of Science, Charles University, Benátská 2, 128 01, Prague, Czech Republic; 4 Department of Biology, Systematic Botany, University of Osnabrück, Barbarastraße 11, 49076 Osnabrück, Germany; 5 Present Address: South-Siberian Botanical Garden, Altai State University, Lenina Ave. 61, 656049 Barnaul, Russia; 6 Present Address: School of Life Sciences, Nanchang University, 330031 Nanchang, China

**Keywords:** Phylogenetics, Taxonomy, Phylogenomics, Plant evolution

## Abstract

Angiosperms have become the dominant terrestrial plant group by diversifying for ~145 million years into a broad range of environments. During the course of evolution, numerous morphological innovations arose, often preceded by whole genome duplications (WGD). The mustard family (Brassicaceae), a successful angiosperm clade with ~4000 species, has been diversifying into many evolutionary lineages for more than 30 million years. Here we develop a species inventory, analyze morphological variation, and present a maternal, plastome-based genus-level phylogeny. We show that increased morphological disparity, despite an apparent absence of clade-specific morphological innovations, is found in tribes with WGDs or diversification rate shifts. Both are important processes in Brassicaceae, resulting in an overall high net diversification rate. Character states show frequent and independent gain and loss, and form varying combinations. Therefore, Brassicaceae pave the way to concepts of phylogenetic genome-wide association studies to analyze the evolution of morphological form and function.

## Abstract (web summary)

As one of the most successful angiosperm clades with ~4000 species, the mustard family has been diversifying into many evolutionary lineages. Here, the authors construct plastid-based phylogeny and show nested whole-genome duplications coincide with diversification and high morphological disparity.

## Introduction

The diversity of morphological traits and characters is dazzling, and its non-uniform realization during the evolution of plant life^1^ allowed land plants to diversify into almost all terrestrial habitats. Morphological diversity is considered the major product of macroevolution^1,2^. It is often expressed as disparity, which by its simplest definition—that we will follow here—is the amount of morphological variation present in a given taxon relative to the total morphological variation in the set of taxa under investigation^2^. Morphological disparity and taxonomic richness are not independent, since morphological differences are the basis for taxonomic descriptions, which are then translated into different species and genera. Nevertheless, the two factors are not necessarily correlated, as high disparity can be maintained in clades of low taxonomic richness, and plant diversification can occur without expanding morphological variation (or the morphospace) within taxa^2,3^. In animal clades, early high disparity with subsequent decrease is the predominant pattern in deep evolutionary histories^4^, whereas it is intriguing to notice that for the past ~100 my (million years) large clades of land plants, such as conifers, ferns, and angiosperms, have maintained high levels of disparity^2^. However, in most cases the intrinsic or extrinsic factors driving disparity are unknown^2,4^.

Whole-genome duplications (WGD) are among the factors that have the potential to increase disparity^5^, and they have played a fundamental role during land plant evolution^6^. WGD arises either via auto- or allopolyploidization, and the mode of polyploidization is often unknown for paleopolyploids; herein we cannot distinguish between the two. WGDs may not only provide the opportunity to develop new character states, key characters, or even evolutionary innovations, but also generally increase the potential to implement increased character and trait variation in a clade and thereby its disparity. Subsequently, increased morphological disparity may provide the basis to further diversify and eventually even radiate in a changing spatiotemporal context, while only a minority of characters and their states contribute to key traits and innovations and thereby drive clade diversification and radiation.

Polyploidy is common among land plants, with up to 24% of species being recent polyploids^7^, and all flowering plants have at least one if not numerous ancient polyploidy events in their history^8^. While the evolutionary potential of polyploids has been debated in recent years^9^, the importance of this phenomenon for angiosperm diversification has been corroborated by numerous studies. All angiosperms share at least two paleopolyploidization events^10^, and since the first identification of such major events, the so-called At-α, At-β, and At-γ events, in the genome of the model plant *Arabidopsis thaliana*^11^, a multitude of WGDs have been identified in many angiosperm lineages^12^. Many have been dated to times of major environmental change and transitions between geological epochs, like the Cretaceous-Paleogene boundary^13^ or Pleistocene glaciation-deglaciation cycles^14^. An adaptive advantage of polyploids is thought to be caused by the availability of duplicate genes for sub- and neofunctionalization and thus the emergence of new traits^15^. Clades having undergone WGDs often also have higher diversification rates^16^, although the timings of WGD and diversification do not necessarily coincide. Rather, a diversification leading to a species-rich clade occurs sometime after the split from another lineage, which remains relatively species poor. The so-called WGD radiation lag-time model^17^ attempts to explain this observation that can be detected both among angiosperms^17^ and in the Brassicaceae family^17,18^. Since polyploidization per se is causing major disadvantages, e.g., during mitosis and meiosis, lineages subjected to WGD usually experience a subsequent phase of diploidization^19^ often accompanied by drastic genome reorganization and genome size reduction^20^. These processes might be key to subsequent evolutionary success of respective clades evolving new traits and innovations. Some examples for key traits presumably originating from WGDs exist, such as synthesis of glucosinolates, secondary compounds involved in insect defense mechanisms in the order Brassicales, following At-β^21^ or evolution of the pentamerous flower in Pentapetalae triggering plant-pollinator coevolution following At-γ^22^. However, there are few studies systematically linking morphological diversity and its evolution with WGDs^5^.

The Brassicaceae are among the 15 largest angiosperm families, comprising almost 4000 species in 351 genera^23^, including the model species *Arabidopsis thaliana*, *Arabidopsis lyrata* and *Arabis alpina*, as well as important crops, such as cabbage and rapeseed. A taxonomic system of 51 monophyletic tribes has been proposed to subdivide the family, and they can be organized into several major evolutionary lineages: The tribe Aethionemeae diverged from the rest of the family around 32 million years ago^24^ (mya). The other 50 tribes are grouped into three^25^, four^26^, or five lineages^27,28^, which started diversifying ~23 mya^24^. The phylogenetic positions of these tribes and lineages are still unresolved, in part due to conflicting signals from nuclear and plastid data. Furthermore, potential undersampling of the ingroup may have contributed to difficulties in generating a fully resolved phylogeny-two recent studies included 55 species from 45 genera in 29 tribes^27^, and 79 species from 72 genera in 50 tribes^28^, often only sampling one representative per tribe and few species from other families of the order Capparales. Selection of genes for phylogenetic reconstructions following different strategies, unresolved reticulate evolutionary scenarios, and taxonomic inaccuracy, such as erroneous circumscription of tribe Stevenieae^28^, may also have contributed to these inconsistencies.

The evolutionary history of Brassicaceae has been shaped by repeated cycles of WGDs, most notably At-α preceding the divergence of the family and the earlier At-β event within the Brassicales^11,21^. In addition to these paleopolyploidizations, so-called mesopolyploidization events^29^ have been discovered and confirmed in eleven out of the 51 Brassicaceae tribes^30,31^, among them the agronomically important tribe Brassiceae, and explicitly shown to be absent from various other tribes^31,32^ (Supplementary Table 1). Post-mesopolyploidization genomes are characterized by extensive diploidization through chromosomal rearrangements, genome size reduction and fractionation; however, duplicated genomic regions are still detectable^29,33^. Interestingly, the occurrence of WGDs seems largely uncoupled from shifts in diversification rate that have been observed in nine tribes^34^. Neopolyploidy is common in Brassicaceae as well, with polyploidy detected in 43% of species, and the percentage of neopolyploids per tribe is weakly correlated with species richness^24^, indicating polyploidization is a continuously running process over evolutionary time scales in Brassicaceae.

Brassicaceae are known for parallel evolution of most morphological characters in most lineages. Therefore, character state reconstruction analyses have often failed to provide any evolutionary signature on the tribal or higher lineage level^27^. This led to the idea that morphological characters in Brassicaceae vary strongly and are of little use for phylogenetic reconstruction. However, this has never been analyzed rigorously on the familial level using the entire morphospace needed to determine, identify, and distinguish genera taxonomically. Several studies have revealed extensive homoplasy and convergent evolution for nearly all morphological characters, such as fruit shape^27^, flower characters^35^, shape of leaf base^27^, and leaf complexity^31,36^, and even for complex traits such as life history^18^. There is no obvious morphological key character or innovation that evolved after mesopolyploidization and triggered subsequent diversification. This raises the question if and how WGDs act on disparity and diversification in a successful and constantly diversifying angiosperm family like Brassicaceae^34^.

Here, we present a plastome phylogeny combined with a comprehensive morphological dataset of the entire Brassicaceae as well as a complete species checklist comprising all currently described 3977 species and 351 genera. This allows us to establish a phylogenetically and temporally resolved maternal evolutionary framework in which the herein presented, as well as future results, can be interpreted. We analyze if mean morphological disparity has increased after tribal and lineage specific WGDs, and we study whether early diverging disparity among main evolutionary lineages coincides with shifts of net diversification rates in the early evolutionary history of the family. We propose that WGDs contribute to morphological disparity, but do not necessarily trigger immediate clade diversification and radiation. Our study thus highlights the evolution of morphological diversity as a so far underexplored aspect of the general importance of WGDs for the evolutionary success of a lineage over more than 30 million years.

## Results

### The taxonomic and phylogenetic backbone concept

**Fig. 1:**
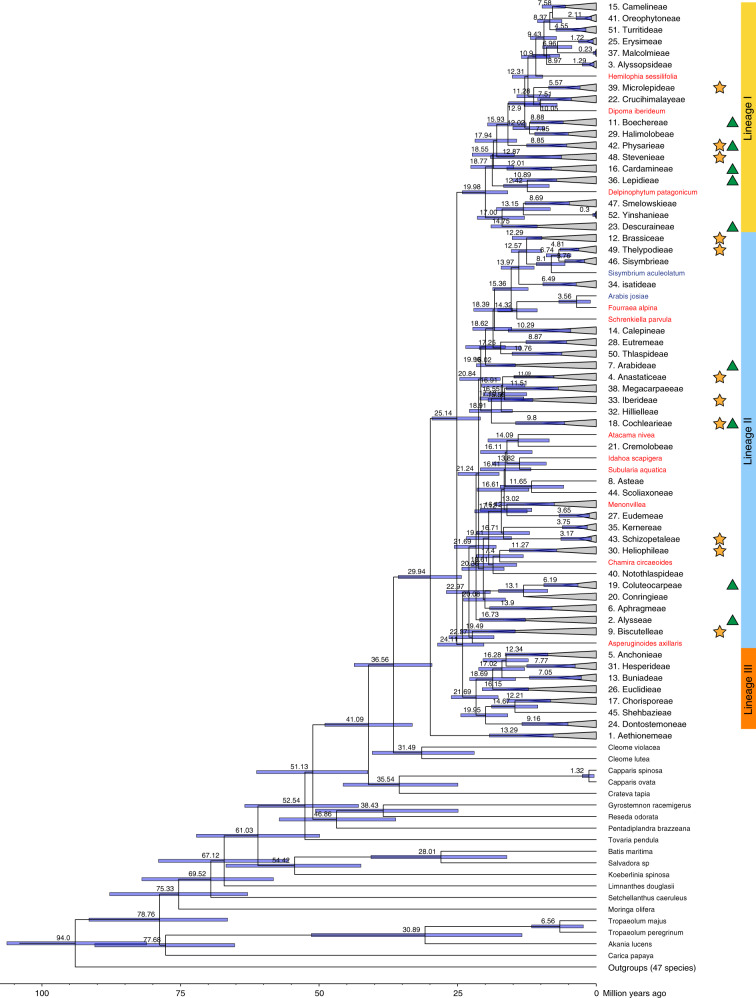
Brassicaceae maternal timeline. A simplified version of the BEAST analysis of coding regions from 60 plastid genes (Supplementary Fig. 2) is shown. Two hundred and fifty Brassicaceae species as well as representatives of 15 additional Brassicales families and 47 species (indicated here as outgroups) from other Rosidae families were included. Within Brassicaceae, tribes included with multiple species are represented by gray triangles and the respective crown ages are given. Taxa indicated in red refer to the three categories ‘remote genera’, ‘newly assigned genera’ and ‘unclear and remaining unassigned’; taxa indicated in blue are in need of a new genus name, because they create a polyphyly problem (Supplementary Note 2). Divergence time estimates are based on combined 28 BEAST analyses, resulting in 21,038 analyzed states. Ninety-five percent HPD intervals are represented by blue node bars. Assignment to the three lineages are given, and mesopolyploidization events^30,31^ as well as significant rate shifts^34^ are indicated with yellow stars and green triangles, respectively. Source data are provided as a Source Data file.

Past phylogenetic reconstructions of Brassicaceae have revealed incongruences between biparentally inherited nuclear and predominantly maternally inherited plastome marker based phylogenies, most likely due to rapid diversification, intertribal hybridization^37^ and nuclear introgression^38^. A recently published nuclear phylogeny provided substantial progress and comprises many of the tribes described in Brassicaceae^30^. However, there is still no comprehensive phylogeny available on the tribal level that also considers all deep nodes within tribes. Furthermore, the lack of consistently estimated divergence times over the entire family restricts its potential use for phylogenetic comparative methods. Here, we rely on a plastome phylogeny, allowing us to estimate divergence times, extract age estimates, and conduct phylogenetically corrected downstream analyses. Furthermore, the use of the plastome, assessed via a genome skimming approach, enabled us to include samples from rare herbarium specimens. Our family-wide taxon sampling for the phylogenetic analysis covers all tribes of the Brassicaceae and aims at representing the deepest known phylogenetic split within each tribe. The presented maternal phylogeny includes 153 out of the 351 genera currently recognized in the Brassicaceae. The tree topology based on coding gene space of the plastome (Supplementary Fig. 1) is fully congruent with earlier plastome analyses encompassing a fraction of taxa (5–24% of Brassicaceae genera) compared to the taxon set included herein^24,28,39^. Furthermore, our maternal phylogeny (Supplementary Fig. 1) is highly consistent with published phylogenetic-systematic studies in respect to taxonomic conclusions on the tribal and genus level (Supplementary Note 1). Two recent tribal level phylogenies of Brassicaceae based on genome-wide nuclear sequence data^27,28^ are somewhat inconsistent with each other and also with our plastome phylogeny, mostly in respect to the deeper splits within the family. Consequently, we did not analyze our data using phylogenetic information and tree structure on genus level, but restricted conclusions on the level of higher order evolutionary lineages and comparing tribes directly with each other. Our phylogenetic analysis resulted in a well-supported maternal topology, which is summarized on the tribal level in Fig. 1 (detailed tree in Supplementary Fig. 1/2; a phylogenetic tree based on the nuclear encoded rDNA cistron, in contrast, is largely unresolved and shown in Supplementary Fig. 3, details are given in Supplementary Note 1). Topologies from maximum likelihood (ML) reconstruction and divergence time estimations are congruent, and robustness is demonstrated by high bootstrap support for almost all nodes in our RAxML analysis (Supplementary Fig. 1). As in previous studies, Brassicaceae can be grouped into an early branching lineage represented only by tribe Aethionemeae (Fig. 1), with a split time of ~30 my (Brassicaceae crown group age), followed by diversification of the major evolutionary lineages I, II, and III (following lineage assignment by Koch & Al-Shehbaz^25^) 24–25 mya. This is consistent with time estimates based on data from both plastid and nuclear genomes^24,34,39,40^. Additional subclades of evolutionary lineages were introduced based on nuclear genomic data^28^, and we also analyzed our data using these phylogenetic concepts since we cannot resolve the topological conflict among phylogenetic trees.

Previously detected significant shifts in diversification rates^34^ and mesopolyploidization events^30,31^ are restricted to lineages I and II and are indicated as green triangles and yellow stars, respectively, in Fig. 1. Our sampling strategy allowed us to extract stem group ages for all 51 tribes and crown group ages of 24 tribes directly from the dated phylogeny. Correlation of these crown group ages with those obtained recently from tribal analyses based on nuclear encoded rDNA^34^ was very high and significant (Spearman’s rank correlation coefficient 0.89, *P*-value < 0.001, see Supplementary Fig. 4). Thus, we used time estimates from that study to fill our missing data points and calculate the lag-phase between clade divergence and diversification for all tribes (Supplementary Table 2).

We compiled a comprehensive Brassicaceae species checklist including every accepted genus and species. This checklist comprises 3977 species from 351 genera, most of which could be assigned to one of the 51 tribes, with only eleven orphan genera, for which a reliable phylogenetic placement on the familial level was provided (Fig. 1, Supplementary Fig. 1/2). The detailed descriptions and taxonomic discussions are provided in Supplementary Note 2, and a species checklist providing accepted names is also accessible via BrassiBase (version 1.3; https://brassibase.cos.uni-heidelberg.de/). Our species and genus checklist, which provides accepted names for species and subspecies, incorporates changes to 860 previously accepted species names, including changes for more than 3500 synonyms (often wrongly assigned in the past), and resulted in a list with 15,365 updated data entries.

### Morphological characters are homoplastic in Brassicaceae

**Fig. 2:**
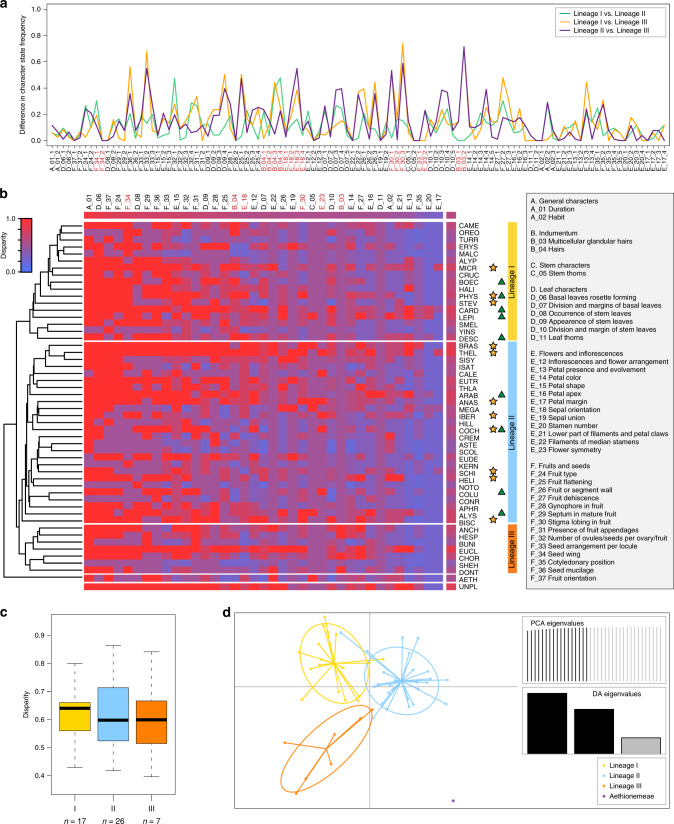
Morphological characters and their disparity across tribes and lineages. **a** Pairwise differences in character state frequency for all 111 character states. **b** Heatmap of tribal disparity calculated directly from tribal level morphomatrix. Tribes are sorted by phylogeny (following Fig. 1), and characters are sorted by disparity, with the highest mean disparity values on the left. Assignment to the three lineages are given, and mesopolyploidization events^30,31^ as well as significant rate shifts^34^ are indicated with yellow stars and green triangles, respectively. **c** Boxplot of tribal mean disparity (direct) across the three lineages. No significant differences were detected between lineages. In the boxplot, center line represents median; upper and lower quartiles are indicated by box limits; whiskers represent 1.5× interquartile range and points are outliers. **d** DAPC analysis of tribal level disparity with a priori grouping of lineages. The 37 characters in six categories (A–F) are named in the grey box on the right. Characters with significant phylogenetic signal (Moran’s I) are highlighted in red below the pairwise differences and above the heatmap (see also Supplementary Fig. 7). Source data are provided as a Source Data file.

Evaluation of morphological variation on the genus level and across the entire Brassicaceae family (Supplementary Note 3) resulted in an interactive key enabling researchers to determine all genera, available at BrassiBase (https://brassibase.cos.uni-heidelberg.de/?action=key). The underlying matrix of the key (38 characters, 166 character states) was optimized for statistical analyses (37 characters, 111 character states); we also provide a matrix compiled on tribal level. For clarity, characters were arbitrarily grouped into six categories (A–F) referring to life form, hairs, stem, leaves, flowers and inflorescences, and fruits and seeds, respectively (Fig. 2, Supplementary Methods).

To visualize differences in presence or absence of character states between main evolutionary lineages, we assessed pairwise differences in character state frequency. High-pairwise differences (Fig. 2a, Supplementary Table 3) denote character states predominantly found in one lineage, such as multicellular glandular hairs (B03), which are restricted to four of the seven tribes of lineage III. Other character states with high differences between lineage III and the rest of the family are decurrent and connivent stigma lobing in fruits (F30) or seed wings (F34). Characters with low differences, such as ‘sepal orientation’ (E18) or ‘hairs’ (B04), may represent a set for which the evolutionary potential of character state diversification either evolved between the split of the major lineages and the onset of their diversification or were lost. However, only few character states showed pairwise differences higher than 0.5, and none reached a pairwise difference of 1 (Fig. 2a, Supplementary Table 3).

### Morphological disparity is not phylogenetically constrained

To assess the lineage- and tribe-specific potential for morphological diversity (despite the high degree of homoplasy) we analyzed morphological disparity. Rather than looking at individual characters or character states, disparity elaborates on the presence of possible character states within a defined taxonomical unit (in our case genera and tribes). Note that from here on, we will use the term disparity for the specific measure of disparity (observed character states/total character states). We did not detect significant differences in mean disparity among evolutionary lineages I, II, and III (Fig. 2c, Supplementary Fig. 5/6, Supplementary Tables 4/5), or using alternative lineage assignments^26,28^ (Supplementary Fig. 6, Supplementary Tables 4, 6, and 7). Furthermore, mean tribal disparity was randomly distributed across the phylogeny; we detected no phylogenetic signal (Moran’s I). This can be interpreted as an absence of phylogenetic constraints on phenotypic variation and the increasing number of realized character states. The character with the highest disparity was duration (life cycle), where both character states (annual and perennial) were observed in most tribes, followed by basal leaves rosette forming, fruit orientation, fruit type, and seed wing. Lowest disparity was observed for characters typically fixed across the Brassicaceae with few exceptions, such as stamen number (Supplementary Table 3).

In contrast to overall disparity, we detected phylogenetic signal in disparity for six of the 37 characters analyzed (multicellular glandular hairs, hairs, sepal orientation, flower symmetry, stigma lobing in fruit, and seed wing; indicated in Fig. 2a, b; see Supplementary Fig. 7 and Supplementary Table 3), i.e., in these cases morphological disparity was phylogenetically clustered. All six characters had overall medium disparity values (0.54–0.79), as can be expected for characters displaying differences between clades. While detection of phylogenetic signal is usually used for characters such as morphological traits, where phylogenetic signal indicates non-random distribution of the respective character along the phylogeny, here phylogenetic signal indicates constraints to evolve or reduce morphological diversity in some clades. Five of these six characters also showed high pairwise differences in character state frequency, suggesting that those character states contribute to differences in disparity among evolutionary lineages (Supplementary Table 3).

As we detected differences in tribal level disparity in some characters, but no differences between mean tribal level disparity, we conducted a Discriminant Analysis of Principal Components (DAPC) of disparity with group priors according to the major lineages to assess the characters’ contribution to distinguish between lineages. The three lineages I, II and III were well-separated in the resulting scatterplot (Fig. 2d); however, splitting lineage II further following alternative lineage assignments^26,28^ resulted in overlapping groups (Supplementary Fig. 8). The characters contributing most to separating the lineages mostly coincided with highest pairwise character state difference, phylogenetic signal, or both (Supplementary Table 3), and had medium levels of mean disparity.

### Disparity is a product of a combination of processes

The direct result of diversification processes can be expressed as species or genus richness, and these two numbers may be considered as a good predictor for morphological disparity and realization of the phenotypic space. Accordingly, disparity of tribes (directly calculated from the morphomatrix) was correlated with both species and genus richness (Supplementary Table 8). Tribal crown group age, i.e. the time of diversification, as well as length of the lag-phase between stem and crown group age were also correlated with disparity; however, they were also correlated with species and genus richness, respectively, thus a combination of factors may contribute to disparity.

**Fig. 3:**
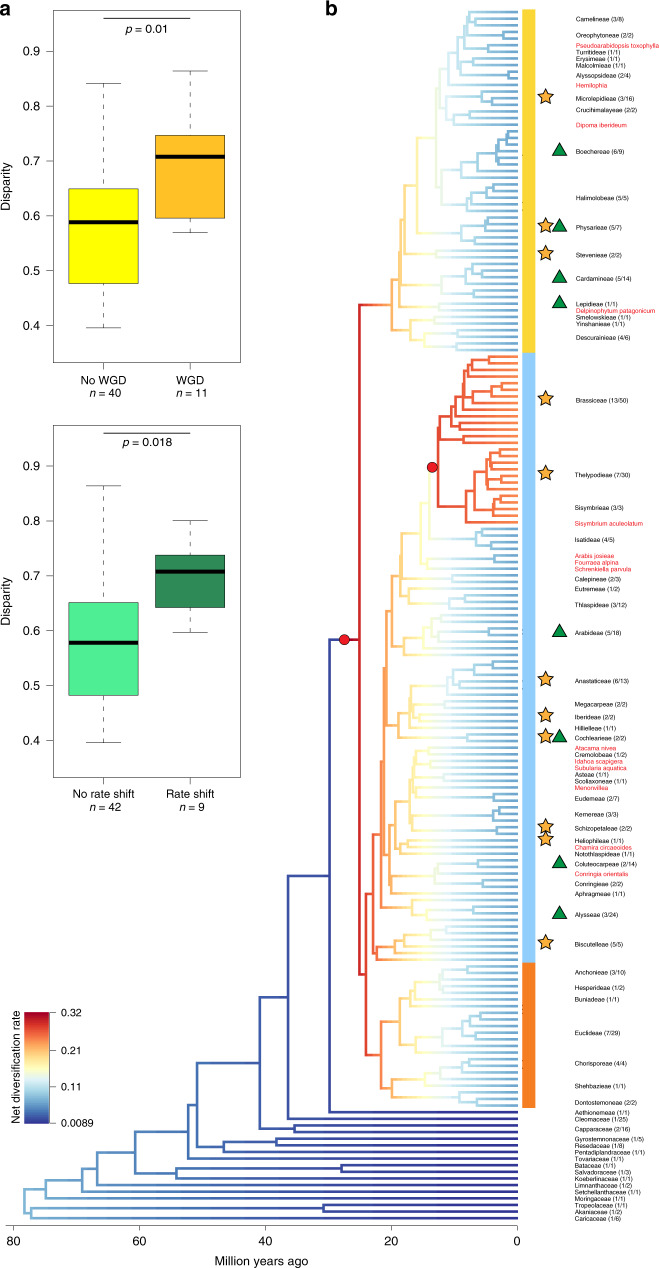
Disparity and diversification. **a** Boxplot of tribal disparity for tribes with and without mesopolyploidizations or rate shifts. Mesopolyploidizations (WGDs) and rate shifts are associated with higher disparity. Significance was tested using phylogenetic ANOVA and *P*-values are indicated above the boxplots. In the boxplot, center line represents median; upper and lower quartiles are indicated by box limits; whiskers represent 1.5× interquartile range and points are outliers. **b** BAMM best shift configuration of genus-level plastome phylogeny. The phylogeny was pruned to contain only one sample per (monophyletic) genus. Two significant rate shifts were detected at the onset of lineage diversification ca. 25 mya as well as before the split of tribes Brassiceae+Thelypodieae+Sisymbrieae from Isatideae, and are indicated with red circles; branch colors indicate net diversification rate. Tribe names, number of genera (sampled/total), mesopolyploidizations^30,31^, species level rate shifts^34^ and lineage assignment are given on the right. Source data are provided as a Source Data file.

It has been debated whether polyploidization is associated with increased diversification rates^41^, and thus may coincide with higher morphological disparity. However, a direct association of polyploidization and increased net diversification has neither been shown for angiosperms^41^ nor for Brassicaceae^34^ or within Brassicaceae (tribe Microlepidieae)^42^. Using phylogenetic ANOVA, we detected significantly higher disparity (*p* = 0.01) in tribes having undergone mesopolyploidization events predating their diversification^30,31^ compared to other tribes (Fig. 3a). These tribes also had a higher number of genera (Supplementary Fig. 9, Supplementary Table 9). Interestingly, while shifts in diversification rates on the species level are not necessarily associated with mesopolyploidization in Brassicaceae^34^, but rather with crown group age and number of species (Supplementary Fig. 10, Supplementary Table 9), disparity is also significantly elevated (*p* = 0.018) in tribes with rate shifts (Fig. 3a).

To bridge previous studies investigating rate shifts within tribes^34^ or in the context of all angiosperms^16^, we conducted a Bayesian Analysis of Macroevolutionary Mixtures (BAMM) on the genus level. We detected two shifts in genus-level diversification rate in the best shift set, both associated with increased net diversification (Fig. 3b). The first shift was located at the base of core Brassicaceae, just before lineage diversification ~25 mya. The second shift predated the split of tribe Brassiceae and the Thelypodieae/Sisymbrieae clade in lineage II 12.6 mya. Shifts at similar branches were detected in the other credible shift sets (Supplementary Fig. 11). The corresponding rate-through-time plot (Supplementary Fig. 12) revealed an increase in net diversification rate until ~16 mya, when many of the tribes originated. Stem and crown group ages of all the tribes are 15.21 ± 4.71 my (mean ± standard deviation) and 9.99 ± 4.77 my, respectively. Correlation analysis furthermore indicated that none of the shifts in speciation rate were significantly associated with genus-level disparity values of any of the 37 morphological characters or mean disparity (Supplementary Table 10).

Diversification rates have been estimated on the tribal level before^34^. We tested whether speciation, extinction and net diversification were significantly different between Brassicaceae tribes having undergone WGDs, rate shifts, a combination of both or neither. As could be expected, net diversification rate was significantly higher in tribes with rate shifts compared to tribes with WGDs or without shifts and WGDs (Supplementary Fig. 13). This could be attributed to speciation rate (Supplementary Fig. 13), where the same significant differences were found. In contrast, extinction rates were not significantly different when corrected for phylogenetic signal, although tribes with WGDs had considerably lower extinction rates compared to those with rate shifts and without WGDs or shifts (0.21 ± 0.19 compared to 0.34 ± 0.38 and 0.32 ± 0.18).

## Discussion

Our analysis of morphological characters and their disparity in Brassicaceae revealed mostly consistent patterns across lineages and traits. Characters with a high mean disparity occur in multiple states across the phylogeny, generally have a low contribution to lineage differentiation and low pairwise differences between lineages, and are devoid of phylogenetic signal. This agrees with previous molecular-phylogenetic studies showing that morphological characters are homoplastic. Characters with low disparity, where one or more of the character states are rare, show a similar pattern of low contribution to lineage differentiation, low pairwise differences and lack of phylogenetic signal. In contrast, those characters showing phylogenetic signal in disparity, high pairwise differences between character state frequencies and high contribution to lineage differentiation have medium levels of disparity. Key innovations after WGDs, such as the emergence of glucosinolates in Brassicales^21^ or the pentamerous flower in Pentapetalae^22^, have been hypothesized to play a major role for subsequent diversification of lineages affected by WGD^43^. Within our extensive set of morphological characters in Brassicaceae, we find no evidence for any morphological character state being a key innovation after mesopolyploidizations, but rather recurrent occurrences of almost all character states and high levels of homoplasy throughout the entire family.

Consistent with the high levels of homoplasy in leaf shape (character D10), we did not detect phylogenetic signal or lineage differentiation in this character. The REDUCED COMPLEXITY (RCO) gene is responsible for the appearance of complex leaves in Brassicaceae^31^, and it has been shown that leaf shape can contribute to fitness in Brassicaceae^44^. Interestingly, this gene originated through gene duplication after divergence from Aethionemeae^36^, providing an example that also local gene duplication can provide the genomic substrate to evolve new traits. This gene has been repeatedly lost across the family^31^, leading to extensive homoplasy. Most trichome types likely evolved multiple times in Brassicaceae^45,46^. The corresponding character in our study is B04 (hairs), in particular character states B04-3 and B04-4. Trichome disparity was not phylogenetically constrained but showed differentiation between lineage I and II. A single origin of malpighiaceous and stellate trichomes is also not supported by our and others’ data^27^, which could be attributed to the complex genetic control of trichome development^47^. Trichome types have not been studied in the context of family-wide diversification rates and species richness, and thougha selective advantage has been discussed^48^, there may also be fitness costs depending on environmental conditions^49^. In contrast, another trichome character (B03, multicellular glandular hairs) was indeed phylogenetically constrained, both regarding disparity and character occurrence. Only four of the seven tribes of lineage III exhibit multicellular glands, making this the only character state restricted to a single lineage. This pattern has been reported earlier^27^, but interestingly our extensive sampling shows that both character states are consistently present at the tribal level and in part even at the genus level, indicating that instead of replacing one character state with another, the potential to exhibit the alternative character state evolved. However, the genetic basis of this trait has yet to be found.

In tribe Brassiceae, taxa with indehiscent fruits have higher net diversification and speciation rates, as well as larger seeds and diverse pericarp features, such as corkiness, hooks, barbs, and wings^50^. Fruit opening in Brassiceae corresponds to our character F27, and we did not find a correlation between its disparity and speciation rates on the genus level (Supplementary Table 10). It is also not significantly phylogenetically constrained (although presence of indehiscent fruit shows a high difference between lineage I and II), but disparity is indeed high in tribe Brassiceae. This is one example that preceding WGD (triplication in Brassiceae^51^) might have increased disparity and net diversification rates. The additional rate shift in Brassiceae+Thelypodieae+Sisymbrieae may have further provided an additive effect and led to an increased speciation rate and high species richness.

Diversification rate upshifts have been intensively studied across angiosperms^12,16^, and some correlation between WGDs and diversification rate upshifts was found, even with a cumulative effect of increased speciation rates with increased number of WGDs in a given lineage^12^. Mean speciation rate in Brassicaceae is about 0.55 species/my^34^, compared to a mean of 0.49 species/my across rosids^12^. However, net diversification rates are highest in the clade containing Brassicaceae, Cleomaceae and Capparaceae compared to all other analyzed angiosperms^16^. An increase in speciation rate after WGDs, as had been observed in other lineages^12^, was not detected within Brassicaceae. In our study, the speciation rate was significantly higher in tribes that underwent rate shifts than in tribes without shifts (Supplementary Fig. 13, Supplementary Tables 11–14), as could be expected, and this is also reflected in elevated net diversification rates. However, this pattern was not observed in tribes with mesopolyploidization events. Net diversification rates were lowest in tribes with WGDs, associated with low extinction rates after WGDs. A decrease in the extinction fraction was also shown in the Brassicaceae+Cleomaceae+Capparaceae clade following At-β in Brassicales^12^. Nevertheless, there are no decreases in net diversification anywhere in Brassicaceae^34^. The robustness of speciation and extinction rates is under debate surrounding the reliability of different widely used methods without further biological information and well-justified constraints^52^ and, therefore, more detailed conclusions are not drawn herein.

Angiosperms underwent on average 3–4 WGDs (on the level of orders and families) over the evolutionary history of land plants, spanning more than 440 my. The Rosids, which Brassicaceae belong to, have a mean number of 4 WGDs^8,12^. Brassicaceae underwent not only the At-α WGD in their early evolution^53^ ~35 mya, but also accumulated eleven additional tribe-specific WGDs (mesopolyploidizations) during their Miocene/Pliocene evolutionary history^30,31^. In total 130 genera are potentially affected by one of the additional eleven mesopolyploidizations, resulting in a mean number of 1.45 WGDs per genus in 35 my. Brassicaceae therefore have a high rate of 0.41 WGD/10 my compared to the land plant average of 0.09 WGD/10 my. If we further consider that nearly 43% of Brassicaceae species are neopolyploids^24^, which are not included in the calculation above, and assume an age of under one million years for the more recent neopolyploids, then a similarly high rate of among recurrent WGDs can also be inferred.

WGDs in angiosperms have been associated with shifts in diversification rate, although mostly after a considerable lag time, leading to the conclusion that WGDs promote, but are not sufficient to cause increased diversification. The generally high and increasing diversification rates have been attributed to the cumulative effect of these ancient WGDs^16^. We also do not observe a strict association of rate shifts with WGDs in Brassicaceae. The lag-time (time span between WGD and associated increased diversification^17^) ranges from 0.6 my to 49.2 my in angiosperms^16^. For the diversification after the At-β WGD event (85 mya^21^ including Brassicaceae with Cleomaceae and Capparaceae) a lag time of at least 36 my can be assumed. At-α was not analyzed, but our data provide a lag time of about 10 my (At-α WGD ~35 mya and core Brassicaceae rate shift ~25 mya). Within Brassicaceae, lag time is in a similar range for the two tribes with both events (12 my and 17 my between an assumed WGD at the base of the tribe and later diversification in Physarieae and Cochlearieae, respectively). This is considerably longer than the mean lag-phase (time span between tribal stem age and first lineage divergence) of about 5 my (Supplementary Figs. 9 and 10, Supplementary Table 2) and highlights the long-lasting impact of WGD on any other factor increasing diversification such as environmental change, migration or coevolution. Our study does not provide further data to explore combinatorial effects of WGD with other factors driving morphological disparity, and we can only summarize earlier findings that lineage diversification and differentiation in Brassicaceae is often associated with major environmental change^18,34,39^.

The herein elaborated phylogenetic framework based on plastome data and the entire rDNA operon has been explored to denote species within tribes and identify those genera, which remain orphan taxa and are not assigned to any tribe. Main evolutionary lineages were defined accordingly. However, phylogenetic relationships among tribes should be regarded as a work in progress and may be further resolved in the future with comprehensive nuclear genome datasets^28,31^ that allow for to the reconstruction of the early evolutionary history of any given tribe. Consequently, our conclusions refer to evolutionary patterns on the level of major evolutionary lineages. However, alternative concepts based on phylogenies derived from the nuclear genome, with additional lineages, were analyzed as well, and resulted in congruent patterns. Furthermore, our conclusions are not based on statistical tests that rely on a resolved genus-level phylogeny (disparity, lineage differences, DAPC). Those tests that use phylogenetic relationships among tribes (phylogenetic ANOVA, test for phylogenetic signal) may be affected if a future comprehensive nuclear genome phylogeny is used and may provide additional significant results. We were not able to reconstruct changes of morphological disparity over time and along lineages since this requires a fully resolved evolutionary history of Brassicaceae incorporating phylogenetic evidence from both plastid and nuclear genomes. This is also a prerequisite for future research studying past environmental parameters driving evolutionary patterns and processes.

We may hypothesize that our findings of high disparity throughout evolutionary times and constantly high net diversification also correspond to constantly evolving genomic blocks as demonstrated extensively in Brassicaceae^54^ both by genomic and cytogenetic analyses^30,55^. The most recent ancestral Brassicaceae genome model consisted of 22 genomic blocks^54,56^. Fractionated blocks are interpreted as a consequence of the diploidization phenomenon in extant paleopolyploids^57^, and evolutionary mobility of genomic blocks is set into a context of frequent polyploidy, diploidization, and block shuffling^55^. This may facilitate future studies to link disparity and trait evolution utilizing entire genomic information^57^, and thereby unravel the phenomenon of consistently high capacity for morphological diversification throughout evolutionary times resulting in a permanent gain and loss of character states. This will also pave the way for new approaches linking phenotypes with genomic sequence and genome context data to detect the underlying genetic causes, as has recently been shown for the genetic basis of leaf shape^31^. Based on our results we propose a model of nested WGDs and diversification shifts triggered by other factors, such as environmental change, which in concert increase morphological disparity.

## Methods

### A matrix of the family-wide morphospace

We generated a morphological character matrix intended to discriminate on genus level. This matrix was generated in two steps. First, the morphospace (characters and character states) used in species and genus descriptions and classical text book determination keys was extracted and incorporated into an ‘online interactive key’ to the genera of Brassicaceae (BrassiBase https://brassibase.cos.uni-heidelberg.de/; toolkit Interactive Key^23^). Data can be fully explored in BrassiBase, and a visualization tool permits a summary on tribal level (BrassiBase; toolkit Morphology Tool^23^). This key was further improved for downstream statistical analysis by adjusting character definition and character states (Supplementary Note 3). Finally, 37 characters with 111 character states were scored for all 351 genera. A detailed definition and description of characters and character states is provided in Supplementary Methods. The morphomatrix integrates data compiled on genus level and does not include individual species’ morphological diversity. Consequently, the morphomatrix represents multistate, unordered characters and character states, and genus (and above) level morphological variation. For downstream analysis, we also scored presence/absence of characters on the tribal level (tribal level morphomatrix).

To assess whether any character states showed differences in their presence/absence across lineages, we calculated the fraction of presence of character states for each lineage from the tribal level morphomatrix. High pairwise differences between lineages indicate character states, which are realized differentially and may be targeted in future studies.

### DNA preparation and sequencing

We selected 178 samples from 176 Brassicaceae species for phylogenetic reconstruction with the aim of (i) covering all tribes, (ii) recovering the deepest split within each tribe, (iii) placing formerly unplaced genera, and (iv) including taxa of interest to the scientific community. Our sampling was further expanded with published data from Brassicaceae taxa and extended to the other families of Brassicales, where we also generated sequences for five new species, thereby covering almost all families. The entire taxon sampling including additional outgroup sequences from the Rosids is provided in Supplementary Data 1.

The Invisorb Spin Plant Mini Kit (STRATEC Biomedical) was used for DNA extractions from dried leaf material of all samples. Preparation and sequencing of total genomic DNA libraries were performed at the CellNetworks Deep Sequencing Core Facility (Heidelberg) with library insert sizes of 200–400 bp either on an Illumina HiSeq 2500 instrument in 100-bp paired-end mode or on a HiSeq 4000 instrument in 150-bp paired-end mode.

### Plastid de novo assembly and analyses

Raw Illumina reads were filtered into high-quality (HQ) sequences using NUCLEAR 3.2.4 (GYDLE Inc., Québec, Canada) by trimming adapters and retaining segments of 50 or more consecutive bases with quality scores 20 or higher. Plastid assemblies were initiated by mapping HQ sequences to the *A. thaliana* plastid genome (ENA/GenBank accession NC_000932.1; [https://www.ncbi.nlm.nih.gov/nuccore/NC_000932.1]), using NUCLEAR parameters: -l 40 -s 16 -m 3 --min-score-cov 80, thus selecting fragments aligned over at least 80% of one of their sequence reads in High-scoring Segment Pairs (gapless local alignments) of 40 bases or more, with 16 consecutive identities and at most three mismatches every 40 bases (92.5% local similarity). The assemblies were then produced using VISION 2.6.12 (GYDLE Inc., Québec, Canada) by iterative resolution steps combining local fragment realignment, consensus resolution, gap filling, segmental reorganization, recruiting and remapping of HQ sequences, interactive edition and visualization. The assembler in VISION is always aware of each fragment’s mapping scores, therefore regions consistently connected and covered by perfectly aligned reads are not subject to misassembly influenced by additional divergent reads, such as those carrying sequencing errors or representing contaminants or insertions into the nuclear genome. The orientation of the small single copy (SSC) region relative to the large single copy (LSC) region was kept consistent between all samples, and the finishing process ensured the proper assembly of identical inverted repeats and their exact junctions to the LSC and SSC regions, with all assemblies starting at the first base of the LSC.

Plastid annotation was performed using a curated set of plastid gene features extracted from *the A. thaliana* plastid genome GenBank record. These features were aligned to the assemblies in their nucleotide form with NUCLEAR, CDS (coding sequences) were also aligned in their amino-acid form with BLASTX. Protein and nucleotide alignments were processed in order to adjust start and stop codons and detect pseudogenized genes due to missing exons or internal stop codons.

### Alignment and phylogenetic analysis

Geneious 7.1.7 (Biomatters) was used for the extraction of plastid coding regions (excluding introns), which were subsequently aligned using the FFTNS-ix1000 algorithm in MAFFT 7.017^58^ as implemented in Geneious, with the 200 PAM/k = 2 scoring matrix, a gap open penalty of 1.53, and an offset value of 0.123. Starting from the set of plastid genes used for phylogenetic reconstruction of Brassicaceae earlier^24^, we selected genes based on available annotation in our extended taxon set. In general, only genes present in all taxa were considered for phylogenetic data analysis, yet in order to maximize available sequence information within Brassicaceae, two exceptions were made for genes *rrn*16 (alignment position 25,102–26,517, 1416 bp) and *trn*F-GAA (alignment position 28,456–28,519, 64 bp) as these genes were missing only in one outgroup sample respectively, namely in *Tovaria pendula* (*rrn*16) and *Setchellanthus caeruleus* (*trn*F-GAA) from the Capparales. Here, missing sequences were replaced by Ns. A total of 60 genes remained, including 43 protein-coding genes, 14 tRNAs and three rRNAs. Start and stop codons were removed from alignments of protein-coding genes and all alignments were realigned (settings as above). After manual inspection of all sequence alignments, Gblocks 0.91b^59^ was used to exclude indels using a minimum block length of 2 bp and also saving non conserved blocks given the high sequence divergence in the dataset. The remaining data blocks were concatenated in Geneious resulting in a total alignment length of 29,126 bp with 12,187 variable sites (9326 thereof parsimony informative).

PartitionFinder 2.1.1^60^ was used to determine the best-fit partitioning scheme in order to account for putative rate heterogeneity among the selected genes. Models implemented in BEAST were tested using the Bayesian Information Criterion (BIC) for model selection in a greedy search and branch lengths were allowed to be unlinked. A single partition was determined and GTR + G + I + X was identified as the best substitution model. The analysis was performed twice to confirm the resulting partitioning. Both analyses revealed the same best-fitting partitioning scheme, which was therefore used in the following analysis and is in congruence with earlier analyses^39^.

RAxML 8.1.16^61^ was used to conduct an unpartitioned maximum likelihood (ML) phylogenetic tree reconstruction with a rapid bootstrap analysis (1000 replicates) and search for the best-scoring ML tree under the GTR + G + I + X model. *Vitis vinifera* was set as outgroup. The resulting phylogenetic tree was compared to a phylogeny based on the entire nuclear encoded rDNA operon (see Supplementary Methods for details).

### Divergence time estimation and fossil calibration

In order to use the ML tree as a starting tree for divergence time estimation, the R package ape 3.1–4^62^ was used to make the ML tree ultrametric with node ages corresponding to time constraints using the ‘chronos’ function. Divergence time estimation was performed in BEAST 1.8.4^63^ under an uncorrelated lognormal relaxed clock approach with estimated rates and *Vitis vinifera* set as outgroup. The BEAST analysis was performed following Hohmann et al.^24^ with four fossil calibration points (Supplementary Table 16) using uniform distributions and a maximum age of 125 mya for all fossil constraints. The root age was bounded between 92 and 125 mya with a uniform distribution.

Twenty-eight independent MCMC runs were performed with 100,000,000 generations each and sampling parameters every 50,000 generations until the effective sampling size (ESS) of each parameter in the combined logfile exceeded 200 excluding the first 20% of generations as burn-in. We used LogCombiner 2.4.7^64^ after removing burn-in to combine trees from the different runs. A maximum clade credibility tree was constructed from 21,038 trees in TreeAnnotator 2.4.7^64^ and visualized using FigTree 1.4.1^63^.

### Morphological disparity analysis

Since the morphological variation (characters and character states) was defined and scored reflecting the entire discriminative morphospace of Brassicaceae, the same dataset was used to study morphological disparity in more detail. Morphological disparity, the ‘range or variance of morphological form across species or other taxa’^2^ is here defined as the fraction of character states per character, which are realized on a given taxonomic level. Consequently, the disparity value for each character ranges from one divided by the number of character states (only a single character state realized) to one (all character states realized).

For downstream analyses, both genus and tribal level disparity was used, and we provide two different values for tribal level disparity. (i) Disparity was calculated directly from the tribal level morphomatrix (‘disparity direct’). (ii) Mean tribal disparity was calculated from genus-level disparity values (‘disparity from genera’). Direct disparity values are never lower than disparity from genera. Correlation of the two matrices was estimated using Spearman’s rank correlation coefficient in R. Additionally, we searched for phylogenetic autocorrelation using Moran’s I^65^ within disparity for all 37 characters and mean disparity on tribal level (direct calculation) with the R package phylosignal 1.2.1^66^ using a pruned tribal tree based on our BEAST results.

### Analysis of diversification patterns

Patterns of diversification were assessed using Bayesian Analysis of Macroevolutionary Mixtures 2.5.0 (BAMM^67^) on the genus level. We first pruned our dated phylogeny to include only one species per (monophyletic) genus within Brassicales. Multiple tips were retained in genera displaying paraphyly in the plastome BEAST phylogeny. Genera missing from the phylogeny were accounted for by using clade-specific sampling probabilities on a tribal level within Brassicaceae and, because the tribal system is specific to the more species-rich Brassicaceae, on a family level in all other Brassicales families. Number of genera for Brassicales families were extracted from the Angiosperm Phylogeny Website^68^. Rate parameters were detected using the ‘setBAMMpriors’ function in the R package BAMMtools 2.1.6^69^ from the input tree and total number of genera (425 including outgroups), and the prior for the expected number of shifts was set to 1.0. BAMM was then run for 10,000,000 MCMC generations with default MCMC settings, and convergence of runs was detected using the log-likelihood trace as well as determining effective sample size (ESS; > 200) of the log-likelihood and number of shifts after discarding the first 1,000,000 samples as burn-in using the R package coda 0.19–2^70^. The credible shift set and best tree configuration were determined using the respective functions in BAMMtools.

Finally, we correlated disparity values (calculated on the genus level and for each of the 37 characters separately as well as for mean disparity) and speciation rates with the function ‘traitDependentBAMM’ implemented in BAMMtools using the spearman correlation coefficient and a two-tailed test. We subset the BAMM results using BAMMtools ‘subtreeBAMM’ to exclude outgroups, where no disparity values were available.

### Disparity pattern across phylogenetic groups

In order to further explore the morphological disparity, a Discriminant Analysis of Principal Components (DAPC^71^) was carried out using the ‘dapc’ function implemented in the R package adegenet 2.0.1^72^. By maximizing variation between groups while minimizing variation within groups, the DAPC, although initially developed and applied for the analysis of genetic clusters, was utilized in order to reveal—if present—clustering patterns within the disparity data and to identify the morphological characters contributing most significantly to these patterns. The analysis was based on the direct estimate of tribal level disparity. Since DAPC is based on an a priori grouping of the dataset, we used the ‘find.clusters’ function to identify putative clusters within the disparity data, yet no optimal grouping solution could be revealed from the resulting BIC values. Therefore, two DAPCs were performed with the major lineages set as group priors, both with basal tribe Aethionemeae and without. For both DAPCs, cross-validation was performed using the ‘xvalDapc’ function (with 1000 replicates for each level of PC retention) in order to identify the optimal number of principal components (PCs) to retain based on the lowest root mean squared error associated with the predictive success.

### Calculation of stem and crown group ages and lag time

The comprehensive plastome dataset included taxa from all tribes. Our initial taxon sampling aimed to cover the deepest split within every tribe to allow for comparison of this respective node age (crown group age) with the onset of the tribal lineage (stem group age). Since we sampled all tribes, all stem group ages could be extracted from plastome-based divergence time estimates (Supplementary Table 2, Supplementary Fig. 2). The plastome dataset furthermore allowed us to extract tribal crown group ages for 39 tribes, because (i) we included taxa representing the deepest node in the respective tribes and (ii) there is phylogenetic congruence with published data (see Supplementary Note 1). For estimation of the crown group age of tribe Camelineae we excluded *Pseudoarabidopsis* (see Supplementary Note 2). For tribes with one single genus only, such as Shehbazieae and Aphragmeae, there is no crown group age.

In order to generate an almost complete dataset for tribal crown group ages we extracted time estimates from an ITS-based analysis, which considered more than 2000 Brassicaceae species for crown group age estimation:^34^ This study had calibrated individual ITS-based tribal trees (including a consistent set of outgroups among trees) with multiple plastome-based divergence time estimates^24^. These divergence time estimates used for secondary calibration^34^ are fully consistent with those we present here. We first compared our herein estimated crown group ages based on plastome sequences with those from Huang et al.^34^. This direct comparison of crown group ages of tribes was possible in 24 cases and was used to calculate a linear regression and calibration line comparing plastome and ITS derived datasets (Supplementary Table 2, Supplementary Fig. 4). This regression line was used to extrapolate for missing crown group ages in our study to maximize (1) consistency among datasets and (2) work with a complete stem/crown group age dataset for further statistical tests. Since the regression between both studies was nearly perfect, we added missing crown group ages for 12 tribes from Huang et al.^34^ to our dataset (Supplementary Table 2).

A group of tribes closely related to Cremolobeae (eight species) (namely Asteae, Scoliaxoneae, Eudemeae-with only 31 species in total) was not fully resolved according to their tribal assignment, neither in BEAST nor in ML analysis, indicating a polytomy (Supplementary Fig. 1/2). Therefore, we combined all four of them into one group and extracted one single crown group age for all of them. This close relationship has been discussed earlier^73^.

Finally, we calculated three values related to the estimated divergence time for all tribes. (1) Mean stem group age, which is solely based on herein presented plastome data, (2) Mean crown group age, which is mostly based on herein presented plastome data and supplemented by estimates from Huang et al.^34^ as described above, (3) ‘Lag-phase’, representing the time between respective stem and crown group ages and indicating a time span of evolutionary stasis (here with zero net diversification).

### Association between tribal level data

Finally, we assessed the correlation of tribal level disparity, genome size, species/genus richness, and stem/crown group age as well as lag-phase with each other and their association with the presence of mesopolyploidization events^30,31^, significant shifts in diversification rate detected in the ‘best shift configuration’^34^ and with assignment to the major evolutionary lineages^25,26,28^. Mesopolyploidization events were found at or near the origin of eleven tribes (excluding the WGD detected in *Leavenworthia*, Cardamineae, as it is restricted to this genus and not shared with the rest of the tribe)^30,31^ and shown to be absent in at least twenty tribes^31^, most notably in all analyzed tribes of lineage III^32^. For a number of other tribes, low base chromosome numbers and genome sizes^24^ are consistent with a lack of mesopolyploidization, while for few, neither data nor material for cytogenetic or sequence-based analyses is available (Supplementary Table 1). We also assessed differences in tribal speciation, extinction and net diversification rates^34^ (shown in Supplementary Table 16) depending on occurrence of mesopolyploidization, rate shift, both, or neither on tribal level. Rate estimates calculated under a Bayesian framework using BAMM^67,69^ were obtained from a recent Brassicaceae-wide study^34^. Mean tribal genome size values were recalculated with up to date taxonomic information and tribal assignments based on previously published data^26^, and genome size variation was calculated as the standard deviation of genome size estimates for a given tribe normalized by its mean while restricting to tribes with genome size data available for at least three taxa. For correlation of tribal level data, we used Phylogenetically Independent Contrasts^74^ in R (‘pic’ from the package ape) to account for phylogenetic dependencies. To test whether tribal level data was significantly different between those tribes having undergone mesopolyploidization events or rate shifts, we used phylogenetic ANOVAs^75^ with 1000 simulations and posthoc comparisons and Bonferroni correction using ‘phylANOVA’ from the R package phytools 0.6–60^76^. The same analysis was conducted to test for differences of diversification rates; the detected rate shift was in Brassiceae+Thelypodieae+Sisymbrieae was considered here. Finally, to test for significant differences between the lineages, where data are per se highly phylogenetically dependent, we used Kruskal–Wallis rank-sum tests followed by pairwise Wilcoxon rank-sum tests with Bonferroni correction when a significant difference was detected.

### Reporting summary

Further information on research design is available in the Nature Research Reporting Summary linked to this article.

## Supplementary information

Supplementary Information

Peer Review

Reporting Summary

Description of Additional Supplementary Files

Supplementary Data 1

## Data Availability

Data supporting the findings of this work are available within the paper and its Supplementary Information files. A reporting summary for this Article is available as a Supplementary Information file. The datasets generated and analyzed during the current study are available from the corresponding author upon request. The plastome sequences generated during the current study are available at ENA/GenBank under accession codes MK637648 -MK637830 (see Supplementary Data 1), and raw sequencing data under ENA/GenBank Bioproject PRJEB38700. The taxonomic and morphological datasets as well as alignments and the dated phylogeny are available at Dryad [10.5061/dryad.fttdz08pt]. Source data are provided with this paper.

## Source data

Source Data
