# Supplementary material for: Nested whole-genome duplications coincide with diversification and high morphological disparity in Brassicaceae
Source: Nat Commun. 2020 Jul 30;11:3795. doi: 10.1038/s41467-020-17605-7 (PMC7393125; doi:10.1038/s41467-020-17605-7)
Supplement: Supplementary file 1 — Supplementary Information [file 41467_2020_17605_MOESM1_ESM.pdf]

Nested whole-genome duplications coincide with diversification and high morphological disparity in Brassicaceae

Walden *et al*.

Supplementary Note 1. Taxon sampling, evaluation of tree topologies within and among tribes–a literature survey

For taxon sampling in our study any relevant literature was consulted to select an appropriate set of species (see Supplementary Note 3). Furthermore, we also included many taxa from formerly phylogenetically unplaced genera as well as, in our opinion, taxa of broader interest to the scientific community and checked any material accessible (herbarium voucher, germ plasm collections, living collections, Supplementary Note 3). Previous family level phylogenetic analyses of Brassicaceae have been criticized because outgroup selection did not include the entire order Brassicales. This was considered here, and Brassicales are included with representatives from almost all families[1] (only Emblingiaceae are missing) plus a representative set of the rosid superorder[2]. The sampling also aimed at representing the deepest splits within any of the analyzed Brassicaceae tribes to allow subsequent calculation of tribal stem and respective crown group ages (for detailed accession data refer to Supplementary Data 1).

Here, we compare our plastome-based phylogeny with previous relevant studies. However, it should be noted that there is no comprehensive (family-wide) and at the same time highly resolved tree available yet fulfilling any requirements for a reliable phylogeny (multiple outgroups, multiple ingroup taxa at any taxonomic level, avoidance of undersampled regions within a given tree).

The angiosperm-wide context of the herein presented phylogenetic framework defining major important evolutionary lineages is best reflected by a comparison of the following contributions, some of them also considered a dense sampling from order Capparales, to which Brassicaceae belongs to:

Edger *et al*.[3] studied the order Brassicales and the respective butterfly-plant arms race. This study did not include taxa outside Brassicales and species sampling within Brassicaceae was low (six species), but the study allows for comparisons concerning the phylogenetic relationships of the various families from the entire order. The phylogenetic findings were consolidated further in another contribution focusing on plastome-wide sequence data[1]. An angiosperm (rosid)-wide Brassicaceae phylogenetic context has also been presented four years ago[2], but this contribution missed various families from the order Capparales. Increased Brassicaceae-wide sampling was presented by Guo *et al*.[4] building upon the plastome dataset presented earlier[2]. The latest two important contributions increased sample sizes: The study presented by Nikolov *et al*.[5] focused on Brassicaceae and also included a plastome-wide analysis, but this study again neither considered the entire Brassicales nor a wider angiosperm/rosid context. This is further complemented by Li *et al*.[6] studying plastome data from more than 85% of all angiosperm families, but using a restricted number of Brassicaceae taxa. In summary, our herein presented comprehensive plastome-based data set is fully congruent on the tribal level with any significant grouping presented in previous studies also based on plastome data.

The only three studies[5,7,8] serving as starting point for an evolutionary framework for Brassicaceae derived from the nuclear genome studying hundreds of genes progressively increased sample size. With these analyses the structure of the phylogenetic hypotheses changed gradually according to delimitation of the major lineages I, II, and III sensu Koch & Al-Shehbaz[9] and Franzke *et al*.[10]:

Kagale *et al*.[7]: 23 species, 19 genera, 213 genes [(III,(II,I)]

Huang *et al*.[8]: 55 species, 45 genera, 113 genes [(III,(II,I)], with further subgrouping of II into B, C, and D.

Nikolov *et al*.[5]: 79 species, 72 genera, 1827 exons [(III,(IV),(I,II)], with subgrouping of II into II, IV and V similar to Huang *et al*.[8].

However, all these analyses are still biased because of undersampling (often only one representative per tribe was selected), and in all cases outgroup selection did not include a broad sampling of the order Capparales. Strategy of selecting and defining 'single/low copy orthologues' is different among all three studies, and there is no unambiguous grouping of basal main lineages with high statistical support in any of these three analyses.

Aside these three studies, which are most likely paving the way for a future 'nuclear genome perspective', there is a relatively short history of unravelling molecular-phylogenetic relationships of Brassicaceae on the family level and below. The first benchmark was set by Bailey *et al*.[11] using the internal transcribed spacer of nuclear encoded ribosomal DNA (ITS1 and ITS2) and including a large number of taxa from the entire Brassicaceae family. This was followed by super-matrix approaches based on few markers including ITS, and overlapping, but not necessarily identical taxon sets[12]. However, due to various marker-specific uncertainties (orthologue-paralogue problem, multiple loci and respective pseudogenes, non-concerted versus concerted evolution)[13] it became obvious that this molecular marker did not reliably resolve on the family and above-tribal level. However, ITS sequence data largely framed our understanding of monophyletic groups of genera (combined into tribes). ITS based results successfully identified monophyletic groups of genera, thereby often replacing 'traditional' and solely morphology-based tribal circumscriptions. As a consequence, most tribes have been newly circumscribed, with currently 51 tribes recognized within the most up-to-date concept[14]. During the past ten years, a few major results were found consistently among studies and genomes analyzed and should be listed here:

(1) Tribe Aethionemeae is sister to all other Brassicaceae lineages and tribes[15–17].

(2) The entire Brassicaceae (excluding Aethionemeae) can be further divided into three major evolutionary lineages I, II, and III[2,9] with lineage II split into a core clade II and a large phylogenetically unresolved group of rapidly evolved tribes (expanded lineage II)[10,18]; or 'B, C, D'[8], or 'II and V'[5]. In the study of Nikolov *et al*.[5] a new lineage IV was separated from lineage II and appeared as a sister group to lineage I and II,

but none of the basal nodes separating main evolutionary lineages are universally supported by all concatenation and coalescence analyses performed[5].

(3) All three major evolutionary lineages evolved along an often weakly resolved polytomy[12,16,18]. However, organellar data from entire plastome sequence data[2,4] indicated a highly significant phylogenetic grouping: [Aethionemeae,(I,(II,III))], whereas a datasets based on multiple nuclear genes indicated a different but also (less) significant topology: [Aethionemeae,(III,(II,I))][8]. The reasons are still unclear (e.g. incomplete lineage sorting of organellar genomes, plastid capture, combing orthologues and paralogues from nuclear genomic datasets due to extensive polyploidization); but in both topologies main lineages diverged within a comparatively short time period. Since Nikolov *et al*.[5] did not provide any divergence time estimates this study cannot fully be compared with other studies estimating time divergence.

Our herein presented large-scale plastome-based phylogeny shows the highest bootstrap support among all major lineages as well as among tribes and is fully consistent with the recognized [Aethionemeae,(I,(II,III))] topology (Supplementary Figure 1). Thus, we consider our phylogeny as the momentary best available 'maternal perspective on Brassicaceae evolutionary history'. We are aware that this is not the ultimate 'species tree', but it is the most comprehensive analysis defining tribes and lineages. Therefore, our analysis is not flawed by taxonomical problems: e.g. in the study of Nikolov *et al*. [5] tribe Stevenieae was erroneously placed along with tribe Arabideae, because the authors include as sole taxon *Pseudoturritis turrita* and neither *Stevenia* (giving the name of the tribe) nor *Macropodium* was analyzed, which would have most likely resulted in a correct placement within lineage I.

Previous phylogenetic analyses generally did not focus on relationships between tribes (except Nikolov *et al*.[5]), therefore we compared our plastome dataset at the tribal level with published data at the level of tribes and groups of genera. We compared the topologies within tribes inferred from our whole-plastid genome sequence data with findings of recent and relevant molecular phylogenetic studies, with a focus on plastid data. For the following 15 tribes, a topological comparison is not meaningful on tribal and genus level because tribes either consist of one genus only with 3 or less species, such as: Asteae (2 species), Bivonaeeae (1 species, to be integrated into Brassiceae), Buniadeae (2 species), Kernereae (3 species), Notothlaspideae (2 species), Scoliaxoneae (1 species), Shehbazieae (1 species), Turritideae (2 species); or they consist of one or two genera with low species number (genera, species): Aphragmeae (1, 14), Conringieae (2, 9), Hillielleae (1, 10), Schizopetaleae (2, 17), Iberideae (2, 30), Malcolmieae (1, 6), Stevenieae (2, 10; excl. *Pseudoturritis*). Tribe Erysimeae is an exception, consisting of one genus but comprising 274 species. For three additional tribes our sampling (2 representative taxa) is not sufficient to be compared with other studies, because of inaccessible material [Alyssopsideae (5, 9), Calepineae (3, 9), Eudemeae (8, 30)].

The remaining 32 tribes are systematically complex and have been evaluated accordingly. Topological congruence between our plastome tree and previously published phylogenetic hypotheses was proven for 20 tribes: Aethionemeae[19],

Alysseae[20], Anastaticeae[16], Anchonieae[21], Boechereae[22], Brassiceae[23], Camelineae[15,24], Cardamineae[25], Chorisporeae[26], Coluteocarpeae[27,28], Descuraineae[29], Eutremeae[30], Halimolobeae[31], Heliophileae[32], Isatideae[33], Lepidieae[34], Microlepidieae[35], Thelypodieae[36–38], Thlaspideae[39] and Yinshanieae[40].

Comparison of some of our intra-tribal topologies representing five tribes with previously published phylogenies was difficult due to incommensurable taxon sampling, but general clade and group recognition is congruent: Tribes Cochlearieae[41], Hesperideae[42], Megacarpaeeae[43], Oreophytoneae[35] and Physarieae[44].

And finally, mostly minor discrepancies were found between our findings and earlier phylogenetic analyses as for topologies within eight tribes: Arabideae[45], Biscutelleae[46], Euclidieae[42,47], Dontostemoneae[48] and Smelowskieae[49]. In these cases, incongruences are mostly due to different generic circumscription(s) (e.g., *Arabis*) and/or single species not consistently placed in respective genera (e.g. *Clausia aprica*). In (our) tribal level context, noteworthy differences between our tree and previous analyses apply to tribes Cremolobeae, Eudemeae, and Schizopetaleae. In contrast to our findings, these tribes appear monophyletic in Salariato *et al*.[50]. However, monophyly of tribe Cremolobeae and sister group relationship of tribes Cremolobeae and Eudemeae are only weakly supported in Salariato *et al*.[50]. Tribe Sisymbrieae also appears not monophyletic in our tree with *Sisymbrium aculeotum* being sister to a clade comprising tribes Sisymbrieae and Thelypodieae, respectively. This is in contrast to findings of Warwick *et al*.[51], demonstrating, though weakly supported monophyly of tribe Sisymbrieae.

In our study we did not include sequences of *Bivonaea*, because DNA extractions for genome skimming failed, but Warwick *et al*.[52] demonstrated a sister relationship of *Bivonaea* with *Horwoodia* within tribe Brassiceae. Herein, we successfully integrated plastome data from *Horwoodia*, which is also clearly nesting within tribe Brassiceae (Supplementary Figure 1). Consequently, Bivonaeeae as monospecific tribe is fully nested within Brassiceae. A similar finding was also presented by Nikolov *et al*.[5], and we tentatively will not recognize Bivonaeeae as a separate clade until the detailed evolutionary history of this phylogenetically difficult and highly polyploid lineage has been solved.

The analysis of the entire nuclear encoded rDNA operon (18S, 5.8S and 26S rRNA) excluding the highly variable internal transcribed (ITS1 and 2) and non-transcribed spacer (NTS) was performed to test for phylogenetic signatures focusing on the tribal level and to further highlight comparisons among datasets from plastid and nuclear genomes. The alignment was 5,362 bp in length (number of variable/parsimony informative sites for 18S: 180/116, 5.8S: 24/13, and 26S: 696/487). The corresponding RaxML tree (Supplementary Figure 3) is not able to resolve significantly between evolutionary lineages and tribes, and bootstrap values are low across the tree. This can be expected, since phylogenetic based on rDNA operon intervening ITS1 and IT2 regions are suitable for phylogenetic reconstructions within defined tribes (see the extensive literature survey in this Supplementary Note), but

largely failed to identify significantly relationships among tribes and main evolutionary lineages (e.g. Bailey *et al.*[11]).

However, the general structure of the tree using the coding regions from the entire rDNA operon provided more information compared to earlier ITS studies. Aethionemeae is consistently placed sister to all Brassicaceae, and placement of lineage I as sister to combined lineages II and III is in agreement with phylogenetic hypotheses derived from the plastid genome (though bootstrap support is low). This finding might indicate a strong maternal effect, which may have also influenced the evolution of the rDNA operon subjected to concerted evolution[53] to one of the parental types, most often that of the maternal parent[54,55]. Accordingly, phylogenetic analyses using nuclear gene sets demonstrate the alternative relationship among lineages I, II and III. Pairwise comparisons of our plastome phylogeny, the rDNA tree and a recent phylogenetic tree based on nuclear genome data[5] is shown in Supplementary Figure 14.

We may consider this as first and preliminary evidence that lineage II might be of an old introgressive origin or subjected to massive geneflow between lineage III (maternal) and lineage I (paternal), which may have also influenced subsequent diversification in lineage II resulting not only in an increased number of monophyletic groups defined as tribes, but also in increased percentage of mesopolyploid tribes per evolutionary lineage (31% in lineage II compared to 18% and 0% in lineages I and III, respectively).

The rDNA tree also shows a number of tribes being split (either on tribal level or being assigned to a different evolutionary lineage, though with low bootstrap support), which we also may consider as signature of concerted evolution following reticulate evolutionary processes and/or polyploidization events: Iberideae, Microlepidieae, Stevenieae, Anastaticeae, and Biscutelleae all underwent a mesopolyploidization. The split separating analyzed species from Descurainieae and Chorisporeae is fully congruent with infratribal structure as revealed by plastome data. This further highlights plastid capture and reticulate processes either within or even between tribes as described earlier for *Pachycladon* from tribe Microlepidieae[56]. In the case of Camelineae this phenomenon has been identified and described earlier[57], and it was shown that the conflicting phylogenetic structure is the result of biased gene retention in the face of massive nuclear introgression. Two other cases are affecting single species, namely *Hilliella paradoxa* (high polyploid[58]) and *Pseudoturritis turrita*, and both are known for phylogenetic uncertainties in earlier studies[40,59,60].

In summary, the rDNA operon provides additional valuable and congruent information highlighting the complex evolutionary history of Brassicaceae, which is

largely driven by hybridization, introgression and polyploidization. The results may also indicate that there is no simple tree-like visualization of the evolutionary history of the entire Brassicaceae family, and future phylogenetic research has to elaborate on the various evolutionary processes resulting in the various conflicting signals rather than trying to reconstruct a single ultimate tree relying on cladogenetic evolutionary processes.

Supplementary Note 2. The placement of remote and yet unassigned taxa – a comprehensive treatment

During the past years most genera of Brassicaceae have been aligned with particular tribes. As a consequence, 52 defined tribes have been considered as monophyletic lineages, one of which, Bivonaeeae, we do not recognize anymore, and thereby reducing the number of accepted tribes to 51.

However, a number of genera have not been assigned to any tribe yet for various reasons:

(1) no material was accessible and therefore they were not analyzed molecular-phylogenetically.

(2) DNA sequence information was limited and phylogenetic reconstructions were not able to identify relationships significantly.

(3) phylogenetic inferences, e.g. based on plastid versus nuclear DNA sequence information, are contradicting and indicate reticulate evolution not allowing simple assignment without further arguments (e.g., based on morphology).

In the following we comment on any genus with unclear earlier assignment and make suggestions for its taxonomic placement. It should also be mentioned here that in the past the term 'unassigned genera' was often misused as an indicator that there is no information about phylogenetic placement (see point 3, above). Therefore, we will use this term only for situations as indicated under points 1 and 2. In contrast, genera that cannot be significantly assigned to any tribe because of high genetic distance are defined as 'remote genera'.

Altogether, 21 genera belong to this group of unassigned and remote genera. Considering a total number of 351 genera, this is less than 6% and therefore of minor importance for family-wide conclusions based on tribe-level grouped information. It also has to be noted that any of these 21 genera can be grouped with evolutionary lineages I, II (expanded II), or III without any doubt. Finally, nine of these 21 genera have been assigned to tribes, reducing the number of unassigned and remote genera to 12 (3.4%). Species number in these 12 genera is 53 only, of which 45 represent genus *Menonvillea* (1.3% of total species number of Brassicaceae).

Six genera are considered 'remote', which are *Asperuginoides*, *Fourraea*, *Hemilophia*, *Idahoa*, *Schrenkiella*, and *Subularia*.

Asperuginoides was found to be related to Cochlearieae[21], but no close affinity was detected in other studies[43,52]. Also, the herein presented plastome data do not indicate a close affinity to any recognized tribe.

Fourraea has long been known to be a remote taxon of lineage II[16,43,50]. Our plastome analysis confirms this, and, highlights that also *Arabis josiae* should be placed close to *Fourraea* as remote taxon, which makes it necessary to introduce a new monospecific genus.

Hemilophia is positioned within lineage I[16], and this conclusion is fully consistent with our current plastome analysis.

Idahoa and its phylogenetic position was unclear so far[16,52], but relationship certain affinity to *Subularia* was suggested. Our plastome data fully confirm this.

Similarly, *Subularia* and its phylogenetic position was also unclear and differs among authors[16,52], but as outlined above *Subularia* seems to be related to *Idahoa*[52] and this is clearly confirmed here.

Schrenkiella is also placed at the base of lineage II in our analysis, similar to *Fourraea*. The remote status of this genus has also been shown in previous analyses[8,43].

The following nine genera could be newly assigned to tribes in our study in agreement with interpretations of previous phylogenetic studies, which are *Bivonaea*, *Horwoodia*, *Ochthodium*, *Petrocallis*, *Pseudofortuynia*, *Pseudoturritis*, *Raphanorhyncha*, *Sinalliaria*, and *Veselskya*.

Bivonaea was shown earlier to group within tribe Brassiceae and to be closely related to *Horwoodia*[52]. Our data fully confirm this.

Horwoodia should also be placed within Brassiceae (own plastome data). This was also shown earlier[52] by grouping *Horwoodia* with Brassiceae and demonstrating close relationship to *Bivonaea*.

Ochthodium is now assigned to tribe Sisymbrieae. This is fully supported earlier evidence[52].

Petrocallis can be assigned to tribe Kernereae.

Pseudofortuynia can be assigned to tribe Sisymbrieae. Additional evidence has already been presented earlier[61], and has recently resulted in a taxonomic revision[62].

Although *Raphanorhyncha* was not included in our analysis, morphological variation clearly indicates its assignment to tribe Thelypodieae. This is also suggested by the analysis of Nikolov *et al*.[5].

Pseudoturritis and its clear position within Arabideae (plastome data presented herein) contrasts with a previous placement in Stevenieae[59]. However, recent genome-wide analyses demonstrated also a basal position of tribe Arabideae[5,63].

Sinalliaria was placed in tribe Brassiceae[64], which was confirmed here.

Veselskya is placed in tribe Anchonieae. This is supported by morphology, but also diagnostic barcode markers (ITS1-2: internal transcribed spacers 1 and 2 of nuclear ribosomal DNA; plastid *trn*LF region; submitted for this study, ENA/GenBank codes MH718328, MH720340, MH720341).

The following genera remain unassigned. Most of them might be the result of ancient reticulate evolutionary processes. There are *Atacama*, *Chamira*, *Delpinophytum*, *Dipoma*, *Menonvillea*, and *Pseudoarabidopsis*.

Atacama has been segregated from *Mathewsia* (Schizopetaleae) without resolving its tribal position[65]. This genus definitely belongs to the South American 'CES' (Cremolobeae + Eudemeae + Schizopetaleae) assemblage[66], but a more detailed assignment is not possible.

Chamira shows close relationship to *Heliophila* (93% bootstrap support in our data) and has been debated for a long time[67]. Phylogenetic placement results differ among authors and do not confirm unambiguous affinity to Heliophileae[16,52]. One reason for this could be the mesopolyploidization event at the base of tribe Heliophileae[68].

Delpinophytum also represents a long-known taxonomical and phylogenetic problem[52]; based on discrepancy between nuclear and chloroplast markers[69] an intertribal origin within lineage I (Lepidieae as maternal clade) could be assumed.

Dipoma shows a close relationship to Crucihimalayeae based on plastome data (99% bootstrap support in our data), but this is not in congruence with previous results[52] based on the nuclear encoded ITS.

For *Menonvillea* our data do not support a position within Cremolobeae[50,52,69]. However, the position within Cremolobeae generally lacks support[70].

Pseudoarabidopsis is clearly placed within tribe Turritideae based on our plastome data (100% bootstrap support). But this disagrees with previous studies placing it within Camelineae[43,52]. Interestingly, if we compare phylogenetic reconstructions using the ITS data on family level, we also obtain an intermediate position between both tribes[71].

Finally, we identified one single species, *Arabis ottonis-schulzii*, which does not belong to tribe Arabideae (genus *Arabis*) and has to be integrated into tribe Conringieae[72] under a new generic circumscription[73]. Vice versa, *Conringia planisiliqua*, has to be excluded from genus *Conringia* (tribe Conringieae), and it remains as a taxon with a new generic circumscription best integrated into tribe Isatideae.

The CES (Cremolobeae-Eudemeae-Schizopetaleae alliance) lineage with a largely South American distribution has been analyzed phylogenetically with limited DNA sequence information[50] including also Asteae, but members of tribes Scoliaxoneae and Kernereae were not included. All these tribes are closely related to Kernereae and to a lesser extent to Schizopetaleae. Among the remaining tribes (CES plus Asteae, Scoliaxoneae) plastome-derived data basically show a polytomy, and divergence time estimates of respective divergence times are not different. Therefore, tribal resolution and relationships as demonstrated earlier[50] remains questionable and unresolved, and generic assignment to tribes is sometimes not possible (see above).

Supplementary Note 3. BrassiBase as knowledge database and the morphological variation for family-wide genus delimitation

During the past 10 years, substantial progress has been made resolving systematics and phylogeny of a plant family that on the one hand serves as important model system[14], but on the other is known to be a notoriously difficult family in respect to taxonomy based on morphological diversity. Previous taxonomical concepts relying on morphology did not reflect evolutionary history properly[10], and because of parallel evolution in nearly any character used in circumscription of species and genera there is no comprehensive systematic-taxonomic backbone provided.

Biological, molecular and evolutionary knowledge about the mustard family is constantly increasing. However, due to the complex and overwhelmingly large biological diversity of the family, it is difficult to assess research results within a larger evolutionary framework. Many species have been proven to be suitable study objects but are rarely available. Biological material and resources, either collected directly in the wild or held in germplasm collections, have often been misidentified; and only very rarely has the material been further characterized and documented. There is also no comprehensive survey of morphological character and biological trait distribution among Brassicaceae lineages. In order to close these gaps, we made accessible to the scientific community the research data focusing on adaptive characters and their evolution in the Brassicaceae. In this context, we provide a comprehensive documentation of the taxonomy and systematics of the entire family. This includes a database with all relevant taxonomic, systematic and phylogenetic literature, a comprehensive data collection of characters, a DNA-based identification tool for genera and species, electronic interactive keys for the identification of genera, and information on chromosome numbers and genomes sizes. The system was first launched in 2012 (https://brassibase.cos.uni-heidelberg.de/)[18] with subsequent continuous improvement.

The current morphomatrix was built upon a concept to determine all Brassicaceae genera using diagnostic morphological characters[74]. The original concept was used to design an 'interactive' key, which was first implemented in BrassiBase with version 1.2 in 2017[14]. The herein presented morphomatrix takes advantage of this concept, because characters define any genus, and, thereby, Brassicaceae morphological diversity is covered comprehensively.

In contrast to a data matrix used to determine genera with an interactive key, the morphomatrix had to fulfill additional requirements to allow for subsequent data analyses: (i) no missing data is allowed, (ii) number of character states should be moderate, and (iii) characters should be independent from each other.

The work flow included an adjustment of characters and their states, initially designed for genera identification, to the purposes of character analysis in an evolutionary context: This work included, in particular, modification of certain characters (e.g. split of the initial character 'presence of plant thorns' into stem and

leaf thorns) and new grouping of character states (e.g. transformation of initial 14 states/types of trichomes into four more general types). As a result, the final morphomatrix comprised 37 characters represented by 111 character states in 351 genera. All character states are unordered and discrete. This corresponds to 38,961 unique character states covering morphological variation of Brassicaceae at the generic level. During the entire project phase all previous genus descriptions were checked, and in many cases original vouchers (visits in herbaria throughout Europe and Asia, e.g. ALTB, B, BM, E, G, GAT, H, HBG, HEID, JE, LE, LI, M, MW, OSBU, P, W, WU and literature on species descriptions was consulted. Most of the literature is integrated into BrassiBase version 1.3 (launched with this contribution), which currently provides ca. 3500 regularly updated genus-linked references[18,75], and a respective tool is now integrated with this contribution.

Furthermore, revision of the data was necessary in accordance with the numerous changes in generic concepts since 2012. This taxonomic work included updates for the newly described or restored genera (altogether 40) and those reduced to synonymy (5) since 2012 along with those affected by these taxonomic shifts. A number of further changes were simply caused by description or discovery of new species which did not alter taxonomic circumscription of relevant genera but impacted (sometimes severely as, for example, in *Dactylocardamum*) their morphological characteristics. The following genera in particular were updated in this contribution: *Boechera*, *Borodinia* and *Yosemitea*[22], *Friedrichkarlmeyeria*, *Ihsanalshehbazia* and *Microthlaspi*[28], *Noccaea* s. l.[76], *Caulanthus*[77], *Pseudocamelina*[78,79], *Bengt-jonsellia*[80], *Leiospora* and *Parrya*[81], *Anzhengxia*, *Braya*, *Metashangrilaia*, *Neotorularia* and *Rudolf-kamelinia*[82], *Malcolmia* sens. trad.[83], *Englerocharis*[84], *Weberbauera*[85], *Dactylocardamum*[86], *Brassica*[87], *Hilliella* and *Yinshania*[88,89], *Shehbazia*[90], *Eutrema* s.l. and *Pegaeophyton*[30], *Cardamine*[91], *Terraria*[92], *Zuloagocardamum*[93], *Alshehbazia* and *Onuris*[94], *Aimara*[70] and *Menonvillea*[70,95], *Xerodraba*[69], multiple genera of the tribe Alysseae including the new *Cuprella* and *Resetnikia*[96], *Atacama* and *Mathewsia*[65], *Berteroa*[97], *Orychophragmus* and *Sinalliaria*[98], *Quidproquo* and *Raphanus*[99]. In addition, major recent treatments covering numerous genera[100,101] were very useful for various updates in the morphomatrix. Particular efforts were made to find and improve, whenever possible, incongruence in the matrix caused by inconsistencies among descriptions, some terminological differences among different authors, occasional mistakes, etc. For this purpose, additional work with herbarium specimens from ALTB, B, G, GAT, H, HBG, HEID, JE, LE, LI, M, MW, OSBU, P, W, WU was conducted. An earlier and provisional species checklist for the Brassicaceae included 3709 species from 338 genera and 25 tribes[51]. Our new checklist incorporated changes that affected 860 accepted species names, more than 3500 synonyms, and resulted in 15,365 data entries in BrassiBase[14] and is accessible with version 1.3 accompanying this work.

The final morphomatrix represents a multistate character matrix on genus level, and therefore does not allow analysis within a given genus.

During the course of preparing the morphomatrix for disparity and phylogenetic signal analysis, we also optimized and reworked the original matrix for the interactive key. The matrix for the interactive key, which can be used to determine any genus of the Brassicaceae, is now also implemented in BrassiBase version 1.3 [https://brassibase.cos.uni-heidelberg.de/?action=intkey] with its new release accompanying this contribution. This data matrix includes 38 characters with 166 characters plus geographical distribution data. The ‘interactive key matrix’ and dataset is optimized for genus determination and not considered for any statistical analysis.

Supplementary Methods

Introduction into the screening of Brassicaceae morphological characters and its variation – the 'morphomatrix'

Although Brassicaceae are morphologically well-defined and easily distinguishable from any other angiosperm family by their general and little variation in flower architecture (almost always 4 sepals, 4 petals, 4+2 stamens, 2 fused carpels) and fruit characteristics (siliques or silicles, which additionally can be compressed in two different ways), infra-familiar grouping based on morphological characters frequently failed on higher taxonomic levels such as tribes and often even on a genus level. In the past, this often resulted in paraphyletic taxa.

As a consequence, there is no 'backbone set' of characters that can be selected *a priori* and studied across the entire family. Traditionally, characters in Brassicaceae have been elaborated on to describe, characterize and determine morphological variation on the species and genus level, but not with the aim to systematically compare genera or other higher order taxonomic units.

Furthermore, any taxonomic concept from the past, preceding molecular systematics[102–104], until the late 1990s, turned out to be highly artificial and very often did not define monophyletic groups correctly[13]. On the other hand, this also implies that previous concepts scoring different characters are not a priori biased by a phylogenetically constrained character selection.

The complex taxonomic history of Brassicaceae, with numerous changes and introductions of new taxonomic concepts over the past 100 years[74], mirrors this very well. Our own latest taxonomic species checklist for the entire family, which also takes significant phylogenetic evidence into account, resulted not only in a new total number of genera and species, but also introduced more than 15,000 corrections: An earlier species checklist for the Brassicaceae included 3,709 species from 338 genera and 25 tribes[51] and was the only available family-wide checklist until 2012. The updates introduced in the latest release of BrassiBase[18,75] from 2017[14] affected 860 accepted species names, 3,500 synonyms, and resulted in more than 15,365 data entries. The number of tribes increased from 25 to 51, which reflects firm recognition of monophyletic assemblies of genera. Although the number of genera (currently 351 are accepted in BrassiBase) seems to be constant over time, this steady state is due to 26 newly defined genera and the simultaneous reduction to synonymy of a similar number of genera. Finally, species number increased from 3,709 to 3,973, corresponding to a net increase of 4% of accepted species names[14].

In summary, characters and their states have been scored very carefully in previous approaches, in particular on the genus level. This knowledge has been accumulated by Ihsan A. Al-Shehbaz, who presented a first concept of an interactive morphological key to the genera of Brassicaceae (largely based on Appel & Al-Shehbaz[105]). This key comprised a comprehensive character set reflecting our

knowledge of variable characters among Brassicaceae species and genera. However, characters in this early key often used numerous character states (up to 17) or combined characters from stem, leaves, flowers and fruits. Furthermore, this key often failed to assign genera correctly to tribes, since phylogenetic analyses have substantially improved our understanding and concepts of monophyletic tribes since then.

Here we present as an additional result not only an improved and updated interactive key, which is now implemented and accessible through the latest release of BrassiBase (https://brassibase.cos.uni-heidelberg.de/), but we also developed a new morphological data matrix with reduced complexity that coded characters and their states in a way that is more applicable to study morphological characters/traits and their evolution.

We aimed to define approximately five character states, and whenever possible we split information content according to different plant organs. The matrix summarizes occurrence of character states on the genus level, and it does not take frequency of character states (species level) into account.

As data source we used any available genus description from literature. This literature is also provided with BrassiBase and can be found with the respective genera (Taxonomy tool, Literature descriptor; https://brassibase.cos.uni-heidelberg.de/?action=tax). In total the database includes more than 3,500 literature entries. Important to note is that thousands of vouchers had to be looked at to cross-validate (sometimes wrongly) earlier published information, and, finally, for various species only the original vouchers provided reliable information to score genus-specific morphological variation. The entire process presented here is the result of more than 10 years of intensive work of a team of the leading experts in Brassicaceae systematics and taxonomy, finally resulting in a data-matrix without any missing data at the genus level comprising 351 genera and 37 characters (totaling 111 character states). All character states are discrete and unordered. The frequency of character states is shown as pie charts for each character in Supplementary Figure 15. In the following the characters and their states are described in more detail and are grouped arbitrarily into six categories (A: General characters; B: Indumentum; C: Stem characters; D: Leaf characters; E: Flower and inflorescence characters; F: Fruit and seed/embryo characters). This coding, of course, also considers previous descriptions of character states in Brassicaceae[8,106]

In the following the characters and their states are listed and defined accordingly:

A. GENERAL CHARACTERS

General characters describe features of a genus that do not explicitly refer to a plant organ or a specialized structure.

(1) Duration:

Classical botanical literature and earlier descriptions of Brassicaceae often did not recognize monocarpic versus polycarpic species, but defined life span (duration). The

focus here lies on duration because information on polycarpic vs. monocarpic behavior is not available. However, annuality and bienniality are highly correlated to monocarpic flowering in Brassicaceae. Duration can therefore be treated as a discrete character rather than a continuous one, with perennial being anything above two years.

1. Annual or biennial
2. Perennial

(2) Habit

Habit largely refers to life form. Shrubs and subshrubs are defined as woody, perennial phanerophytes. This circumscription does not define a minimum plant height of subshrubs and shrubs. It has also been noted that numerous herbs are woody as well, hence, character state coding cannot be used to score woodiness.

1. Herbs (anything that is no shrub or liana)
2. Shrubs or subshrubs (woodiness, perennials, phanerophytes)
3. Lianas (Not self-supporting)

B. INDUMENTUM

Indumentum is often a key character in Brassicaceae systematics and taxonomy. Accordingly, there is a large diversity of character states being described in taxonomic and morphological literature. Hence, here we present a synoptic perspective, that does not have the ‘full diagnostic capacity’ in morphological keys.

(3.) Multicellular glandular hairs

Binary character. It refers to the presence or absence of glandular hairs on any structure of the plant. This character does not refer to pores with subepidermal cells acting as secretory tissue (e.g. carpels of some *Cardamine* species) or any secretory tissue (e.g. nectaries).

1. Present
2. Absent

(4.) Hairs [types of trichomes]

The character was ordered by increasing macroscopic complexity: 1,2,3,4.

The additive complexity is given by the states ‘branched’ and ‘stalked’, which finally result in complex three-dimensional structures. Some of the original complexity of character states is given for orientation, and full details can be found with the interactive key at BrassiBase.

1. Absent
2. Simple (simple + hooked at apex)
3. Branched and sessile (malpighiaceous + stellate and sessile + 3–6-rayed stellate and sessile + 6- to 12-rayed stellate + 12- to many-rayed stellate + lepidote scales + cruciform)

4. Branched and stalked (forked + dendritic + stellate and stalked + barbellate)

C. STEM CHARACTERS

(5) Stem thorns

Thorn-like structures anywhere on the plant but the leaves or petals. Spike-like structures (e.g. such as in *Rosa*) are not found in Brassicaceae.

1. Present
2. Absent

D. LEAF CHARACTERS

In Brassicaceae, leaf characters are often key to genera and species since flower characters do not have the same discriminatory power, and fruits are often not available. However, the total number of characters used in traditional circumscriptions of leaves is relatively low.

(6.) Basal leaves rosette forming

This character is scored during later phases of plant development. However, exact developmental stages are not defined in taxonomical literature.

1. Rosulate
2. Not rosulate including absent

(7.) Division and margins of basal leaves

Basal leaves are those developed at the stem base, either forming a rosette or as few single leaves. Character state 1 applies if basal leaves are transient and are not present anymore even in early stages of plant stem development. Character states 2 to 5 are arranged with increasing complexity and further explanations (subtypes) are given.

1. Not applicable
2. Entire to sinuate (entire or repand + dentate, denticulate, serrate, incised, crenate, sinuate)
3. Lobed (pinnately lobed + palmately lobed)
4. 1–3-pinnatisect
5. Compound (trifoliolate + pinnately compound + palmately compound)

(8.) Occurrence of stem leaves

Most often Brassicaceae develop stem leaves (usually more than one).

1. Present
2. Absent

(9.) Appearance of stem leaves

Character state 1 applies if stem leaves are absent.

1. Not applicable
2. Petiolate
3. Sessile but not auriculate or amplexicaul
4. Auriculate, amplexicaul, or sagittate

(10.) Division and margins of stem leaves

Divisions and margins of stem leaves can show patterns of different complexity. In the simplest case the margins are entire while in the most complex case leaves are compound. Leaf morphology exhibits high variation. Leaves are predominantly simple in the Brassicaceae (undivided to strongly divided) or rarely compound. Character states are arranged with increasing complexity.

1. Not applicable
2. Entire to sinuate (entire or repand + dentate, denticulate, serrate, incised, crenate, sinuate)
3. Lobed (pinnately lobed + palmately lobed)
4. 1–3-pinnatisect
5. Compound (trifoliolate + pinnately compound + palmately compound)

(11) Leaf thorns

Generally, leaf thorns are rare in Brassicaceae, but it was necessary to separate the few cases from ‘stem thorns’ (character 5) to separate between plant organs.

1. Present
2. Absent

E. FLOWERS AND INFLORESCENCES

In Brassicaceae, flower morphology shows only limited variation in terms of the number of organs and the general flower structure (usually 4 sepals, 4 petals, 4+2 stamen, 2 fused carpels). In most cases flowers are arranged in raceme-like inflorescences. Less often the flowers are solitary.

(12.) Inflorescences and flower arrangement

Bracts are stem leaves in whose axil a flower evolves. In Brassicaceae inflorescences can be bracteate or ebracteate. However, also solitary flowers can be found.

1. Racemes that are bracteate (throughout or at least in lower half)
2. Racemes that are ebracteate (rarely lowermost 1 or 2 flowers bracteate)

3. Solitary flowers on pedicels originating from basal rosette

(13.) Petal presence and evolvement

Brassicaceae usually have four petals, which in some cases can be absent. In rare cases petal number can deviate (higher or lower), which can be an instable or stable character depending on the species. This character is sorted by degree of evolvement.

1. Absent
2. Distinctly longer than sepals
3. Subequalling sepals
4. Reduced (smaller than sepals)

(14.) Petal color

In Brassicaceae, most often petals are white, yellow or pink/purple. Some species can vary in petal color, have bicolored petals or change the intensity of their petal color during development. We scored the prevalent color of the blade in our study. Some petal colors were scored in combination. Yellow and orange as well as pink and purple occur in various shades of intermediates, and were therefore combined. Green and brown were combined as in some cases the two colors cannot be distinguished.

1. White
2. Yellow + orange
3. Pink + purple
4. Green + brown
5. Blue

(15.) Petal shape

Petals in Brassicaceae can have various shapes, and the definition of shape is often difficult and varies from author to author. Therefore, we grouped the petal shape according to relative petal width (relative to petal length). The category ‘intermediate’ combines states between clearly wide and clearly narrow.

1. ‘Wide’ (obovate + oblong to elliptic + orbicular + obcordate)
2. ‘Intermediate’ (spathulate to oblanceolate)
3. ‘Narrow’ (linear + filiform)

(16.) Petal apex

Petals in Brassicaceae are usually undivided, and most commonly the apex of the petal is entire or emarginate. In some cases, the apex can be bifid. The character was sorted according to increasing complexity.

1. Obtuse, rounded, or truncate
2. Retuse or emarginate
3. Deeply bifid

(17.) Petal margin

Petals are usually undivided in the Brassicaceae. In the vast majority of cases the petal margin is entire. Character states are sorted according to increasing complexity.

1. Entire
2. Dentate
3. Pinnatifid
4. Fimbriate

(18.) Sepal orientation

Sepals can be variously oriented in the Brassicaceae. Orientation is scored when the flower is fully opened. Character states are sorted from completely closed to completely open calyx.

1. Erect
2. Ascending
3. Spreading
4. Reflexed

(19.) Sepal union

Sepals can either be separate/free or joined (united) at their margins (grown together). In Brassicaceae, sepals are usually free but occasionally the sepals are fused (united). Cases in which sepals are hooked by trichomes were scored as ‘free’.

1. Free
2. United

(20.) Stamen number

Usually, Brassicaceae have six stamens arranged in two whorls, where the inner consists of four longer and the outer of two shorter stamens. In rare cases stamen number deviates in either direction. These deviations may be stable or unstable within species.

1. Six
2. Four
3. Two
4. More than six

(21.) Lower part of filaments and petal claws

Usually, filaments and petals do not have any additional structures. However, in a number of species additional structures can be found, such as wings, teeth or appendages of stamens and, rarely, petal claws. While teeth and appendages are

always clearly present, wings can be developed to a different degree. Wings were only scored as such when they were clearly observable. In addition, some species are characterized by the presence of pubescent or papillate petal claws or filaments.

1. Both without anything
2. Filaments with a wing, tooth, or appendage
3. Petal claw and/or filament with hairs or papillae

(22.) Filaments of median stamens

In Brassicaceae-morphology the term 'median stamen' refers to stamens in the inner whorl, while the outer stamens are referred to as 'lateral stamens'. Commonly, all stamens are free but sometimes the median stamens are united in pairs to a different degree, sometimes only at the base. Stamens were only scored as united when they were at least connected along the lower third.

1. Free
2. United at least along lower third

(23.) Flower symmetry

In Brassicaceae, in most cases flowers are actinomorphic (radially symmetrical), meaning that all petals have the same size and are evenly arranged in their respective whorl. Stamens were not considered in the definition of flower symmetry. Rarely, zygomorphic (bilaterally symmetrical) flowers occur in the family due to petals of unequal size or their specific arrangements in the whorl.

1. Actinomorphic
2. Zygomorphic

F. FRUITS [AND SEEDS]

Usually in Brassicaceae the fruit is a 2-valved capsule, which is referred to as a silique or silicle depending on the ratio between length and width (<3:1 silicle; >3:1 silique). Fruit shape in the Brassicaceae is highly diverse in respect to shape, size and structure and it is one of the most important characters for species determination.

(24.) Fruit type

According to the ratio between length and width, fruits in the Brassicaceae are traditionally divided into two major groups. Siliques are defined as being at least 3 (to 4) times longer than wide, while silicles are only up to 3 times longer than wide or generally wider than long. Absolute size does not matter here, and in total a silicle can be bigger than a silique. The delimitation of 'silique' and 'silicle' is artificially drawn and mainly serves for the purposes of taxonomy. Here, it is used as a binary character, although in a strict sense fruit length to width ratio would be a continuous character.

1. Silique
2. Silicle

(25.) Fruit flattening

Fruits in Brassicaceae can be flattened or not. In the first case, fruits are either flattened parallel (latiseptate) or perpendicular (angustiseptate) to the septum. In the second case, fruits are either round (terete) or quadrangular in cross section.

1. Angustiseptate
2. Latiseptate
3. Terete
4. Quadrangular

(26.) Fruit or segment wall

Depending on the texture of the wall, fruits can be subdivided into three groups. This character is actually continuous but still reflects a particular aspect of fruit diversity of Brassicaceae.

1. Woody or corky and thick
2. Thin or thick leathery
3. Papery

(27.) Fruit dehiscence

Most fruits in Brassicaceae are dehiscent, although indehiscent fruits also occur in a smaller number of genera. Indehiscent fruits also include rare thick-walled, nut-like fruits. A large number of indehiscent fruits break up into single or few-seeded segments. Fruits which exhibit dehiscent and indehiscent segments were scored as indehiscent. Heterocarpic taxa were scored as both dehiscent and indehiscent as they exhibit both character states.

1. Dehiscent
2. Indehiscent
3. Fruit breaking up into closed, 1- or few-seeded segments

(28.) Gynophore in fruit

The gynophore is an additional segment in the fruit located between the pedicel and the ovary. Usually, gynophores in Brassicaceae are short or absent, but they can also be very pronounced in some cases.

1. At least 1.5 mm long, usually much longer
2. Absent or rarely to 1 mm long

(29.) Septum in mature fruit

Usually, fruits in the Brassicaceae are divided into two halves by a septum. In most cases the septum is complete; however, sometimes it is perforated or reduced to a rim or it can be completely absent.

1. Lacking or reduced to a narrow rim

2. Complete or rarely with a hole

(30.) Stigma lobing in fruit

In Brassicaceae, three main stigma types can be recognized: entire stigma and two kinds of lobed stigma (with diverged and connivent lobes). All three types are about equally common across the family.

1. Entire

2. Lobes not decurrent

3. Lobes decurrent and connivent

(31.) Presence of fruit appendages

In Brassicaceae, fruits usually do not have any additional structures associated to their dispersal. Sometimes additional structures such as wings, horns, spines and crests are present, with wings being the most common type. In some taxa, glochidiate (hooked) trichomes are present, which are also associated to dispersal. However, these structures are not appendages in the strict sense and are therefore not considered in the character state ‘present’. Furthermore, spines, which are derived from epidermal tissue, were not scored as present (only one case known).

1. Present

2. Absent

(32.) Number of ovules/seeds per ovary/fruit

The number of ovules/seeds per ovary/fruit is highly diverse in the Brassicaceae and ranges from one or two to more than 100. If the range covered more than one character state, all respective character states covered were scored.

1. One or two

2. Four to ten

3. (Eleven) 12 to 20

4. More than 20

(33.) Seed arrangement per locule

In Brassicaceae, seeds may be arranged in the locule in different ways. Seeds arranged in one line are referred to as uniseriate, while seeds arranged in two parallel lines are referred to as biseriate. Seeds falling in neither of the two categories are called aseriate and are typically found in one- or few-seeded fruits. All three types are common.

1. Uniseriate

2. Biseriate

3. Aseriate

(34.) Seed wing

In Brassicaceae, the testa of the seeds can form wings (flat, thin margins). Wings can be narrow or wide and can be developed completely around the seed, or may be restricted to short portions. Winged seeds are slightly less common than wingless seeds.

1. Present
2. Absent

(35.) Cotyledonary position

Cotyledonary position was traditionally an important character in systematics of Brassicaceae because cotyledons can be variously arranged relative to the radicle in seeds. The radicle may be positioned along the margins of both cotyledons (accumbent), on the back of one cotyledon (incumbent), or both cotyledons may fold around the radicle (conduplicate). Accumbent, incumbent and conduplicate are the most common type of arrangement. The various types of other arrangements (character state 4) all refer to rolled up, folded or coiled arrangements and rarely occur in the family. In one case the radicle is reduced. Here, the embryo is defined as straight and cotyledonary position cannot be defined (straight = not applicable here).

1. Accumbent
2. Incumbent
3. Conduplicate
4. Other types
5. Straight

(36.) Seed mucilage

In Brassicaceae, numerous species produce myxospermous seeds (forming mucilage around the seed-coat when wet). The degree of capacity to form mucilage differs from taxon to taxon. Taxa producing only very small amounts of mucilage are also scored as ‘present’.

1. Present
2. Absent

(37.) Fruit orientation

In Brassicaceae, fruits can be variously oriented in respect to the stem. They can be erect/ascending (upright), spread (pointing to the sides), or reflexed (pointing downwards). All orientations are commonly found and usually the orientation is species specific. Fruit orientation is determined when fruits are ripe.

1. Erect to spreading
2. Reflexed

Phylogenetic tree of exonic nrDNA cistron (18S-5.8S-26S)

All 194 accessions for which we generated raw reads were used for the reconstruction of a phylogenetic tree based on the exonic part of the nuclear ribosomal (nr)DNA cistron. Internal and external spacer regions were not included, as they vary too much among taxa across the family and could not be reliably aligned. Reads were processed using HybPhyloMaker[107]. Settings were as follows: Adapter sequences and low quality reads were removed with Trimmomatic 0.32[108]. In case of quality < Q20 of read ends, these bases were discarded. The remaining part of the read was trimmed, if average quality in a 5 bp window was < Q20, and removed, if read length fell below 36 bp after trimming. Duplicate reads were removed with FastUniq 1.1[109]. Quality-trimmed, filtered reads were then mapped to a 'pseudoreference' consisting of the 18S, 5.8S and 26S sequences of *Brassica rapa* subsp. *pekinensis* (ENA/GenBank accession KM538956; [https://www.ncbi.nlm.nih.gov/nuccore/KM538956]) that were separated by a stretch of 400 Ns in between each sequence using Bowtie 2 2.2.4[110] with the '-very-sensitive-local' flag. The number of mapped reads to the 'pseudoreference' was 158,544 on average (minimum 3,144; maximum 2,461,553), which corresponds to an average of 1% of the trimmed, filtered reads. Consensus sequences per taxon were constructed with a minimum of 10x read depth and at least 51% majority consensus for base calling using Kindel 0.1.4[111]. The 51% majority consensus results in the reconstruction of the most abundant sequence. Consensus sequences were matched to the nrDNA cistron sequences using BLAT 32×1[112] with 90% similarity to produce PSLX files. The script 'assembled_exons_to_fasta.py'[113] was used to construct matrices for multiple sequence alignments. Sequences were aligned using default settings with MAFFT 7.029[114] and then joined in a single gene alignment using AMAS 0.98[115]. This alignment, partitioned into 18S (1,810 bp), 5.8S (164 bp) and 26S (3,388 bp), contained 0.04% missing data. ModelTest-NG 0.1.6[116] was then used to select the best-fit substitution model, which was selected based on the Bayesian information criterion (BIC) value and the (corrected) Akaike information criterion [AIC(c)] values. Ten substitution models were tested, and the best-fit models were GTR+I+G4 for 18S, TVM+I+G4 for 5.8S, and GTR+I+G4 for 26S. A phylogenetic tree based on the concatenated, partitioned alignment (5,362 bp total length; number of variable/parsimony informative sites for 18S: 180/116, 5.8S: 24/13, and 26S: 696/487) was reconstructed using maximum likelihood in RAxML-NG 0.8[117]. Bootstopping[118] was used with a maximum of 1,000 bootstrap replicates; bootstrapping converged after 750 replicates. Felsenstein's bootstrap was used, as we think that the total number of taxa with < 200 does not justify using the Transfer Bootstrap Expectation support. To compare tree topologies between our nrDNA and plastome trees and the nuclear phylogeny from Nikolov *et al*., we pruned the trees to contain only a comparable set of taxa and inserted polytomies for support values < 95% using phangorn 2.5.5[119] in R 3.5.2. The resulting trees were then compared using tanglegrams in dendroscope 3.7.2[120].

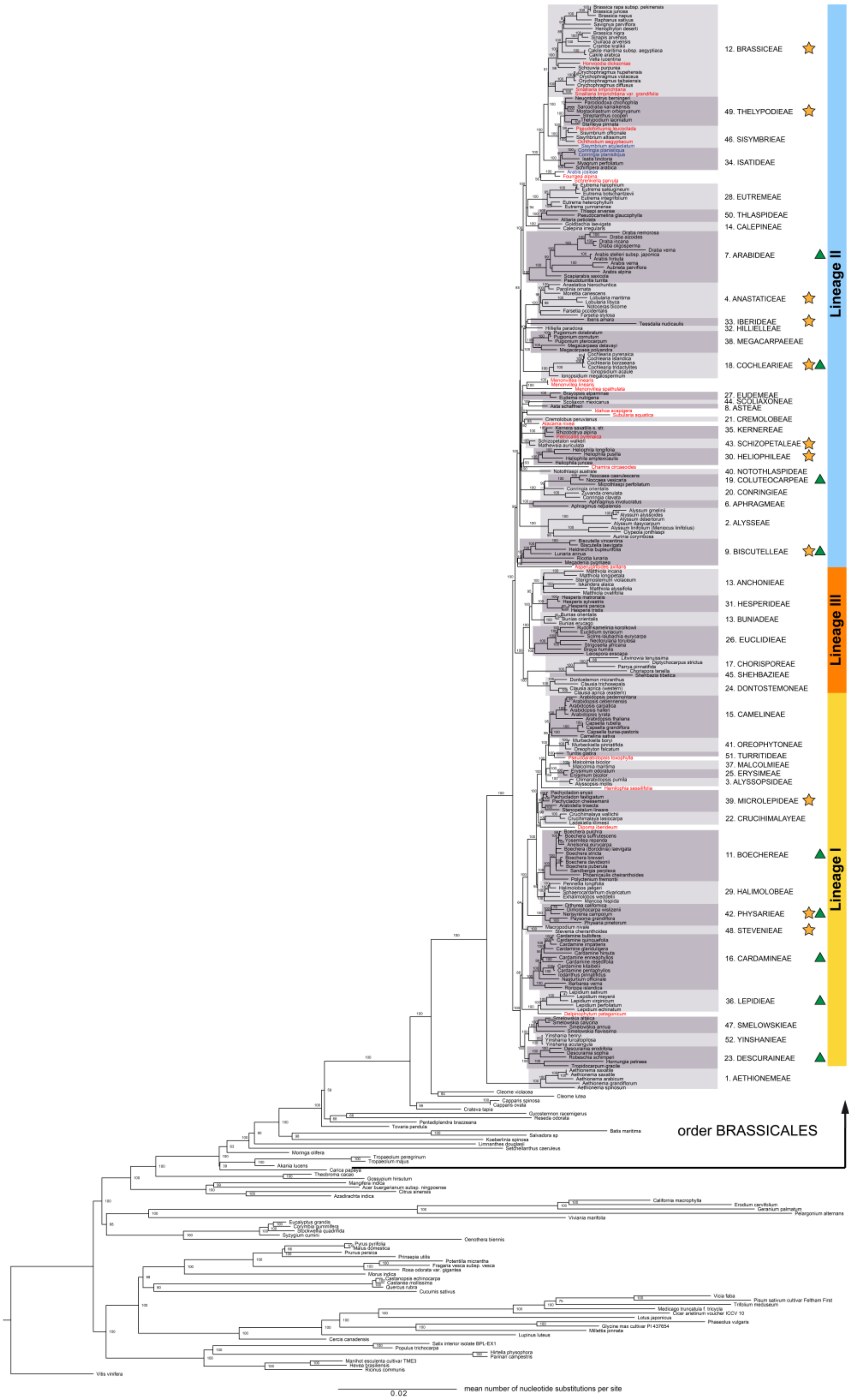

12. BRASSICEAE
49. THELYPODIEAE
46. SISYMBRIEAE
34. ISATIDEAE
28. EUTREMEAE
50. THLASPIDEAE
14. CALEPINEAE
7. ARABIDEAE
4. ANASTATICEAE
33. IBERIDEAE
32. HILLIELLEAE
38. MEGACARPAEEAE
18. COCHLEARIEAE
27. EUDEMEAE
44. SCOLIAXONEAE
8. ASTEAE
21. CREMOLOBEAE
35. KERNEREAE
43. SCHIZOPETALEAE
30. HELIOPHILEAE
40. NOTOTHLASPIDEAE
19. COLUTEOCARPEAE
20. CONRINGIEAE
6. APHRAGMEAE
2. ALYSSEAE
9. BISCUTELLEAE
Lineage II
13. ANCHONIEAE
31. HESPERIDEAE
13. BUNIADEAE
26. EUCLIDIEAE
17. CHORISPOREAE
45. SHEHBAZIEAE
24. DONTOSTEMONEAE
Lineage III
15. CAMELINEAE
41. OREOPHYTONEAE
51. TURRITIDEAE
37. MALCOLMIEAE
25. ERYSIMEAE
3. ALYSSOPSIDEAE
39. MICROLEPIDEAE
22. CRUCIHIMALAYEAE
11. BOECHEREAE
29. HALIMOLOBEAE
42. PHYSARIEAE
48. STEVENIEAE
16. CARDAMINEAE
36. LEPIDIEAE
47. SMELOWSKIEAE
52. YINSHANIEAE
23. DESCURAINEAE
1. AETHIONEMEAE
Lineage I
order BRASSICALES
0.02
mean number of nucleotide substitutions per site

Supplementary Figure 1. Maximum likelihood phylogeny of Brassicaceae and relatives. The coding sequences of 60 plastid genes from 250 Brassicaceae samples covering all tribes and outgroup species from additional 15 families of the order Brassicales as well as 18 families from the Rosidae were used for phylogenetic reconstruction in RAxML. Bootstrap support from 1000 rapid bootstrap replicates is given at the nodes. For Brassicaceae, assignment to the three lineages is given, and mesopolyploidization events[63,68] as well as significant rate shifts[71] are indicated with yellow stars and green triangles, respectively. Newly assigned, yet unassigned and remote genera (see Supplementary Note 2) are shown in red, and taxa in need of a new genus name (because the genus is polyphyletic) are shown in blue. Source data are provided as a Source Data file.

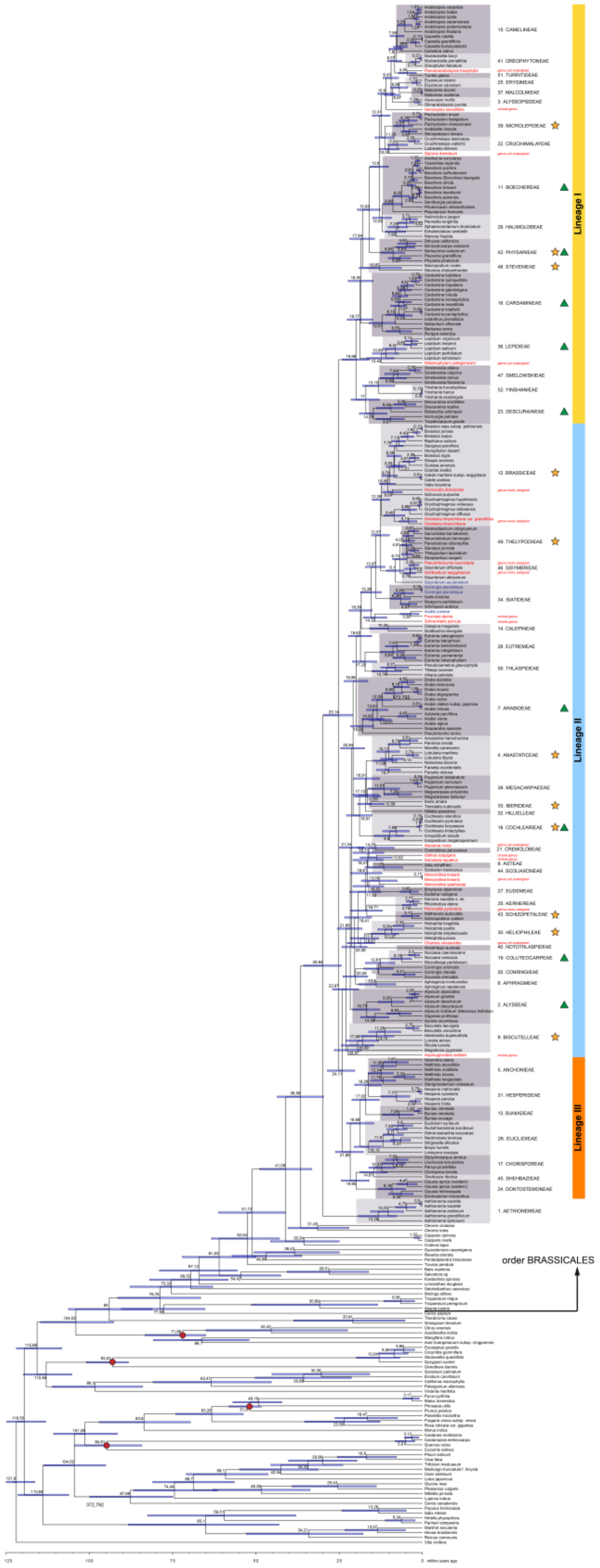

Supplementary Figure 2. Divergence time estimation of Brassicaceae and relatives. Divergence time was estimated using BEAST[121] following Hohmann *et al.*[2] and using fossil calibration at four nodes, indicated with red circles, within the Rosidae. Node ages (median node heights) and their 95% HPD intervals (blue bars) from 28 combined BEAST runs totaling 21,038 sampled trees are given. For Brassicaceae, assignment to the three lineages[9] are given, and mesopolyploidization events[63,68] as well as significant rate shifts[71] are indicated with yellow stars and green triangles, respectively. Newly assigned, yet unassigned and remote genera (see Supplementary Note 2) are shown in red, and taxa in need of a new genus name (because the genus is polyphyletic) are shown in blue. Source data are provided as a Source Data file.

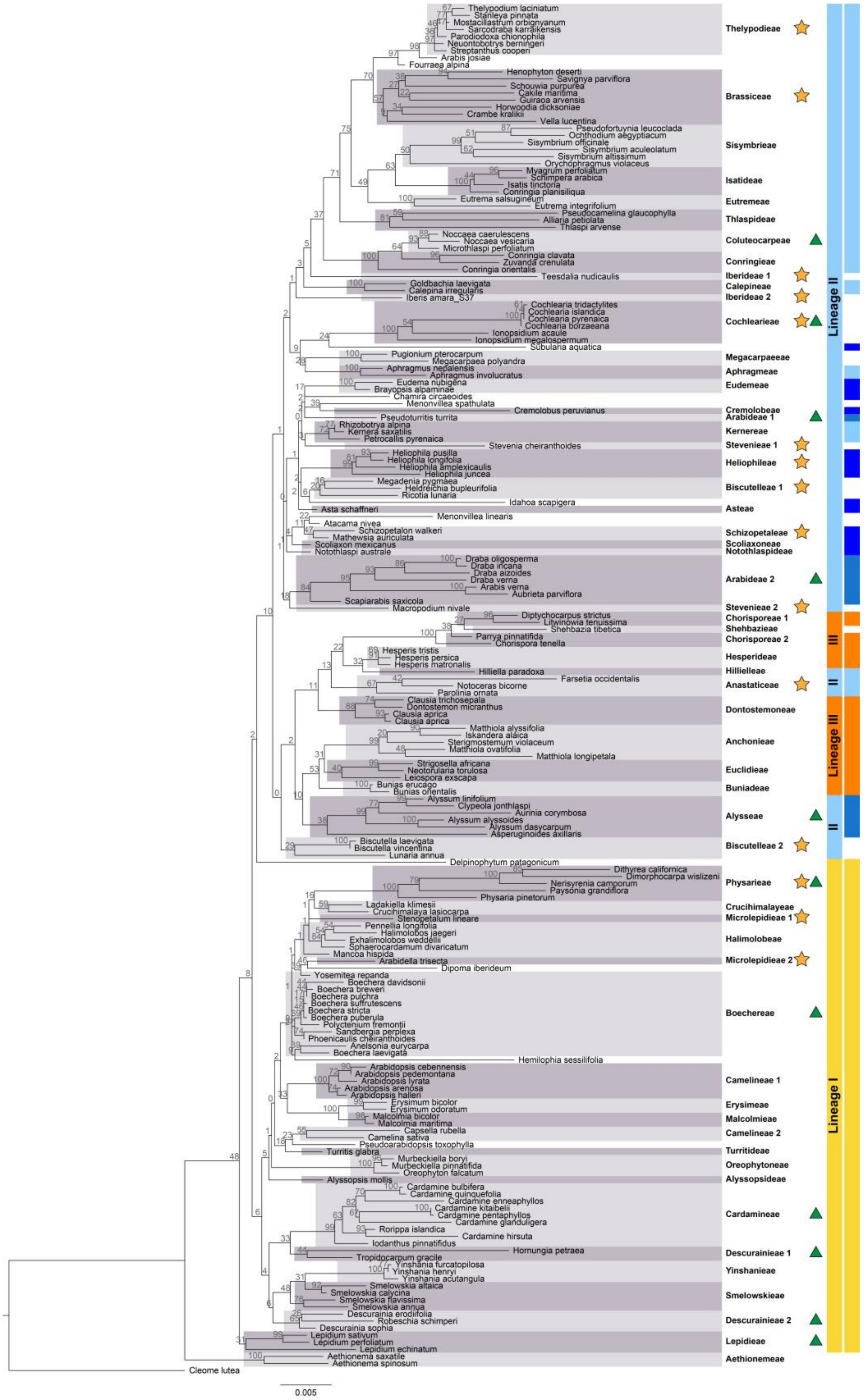

Supplementary Figure 3. Maximum likelihood phylogeny of Brassicaceae based on nuclear data. The nuclear encoded rDNA cistron (18S-5.8S-26S) was assembled from genome skimming data for all newly generated sequencing data. Rooting was performed at the branch of *Cleome lutea* (Cleomaceae). Bootstrap support is given at the nodes. Assignment to the three lineages[9] are given, and lineage assignment following the nuclear phylogeny presented by Nikolov *et al*.[5], with lineage II split into three lineages, is also indicated on the right. Mesopolyploidization events[63,68] as well as significant rate shifts[71] are indicated with yellow stars and green triangles, respectively. Source data are provided as a Source Data file.

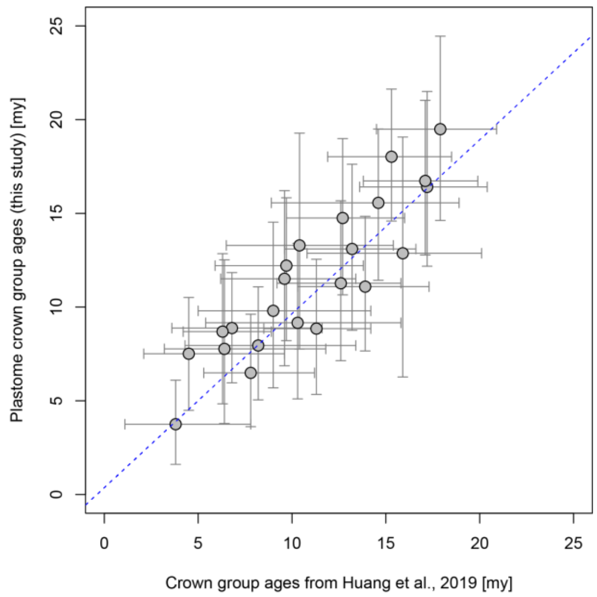

Supplementary Figure 4. Crown group ages. Crown group divergence time estimates for 24 tribes were compared to crown group ages from the literature[71]. Both time estimates were highly correlated (Spearman's rank correlation coefficient 0.89, *P*-value = 0.000003023). Linear regression is shown with the blue dashed line, adjusted R-squared was 0.7874. Grey circles represent median age estimates, bars represent upper and lower 95% HPD intervals. Source data are provided as a Source Data file.

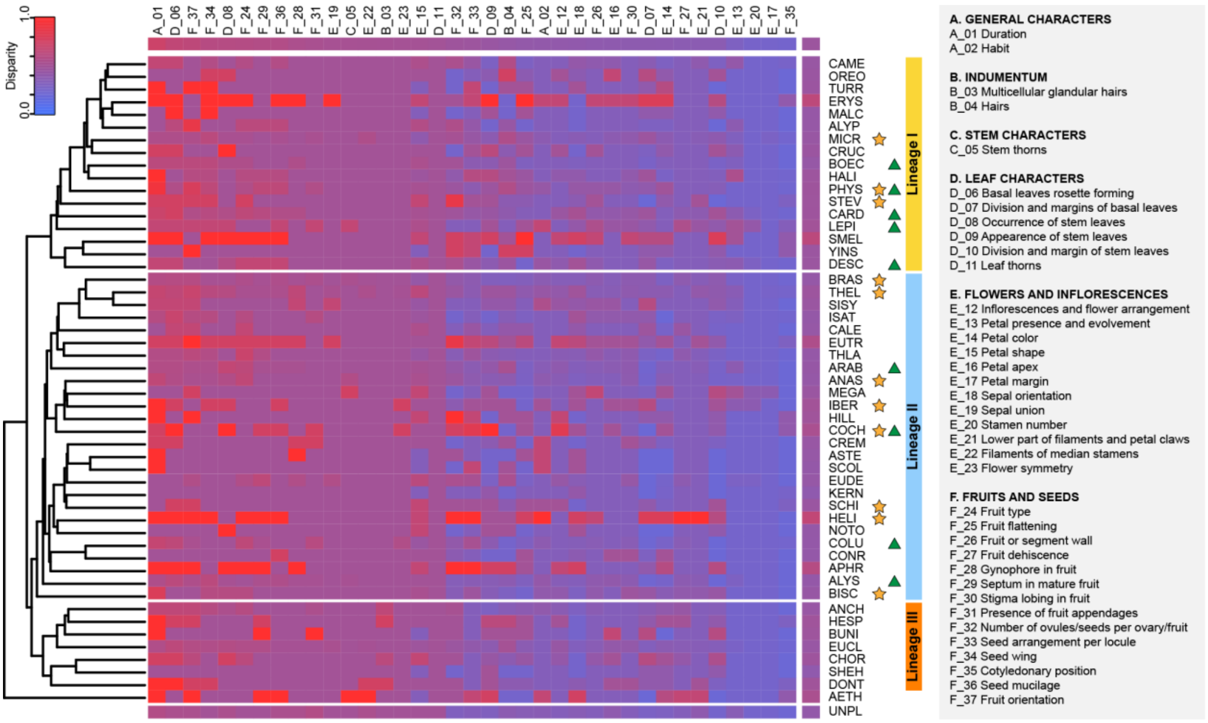

Supplementary Figure 5. Disparity heat map (disparity from genera). Disparity values for each character and tribe were calculated as mean values from genus level disparity. Tribes are sorted by phylogeny (following Fig. 1), and characters are sorted by disparity, with the highest mean disparity values on the left. Assignment to the three lineages[9] is given, and mesopolyploidization events[63,68] as well as significant rate shifts[71] are indicated with yellow stars and green triangles, respectively. The 37 characters in six categories (A-F) are given on the right. Disparity from genera was highly correlated with disparity calculated from tribal data (Spearman's rank correlation coefficient 0.691, *P*-value < 2.2×10^{-16}). Source data are provided as a Source Data file.

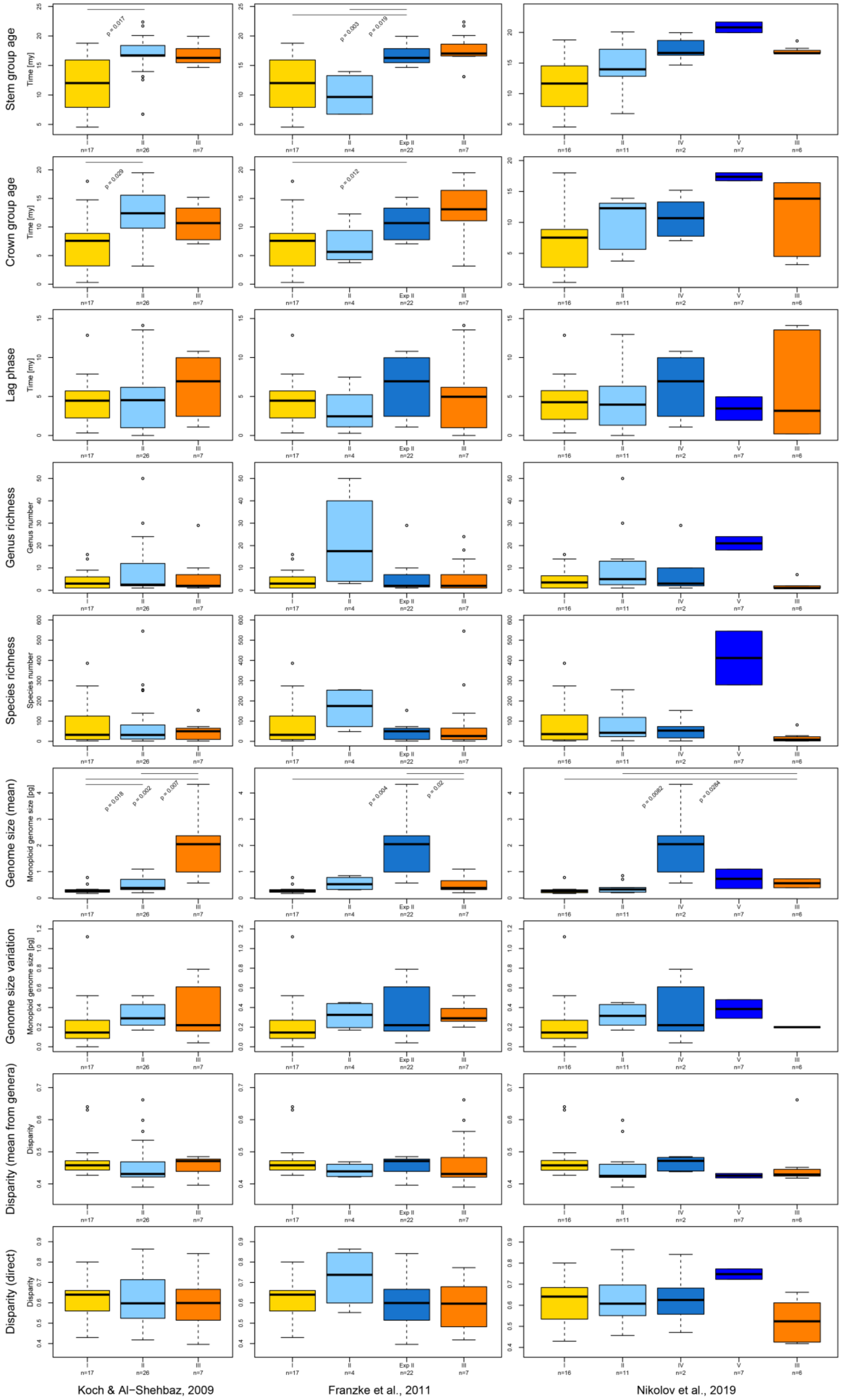
Stem group age
Crown group age
Lag phase
Genus richness
Species richness
Genome size (mean)
Genome size variation
Disparity (mean from genera)
Disparity (direct)
Time [my]
Genus number
Species number
Monoploid genome size [pg]
Disparity
Koch & Al−Shehbaz, 2009
Franzke et al., 2011
Nikolov et al., 2019

Supplementary Figure 6. Boxplot for comparison of Brassicaceae lineages. Lineage assignment following Koch & Al-Shehbaz [9], Franzke *et al*.[10] and Nikolov *et al*.[5]. Tribal values from Supplementary Table 2 excluding basal tribe Aethionemeae. When splitting Brassicaceae into three lineages (left panel), only mean stem group age, mean crown group age and mean genome size show a significant difference between lineages. Splitting Brassicaceae into four lineages (center panel), the same significant differences were found as in the analysis with three lineages, namely mean stem group age, mean crown group age and mean genome size. When splitting lineages further into five lineages (right panel), mean stem group age, mean crown group age, number of genera, number of species, mean genome size and disparity (from genera) showed a significant difference between lineages. However, because of the small sample sizes caused by splitting lineage II into three groups, with lineage IV consisting only of 2 tribes, most pairwise tests were not significant, with the exception of mean genome size, where we detected significant differences between lineage III and lineages I and II. In the boxplots, center line represents median; upper and lower quartiles are indicated by box limits; whiskers represent 1.5x interquartile range and points are outliers. Full test statistics of Kruskal-Wallis tests to detect differences between lineages are given in Supplementary Table 4. Pairwise significant differences were evaluated using Wilcoxon rank sum tests. *P*-values from two-sided tests with Bonferroni correction are given for pairwise comparisons above the boxplots. Full test statistics for Wilcoxon rank sum tests are given in Supplementary Tables 5-7. Source data are provided as a Source Data file.

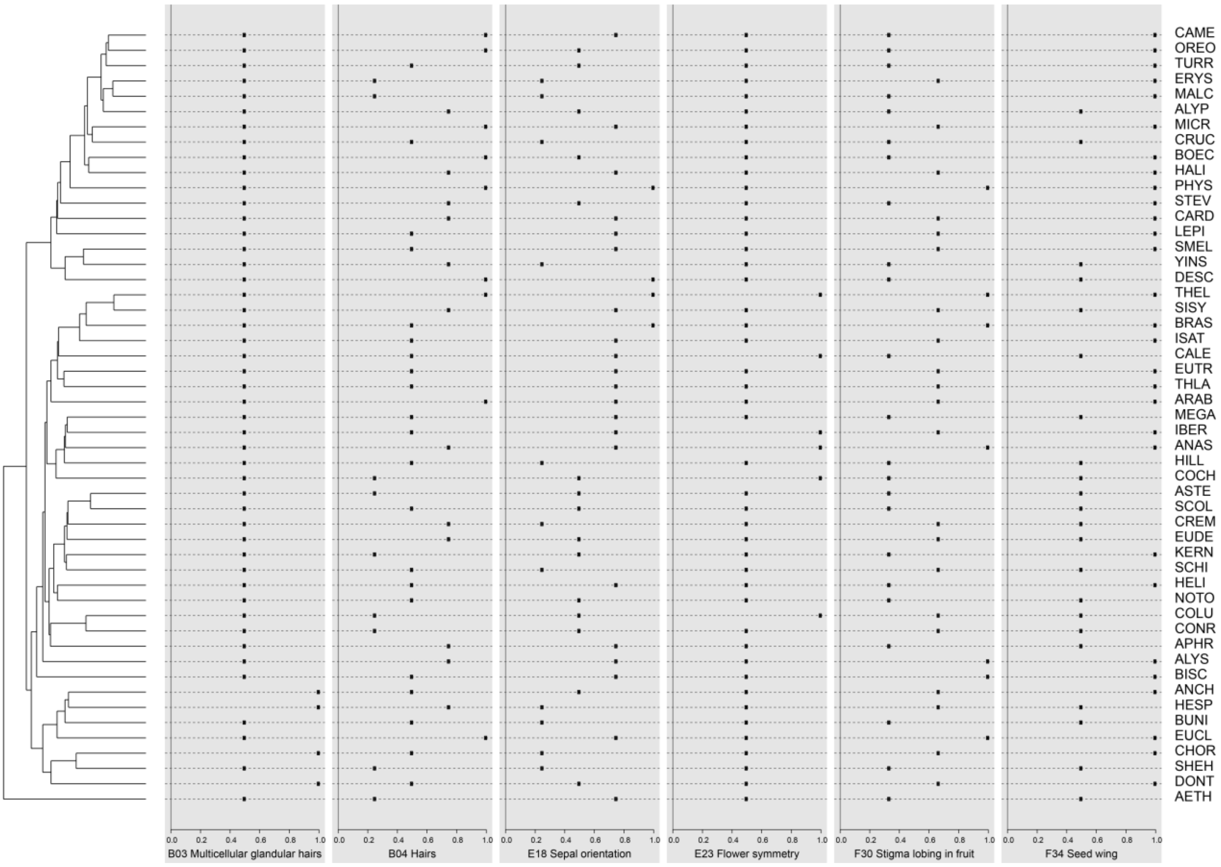

Supplementary Figure 7. Phylogenetic dotplot for disparity. Only the six characters with significant phylogenetic signal ('multicellular glandular hairs', 'hairs', 'sepal orientation', 'flower symmetry', 'stigma lobing in fruit', 'seed wing', see Supplementary Table 3) in their tribal disparity (direct) are shown. Source data are provided as a Source Data file.

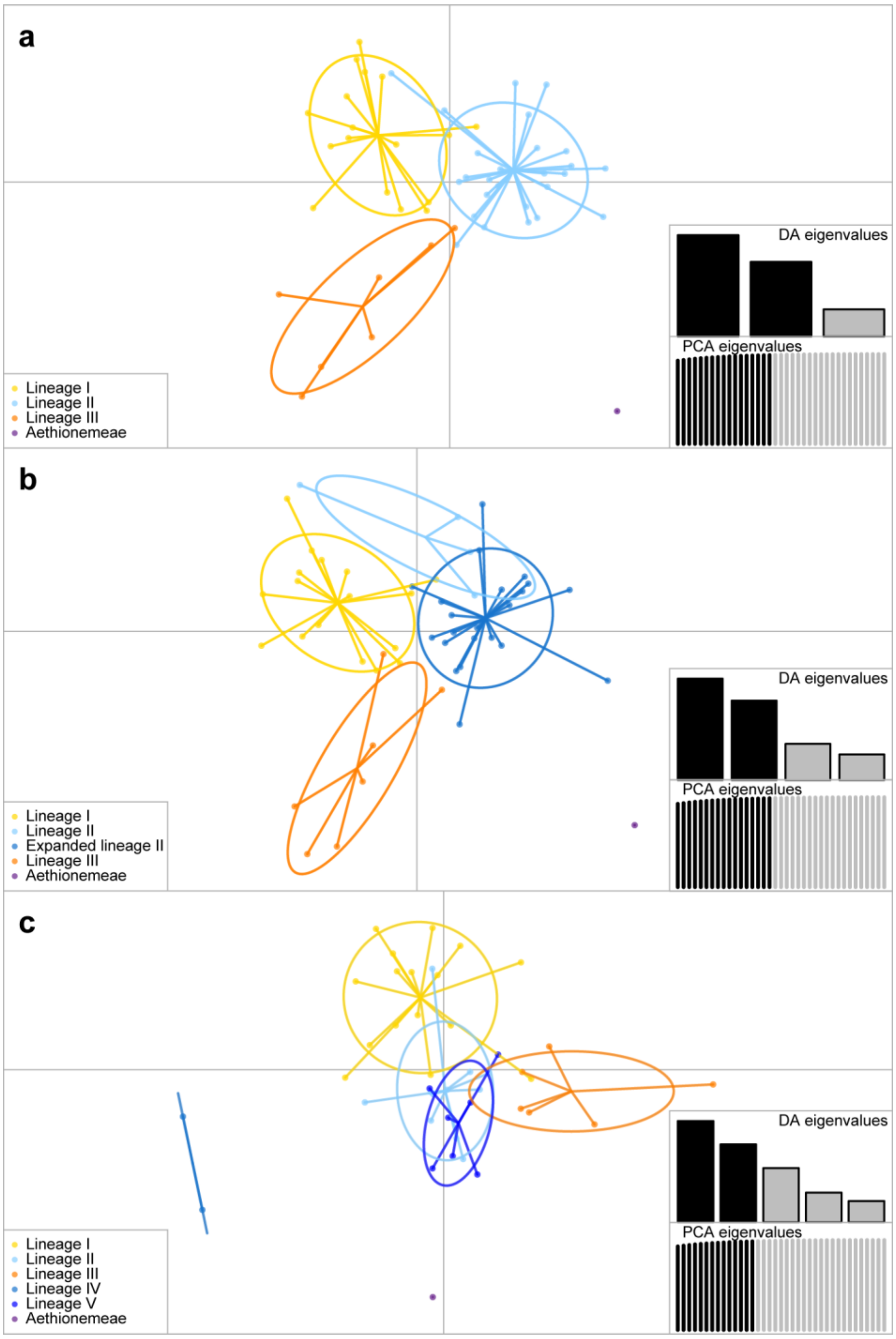

Supplementary Figure 8. DAPC scatterplots for different Brassicaceae lineage assignments. DAPC was conducted on tribal level disparity (direct) including basal tribe Aethionemeae in addition to the three (a) to five lineages. Splitting lineage II into (b) two lineages following Franzke *et al*.[10] or (c) three lineages following Nikolov *et al*.[5] had little effect on lineages I and III, which continued to be well separated. Furthermore, extended lineage II and lineage II (b) as well as lineage II and lineage V (c) largely overlapped; however, lineage IV (c) was separated from the rest. Note that for (c), the number of tribes was lower (n = 42) because the respective study[5] did not assign all tribes to a lineage. Source data are provided as a Source Data file.

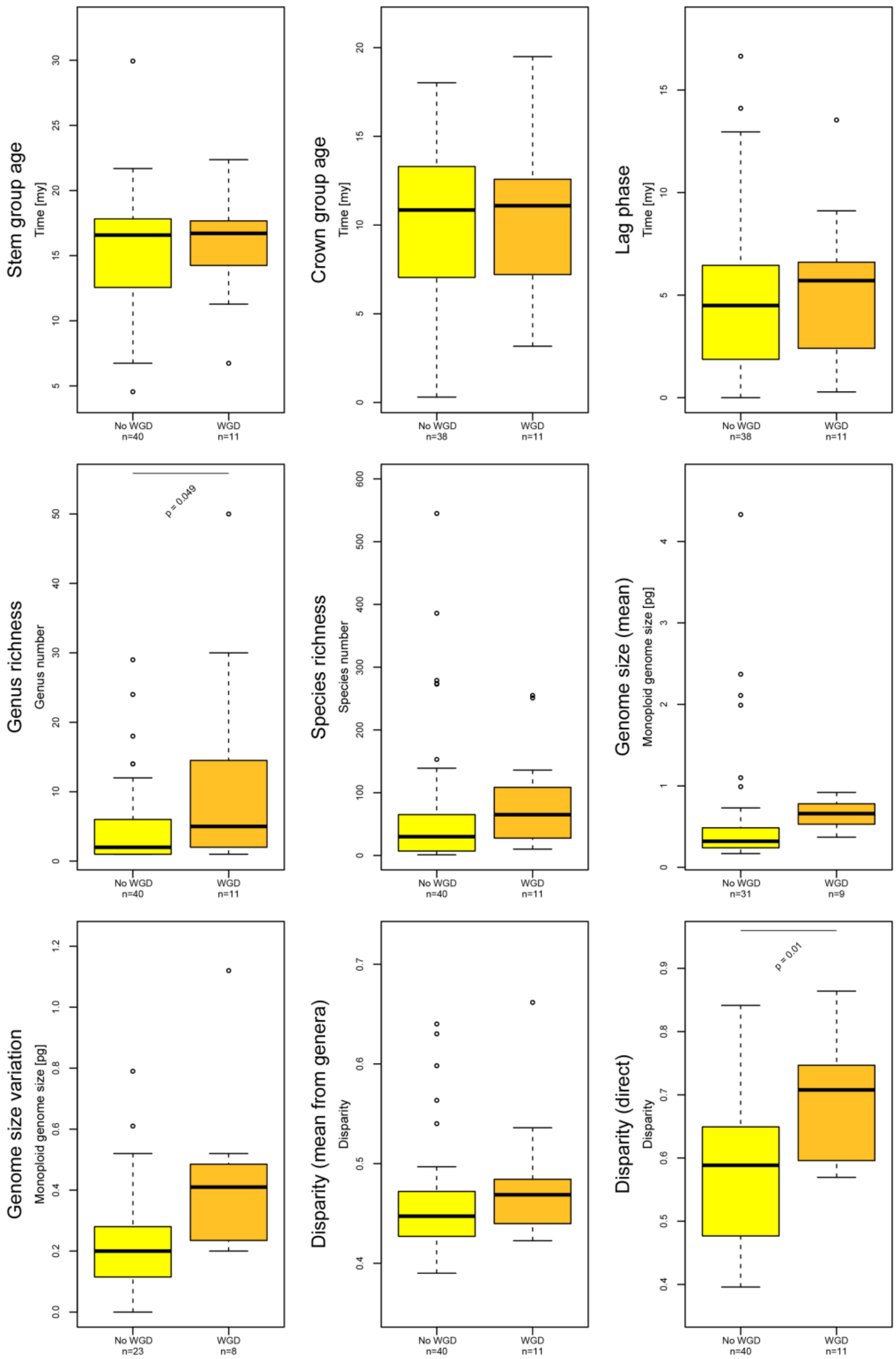

Supplementary Figure 9. Boxplot for comparison of Brassicaceae tribes with/without WGDs. Tribal values from Supplementary Table 2. In the boxplot, center line represents median; upper and lower quartiles are indicated by box limits; whiskers represent 1.5x interquartile range and points are outliers. Only number of genera and disparity (direct) show a significant difference between tribes with WGD and without WGD in a phylogenetic ANOVA (Supplementary Table 9). Phylogenetic ANOVA was performed with 1000 simulations and post-hoc comparisons (two-sided); *P*-values were adjusted using Bonferroni correction. Significant differences are indicated with bars above the boxplots. Source data are provided as a Source Data file.

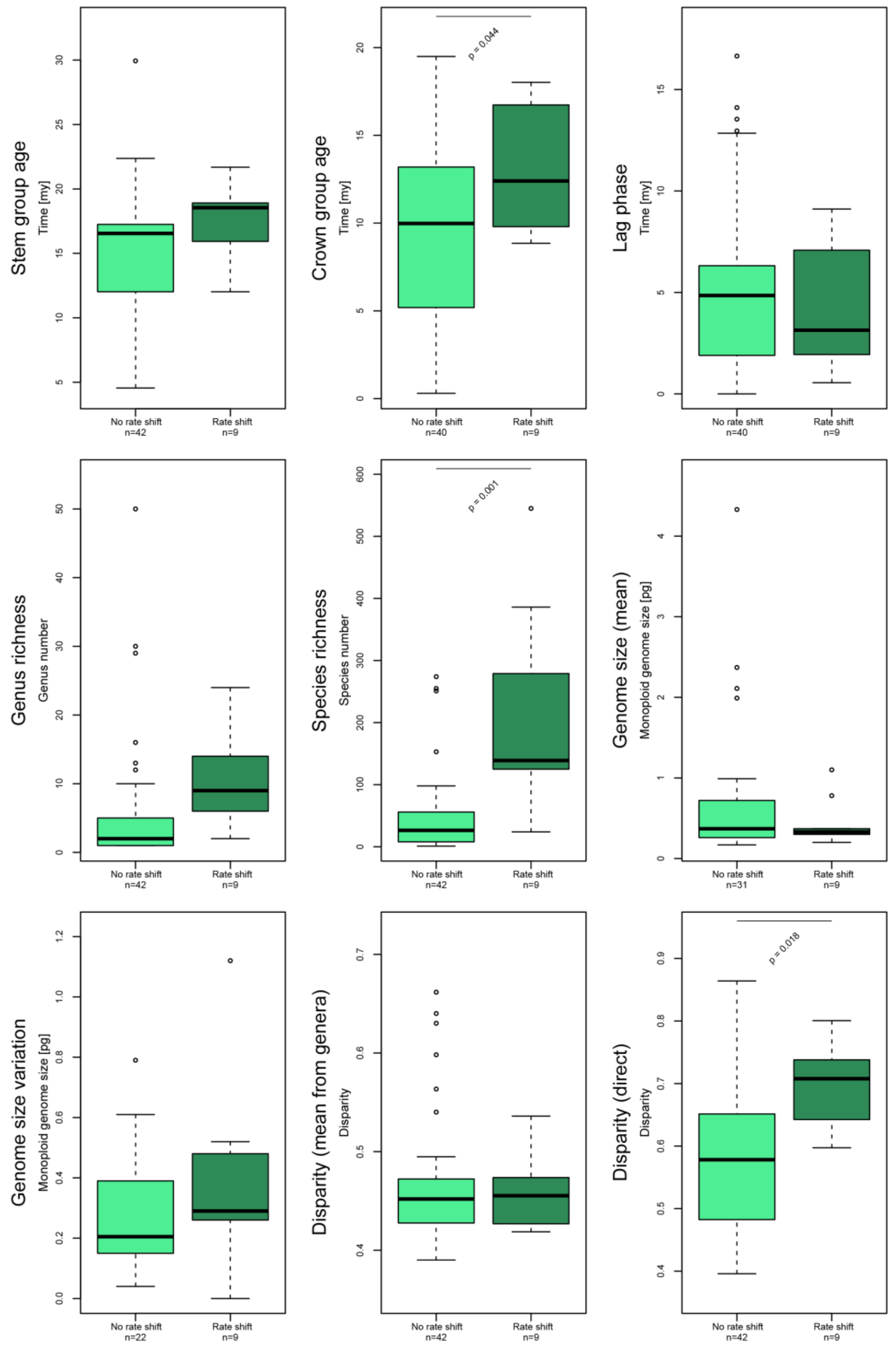

Supplementary Figure 10. Boxplot for comparison of Brassicaceae tribes with/without significant rate shifts. Tribal values from Supplementary Table 2 excluding basal tribe Aethionemeae. In the boxplot, center line represents median; upper and lower quartiles are indicated by box limits; whiskers represent 1.5x interquartile range and points are outliers. Only crown group age, number of species and disparity (direct) show a significant difference between tribes with rate shift and without rate shift in a phylogenetic ANOVA (Supplementary Table 9). Phylogenetic ANOVA was performed with 1000 simulations and post-hoc comparisons (two-sided); *P*-values were adjusted using Bonferroni correction. Significant differences are indicated with bars above the boxplots. Source data are provided as a Source Data file.

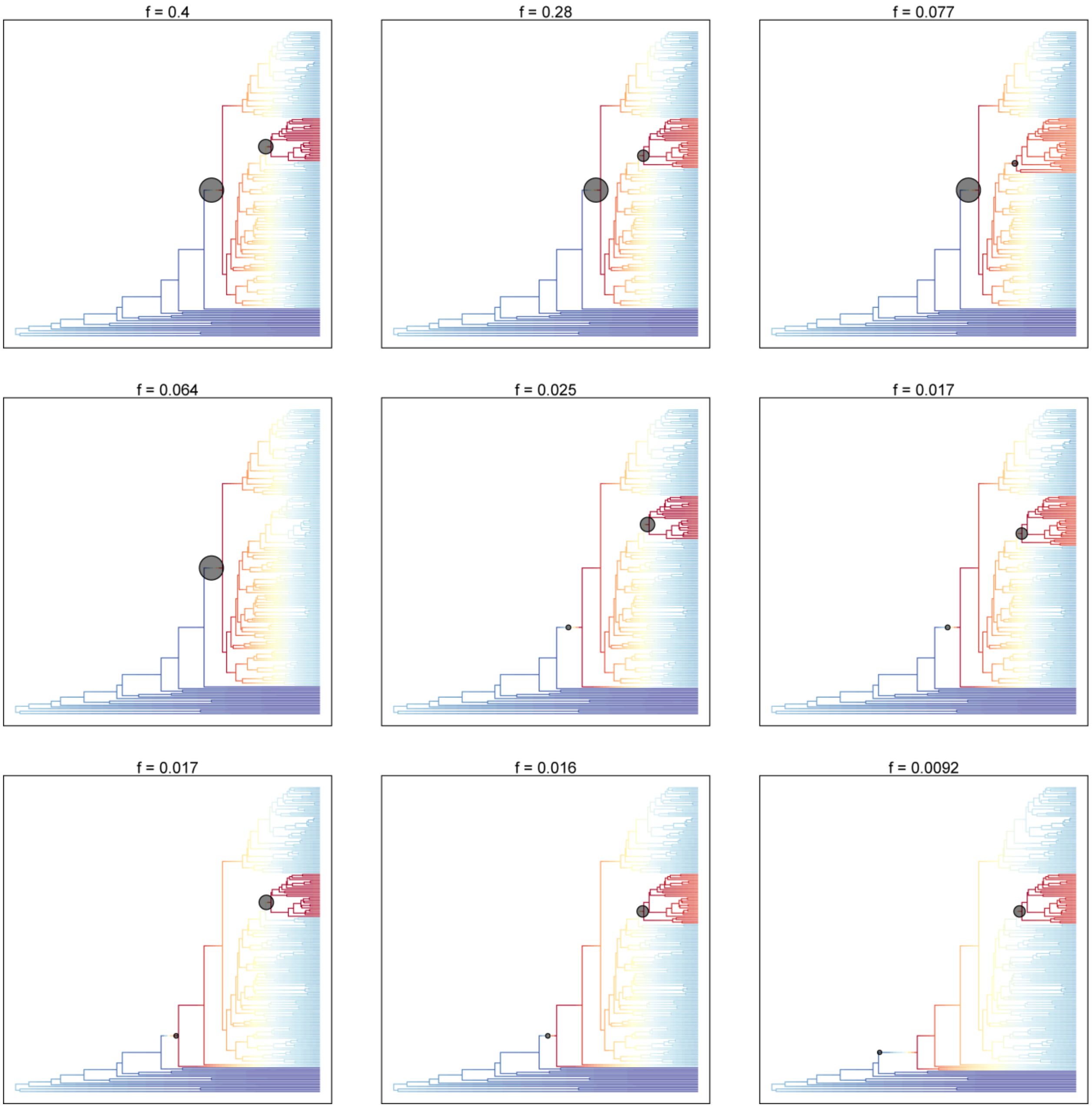

Supplementary Figure 11. BAMM credible shift sets. The nine highest probability credible shift sets are shown. Two shifts were detected in eight out of the nine sets, and only one shift in the other. The first shift was consistently located just before the onset of Brassicaceae lineage diversification, ca. 20 mya, or (with much lower frequency) at one of the splits just predating this diversification event, such as before the split of tribe Aethionemeae from the rest of Brassicaceae or at the split of Cleomaceae and Brassicaceae. The second shift, detected in eight shift sets, was located in or at the base of the clade of four tribes Brassiceae, Thelypodieae, Sisymbrieae and Isatideae (termed ‘lineage II’ in Franzke *et al.*[10]), or in one case also included remote taxa *Arabis josiae*, *Fourraea alpina* and *Schrenkiella parvula*. Source data are provided as a Source Data file.

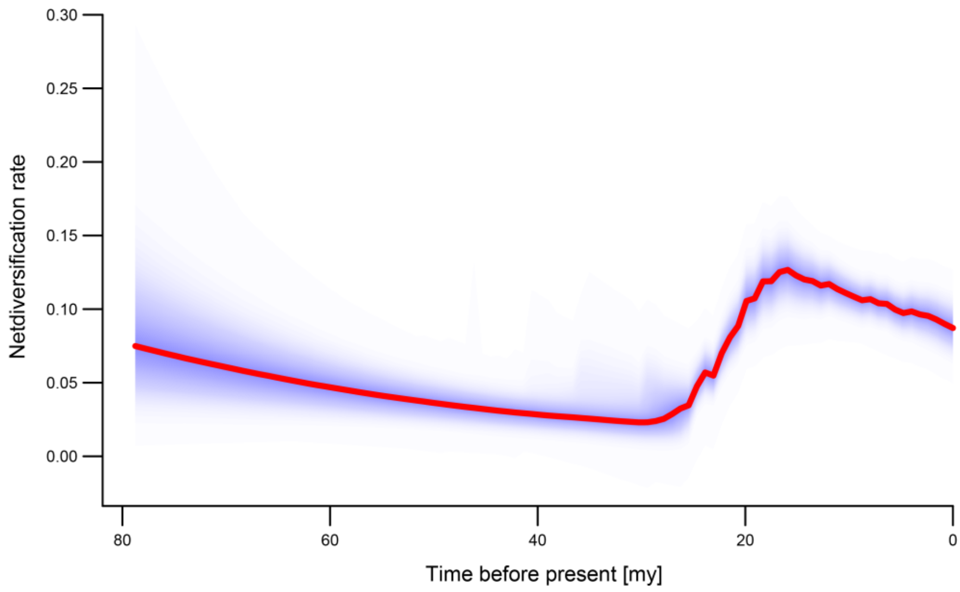

Supplementary Figure 12. Net diversification in Brassicaceae. Rate-through-time plot from BAMM based on genus data. The increased diversification rate 16-23 mya coincides with lineage diversification in Brassicaceae and the origin of most tribes. Source data are provided as a Source Data file.

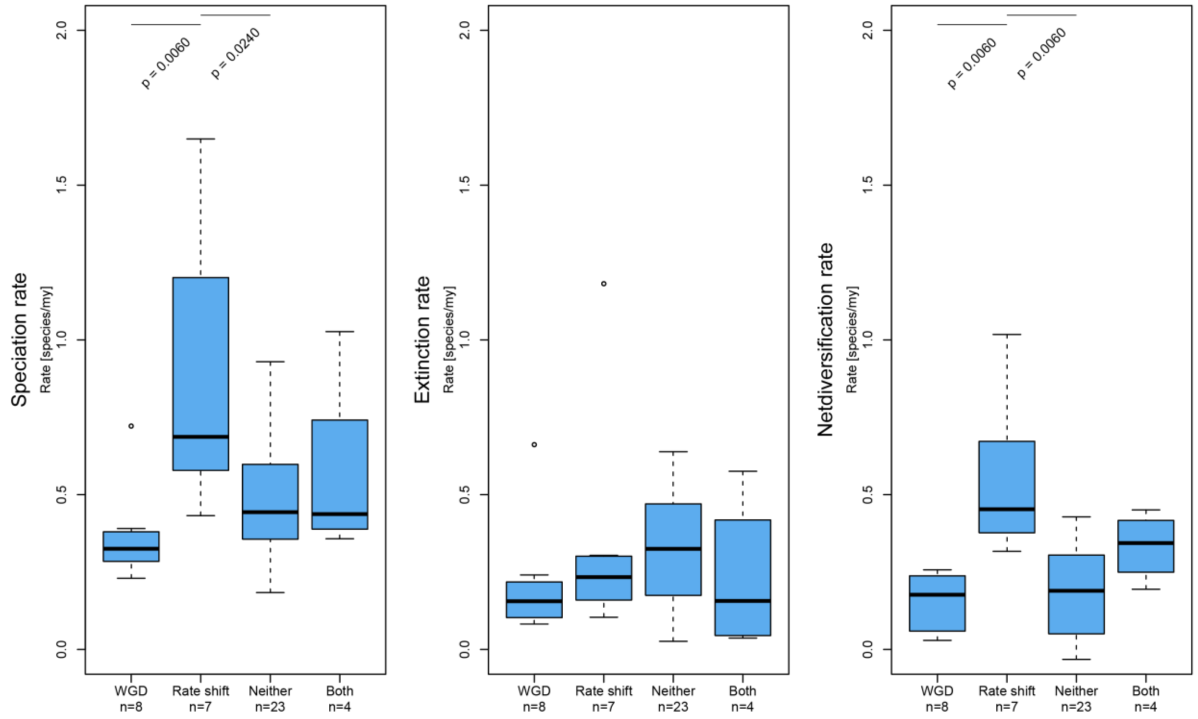

Supplementary Figure 13. Diversification of Brassicaceae tribes. Speciation, extinction and net diversification rates on tribal level (data taken from Huang *et al.*[71]) indicate a significant difference between tribes with rate shifts compared to those with WGD or neither WGD nor rate shift in a phylogenetic ANOVA for speciation and net diversification rate (Supplementary Table 11). In the boxplot, center line represents median; upper and lower quartiles are indicated by box limits; whiskers represent 1.5x interquartile range and points are outliers. WGD tribes show considerably lower extinction rate than tribes with rate shifts and tribes without both, WGD shift, but this is not significant in phylogenetic ANOVA. Phylogenetic ANOVA was performed with 1000 simulations and post-hoc comparisons (two-sided); *P*-values were adjusted using Bonferroni correction. Significant differences (see Supplementary Tables 12-14 for full test statistics) are indicated with bars above the boxplots. Source data are provided as a Source Data file.

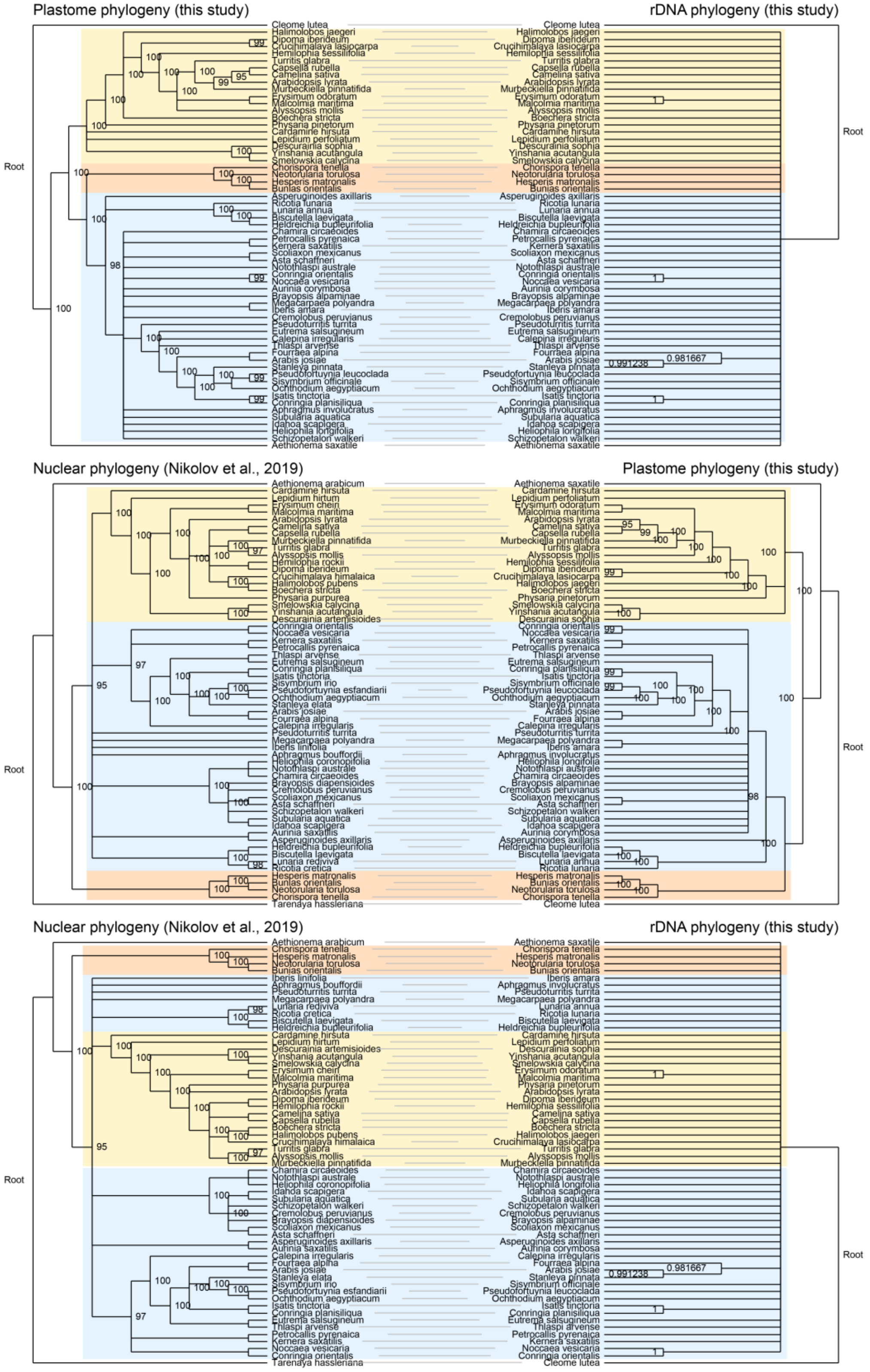

Supplementary Figure 14. Phylogenetic tree comparisons. Pairwise comparisons between the plastome phylogeny (this study), rDNA phylogeny (this study) and a recent nuclear phylogeny[5] are shown in tanglegrams. Trees were pruned to include only species present in all three sets, and some taxa in the nuclear phylogeny were substituted by closely related ones present in our sampling. Polytomies were introduced when support values were low (< 95%). Note that here, Transfer Bootstrap Expectation support values were used for the rDNA phylogeny – Felsenstein bootstrap values were below 95 for all nodes in the pruned trees. The three major lineages are indicated using colored background. Source data are provided as a Source Data file.

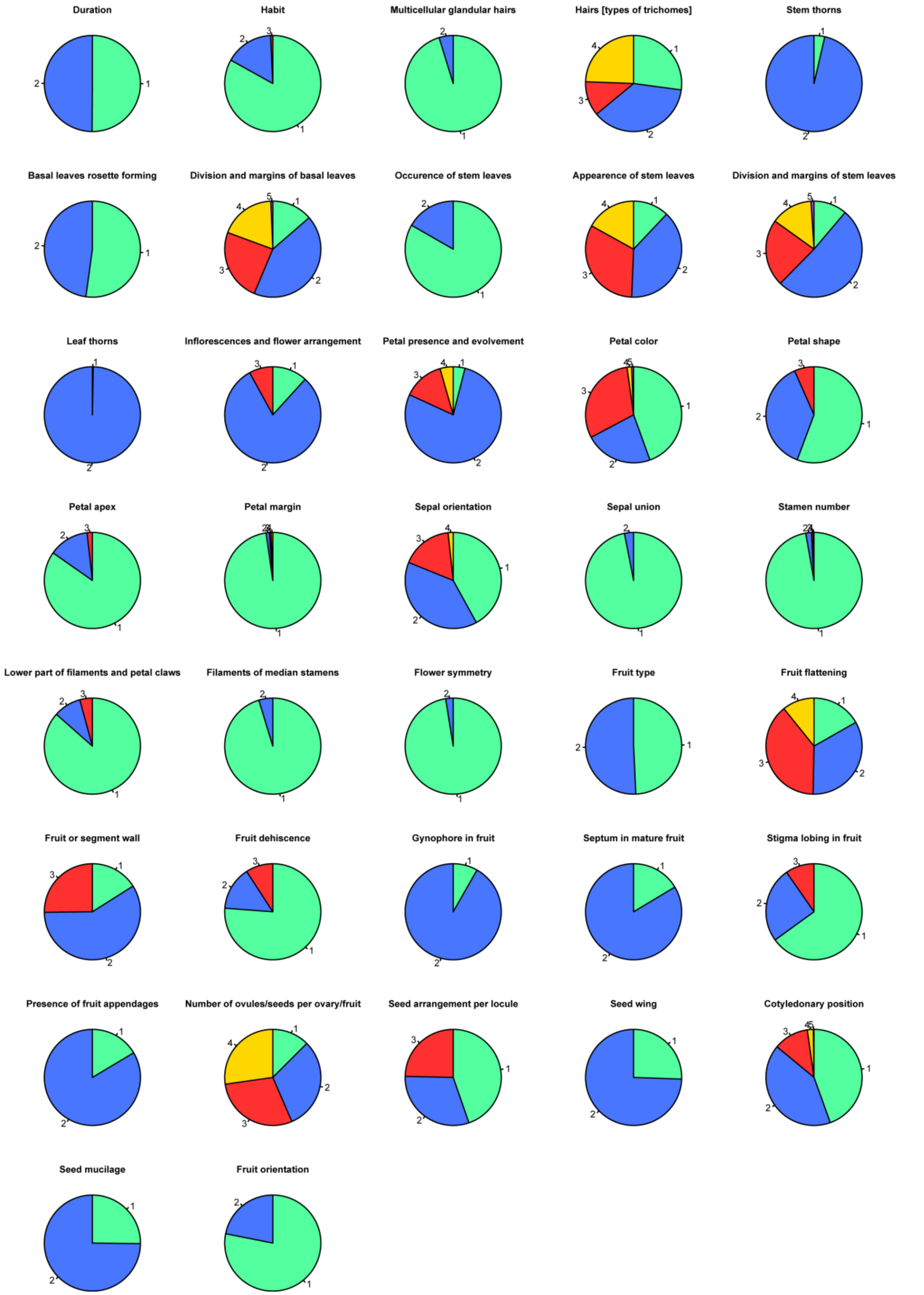

Supplementary Figure 15. Morphological characters and their states in the Brassicaceae family. Pie charts showing the frequency of character states for each character scored in the morphomatrix. All characters were coded to have no more than five states. Source data are provided as a Source Data file.

Supplementary Table 1. Mesopolyploidization.

	Lineage	Mesopoly-ploidization	Mandáková *et al.*[68]	Mandáková *et al.*[108]	Kiefer *et al.*[63]	Base chromosome number (excluding 'doubtful')	Range of base chromosome numbers (excluding 'doubtful')	% Neopolyploids	Mean genome size [pg]
**Alyssopsideae**	I	?				8	1	50	0.18
**Boechereae**	I	no			x	7	1	45	0.24
**Camelineae**	I	no			x	8	1	50	0.26
**Cardamineae**	I	no			x	8	1	63	0.31
**Crucihimalayeae**	I	?				8	1	0	0.32
**Descurainieae**	I	?				7	1	44	0.2
**Halimolobeae**	I	?				8	1	22	0.17
**Lepidieae**	I	no			x	8	1	73	0.3
**Oreophytoneae**	I	?				8	1	33	0.19
**Smelowskieae**	I	?				6	1	62	0.26
**Turritideae**	I	no			x	6	1	50	0.24
**Malcolmieae**	I	?				7,8	2	14	0.26
**Stevenieae**	I	meso			x	8,15	2	60	0.53
**Yinshanieae**	I	?				6,7	2	63	NA
**Microlepidieae**	I	meso	x			4–6	3	7	0.5
**Erysimeae**	I	no			x	6–9	4	66	0.33
**Physarieae**	I	meso	x		x	4–10	7	46	0.78
**Scoliaxoneae**	II	?				NA	NA	NA	NA
**Alysseae**	II	no			x	8	1	32	1.11
**Arabideae**	II	no			x	8	1	63	0.36
**Asteae**	II	?				10	1	0	NA
**Calepineae**	II	?				7	1	75	0.2
**Coluteocarpeae**	II	no			x	7	1	31	0.33
**Conringieae**	II	no			x	7	1	20	0.22
**Cremolobeae**	II	?				11	1	0	NA
**Eutremeae**	II	no			x	7	1	78	0.32
**Isatideae**	II	?				7	1	39	0.34
**Kernereae**	II	no			x	7	1	0	0.2
**Notothlaspideae**	II	?				19?	1	100	NA
**Sisymbrieae**	II	no			x	7	1	35	0.31
**Thlaspideae**	II	?				7	1	18	0.4
**Aphragmeae**	II	?				7,8	2	0	NA
**Cochlearieae**	II	meso	x		x	6,7	2	76	0.37
**Heliophileae**	II	meso	x		x	10,11	2	14	0.39
**Megacarpaeeae**	II	?				7,9	2	0	NA

**Schizopetaleae**	II	meso	x			9,10	2	0	NA
**Biscutelleae**	II	meso	x		x	6,8,9	3	19	0.92
**Eudemeae**	II	?				7,9,11	3	0	0.73
**Thelypodieae**	II	meso	x			10,11,13,14	4	9	0.71
**Anastaticeae**	II	meso	x	x	x	9-13	5	19	0.6
**Iberideae**	II	meso	x		x	7–11	5	27	0.66
**Brassiceae**	II	meso			x	7-12	6	28	0.85
**Shehbazieae**	III	?				NA	NA	NA	NA
**Buniadeae**	III	no		x		7	1	50	2.37
**Chorisporeae**	III	no		x		7	1	33	0.57
**Dontostemoneae**	III	no		x		7	1	33	1.99
**Euclidieae**	III	no		x	x	7	1	36	0.99
**Hesperideae**	III	no		x		6,7	2	29	4.33
**Anchonieae**	III	no		x		6-8	3	9	2.11
**Aethionemeae**	basal	no			x	11,12	2	44	0.37

Lineage assignments following Koch and Al-Shehbaz[9] is given. We obtained mesopolyploidization data from Mandáková *et al.*[68], Mandáková *et al.*[122] and Kiefer *et al.*[63]. Base chromosome numbers, percentage of polyploids and mean genome size were extracted from Hohmann *et al.*[2]. Within lineages, the table is sorted by the range of base chromosome numbers – tribes with multiple base chromosome numbers often have been shown to have gone through mesopolyploidizations. These tribes also generally have a higher mean genome size (compared to others in the same lineage). Genome sizes in lineage III are high, but the lack of mesopolyploidizations has been demonstrated[122].

Supplementary Table 2. Tribal level data.

	Lineages following Koch & Al-Shehbaz	Lineages following Franzke *et al.*	Lineages following Nikolov *et al.*	WGD	Rate shift	Stem group age [my]	Crown group age [my]	Lag-phase [my]	Number of genera	Number of species	Genome size (mean) [pg]	Genome size variation [pg]
**Camelineae**	I	I	I	0	0	7.9 (5.85-10.14)	7.58 (5.56-9.78)	0.32	6	32	0.26	0.2
**Oreophytoneae**	I	I	I	0	0	7.9 (5.85-10.14)	2.11 (0.83-3.51)	5.79	2	6	0.19	0.07
**Turritideae**	I	I	I	0	0	4.55 (1.86-7.26)	3.2 (1.7-5.1)	1.35	1	2	0.24	-
**Erysimeae**	I	I	I	0	0	6.96 (4.4-9.66)	1.72 (0.54-3.19)	5.24	1	274	0.33	0.27
**Malcolmieae**	I	I	I	0	0	6.96 (4.4-9.66)	2.3 (0.8-4.9)	4.66	1	6	0.26	-
**Alyssopsideae**	I	I	I	0	0	8.97 (6.64-11.44)	7.1 (3.8-11.4)	1.87	4	9	0.18	0.1
**Microlepidieae**	I	I	I	1	0	11.28 (8.4-14.31)	5.57 (2.9-8.58)	5.71	16	55	-	-
**Crucihimalayeae**	I	I	I	0	0	10.05 (7.07-13.01)	7.51 (4.49-10.51)	2.54	2	14	0.32	0.1
**Boechereae**	I	I	I	0	1	12.02 (9.31-15.03)	8.88 (5.96-11.84)	3.14	9	125	0.24	0
**Halimolobeae**	I	I	I	0	0	12.02 (9.31-15.03)	7.95 (5.05-11.08)	4.07	5	39	0.17	0.07
**Physarieae**	I	I	I	1	1	15.93 (12.65-19.61)	8.85 (5.34-12.55)	7.08	7	136	0.78	1.12
**Stevenieae**	I	I	I	1	0	17.94 (14.36-21.88)	12.87 (6.27-19.07)	5.07	2	10	0.53	-
**Cardamineae**	I	I	I	0	1	18.55 (14.78-22.37)	17.9 (12.6-22.1)	0.65	14	386	0.31	0.27
**Lepidieae**	I	I	I	0	1	18.77 (14.91-22.63)	10.89 (7.14-15.07)	7.88	3	273	0.3	0.52
**Smelowskieae**	I	I	I	0	0	13.15 (8.31-18.0)	8.69 (4.84-12.85)	4.46	1	25	0.26	0.16
**Yinshanieae**	I	I	I	0	0	13.15 (8.31-18.0)	0.3 (0.06-0.62)	12.85	1	4	-	-
**Descurainieae**	I	I	I	0	1	17.0 (12.95-21.46)	14.75 (10.65-18.99)	2.25	6	48	0.2	0.13
**Brassiceae**	II	II	II	1	0	12.57 (9.81-15.16)	12.29 (9.81-15.16)	0.28	50	251	0.85	0.45
**Thelypodieae**	II	II	II	1	0	6.74 (4.58-9.03)	4.81 (3.14-6.54)	1.93	30	255	0.71	0.43
**Sisymbrieae**	II	II	II	0	0	6.74 (4.58-9.03)	3.76 (2.08-5.65)	2.98	3	48	0.31	0.22
**Isatideae**	II	II	II	0	0	13.97 (11.21-17.14)	6.49 (3.62-9.62)	7.48	5	98	0.34	0.17
**Calepineae**	II	exp. II	II	0	0	18.39 (15.1-22.09)	13.1 (9.8-16.7)	5.29	6	32	0.2	-
**Eutremeae**	II	exp. II	II	0	0	17.25 (13.62-21.09)	10.8 (6.8-15.6)	6.45	2	42	0.32	-
**Thlaspideae**	II	exp. II	II	0	0	17.25 (13.62-21.09)	13.3 (9.4-17.1)	3.95	12	41	0.4	0.26
**Arabideae**	II	exp. II	IV	0	1	19.96 (16.41-23.54)	18.02 (14.59-21.63)	1.94	18	545	0.36	0.29
**Anastaticeae**	II	exp. II	-	1	0	16.91 (12.86-20.67)	11.09 (7.66-14.84)	5.82	13	65	0.6	0.52
**Megacarpeae**	II	exp. II	-	0	0	16.55 (12.54-20.39)	11.51 (6.87-16.21)	5.04	2	11	-	-
**Iberideae**	II	exp. II	-	1	0	16.55 (12.54-20.39)	15.56 (11.43-19.5)	0.99	2	31	0.66	0.39
**Hillielleae**	II	exp. II	-	0	0	17.13 (13.08-20.97)	12.6 (8.9-16.3)	4.53	1	11	-	-
**Cochlearieae**	II	exp. II	-	1	1	18.91 (15.12-22.88)	9.8 (5.69-14.53)	9.11	2	24	0.37	0.26
**Cremolobeae**	II	exp. II	V	0	0	16.61 (11.57-20.87)	16.41 (12.18-21.5)	0.2	2	8	-	-
**Asteae**	II	exp. II	V	0	0	16.61 (11.57-20.87)	16.41 (12.18-21.5)	0.2	1	2	-	-
**Scoliaxoneae**	II	exp. II	V	0	0	16.61 (11.57-20.87)	NA	NA	1	1	-	-
**Eudemeae**	II	exp. II	V	0	0	16.61 (11.57-20.87)	16.41 (12.18-21.5)	0.2	7	28	0.73	-
**Kernereae**	II	exp. II	II	0	0	16.71 (12.05-21.68)	3.75 (1.61-6.1)	12.96	3	3	0.2	-

**Schizopetaleae**	II	exp. II	V	1	0	16.71 (12.05-21.68)	3.17 (0.88-6.37)	13.54	2	17	-	-
**Heliophileae**	II	exp. II	V	1	0	17.4 (13.16-21.59)	11.27 (7.14-15.67)	6.13	1	81	0.39	0.2
**Notothlaspideae**	II	exp. II	V	0	0	18.61 (14.35-22.62)	4.5 (0.8-8.3)	14.11	1	2	-	-
**Coluteocarpeae**	II	exp. II	II	0	1	13.1 (8.3-7.19)	12.4 (9.7-15.4)	0.7	14	139	0.33	0.37
**Conringieae**	II	exp. II	II	0	0	13.1 (8.3-7.19)	13 (8.76-17.62)	0.1	2	9	0.22	-
**Aphragmeae**	II	exp. II	II	0	0	20.08 (16.39-23.93)	13.9 (7.98-19.21)	6.18	1	14	-	-
**Alysseae**	II	exp. II	IV	0	1	21.69 (18.07-25.61)	16.73 (12.78-21.03)	4.96	24	279	1.1	0.48
**Biscutelleae**	II	exp. II	-	1	0	22.37 (18.46-26.56)	19.49 (14.62-24.45)	2.88	5	73	0.92	0.21
**Anchonieae**	III	III	III	0	0	16.28 (12.28-20.42)	15.2 (11.5-18.8)	1.08	10	73	2.11	0.22
**Hesperideae**	III	III	III	0	0	16.28 (12.28-20.42)	7.77 (3.79-12.52)	8.51	2	50	4.33	0.04
**Buniadeae**	III	III	III	0	0	17.02 (12.29-21.2)	7.05 (2.69-12.52)	9.97	1	2	2.37	0.16
**Euclidieae**	III	III	III	0	0	18.69 (14.56-22.83)	13.3 (9-17.7)	5.39	29	153	0.99	0.61
**Chorisporeae**	III	III	III	0	0	14.67 (10.55-18.88)	12.21 (8.21-15.83)	2.46	4	56	0.57	0.79
**Shehbazieae**	III	III	-	0	0	14.67 (10.55-18.88)	NA	NA	1	1	-	-
**Dontostemoneae**	III	III	III	0	0	19.95 (15.97-24.44)	9.16 (5.1-13.4)	10.79	2	17	1.99	-
**Aethionemeae**	basal	basal	basal	0	0	29.94 (24.31-35.71)	13.29 (7.76-19.28)	16.65	1	57	0.37	0.15

Lineage assignments following Koch & Al-Shehbaz[9], Franzke *et al.*[10] and Nikolov *et al.*[5] are given. Ten tribes underwent mesopolyploidizations (WGDs)[63,68], and significant shifts in diversification rate were detected in nine tribes[71]. Stem group ages for all tribes and crown group ages for 24 tribes were extracted from our plastome divergence time estimates (Supplementary Figure 2), and crown group ages for the other tribes added from tribal estimates[71] are underlined. Genome size and genome size variation were recalculated using current taxonomy based on previously published data[2].

Supplementary Table 3. Character differences.

	Mean disparity (tribes direct)	Mean disparity (tribes from genera)	Cumulative contribution DAPC (with Aethionemeae)	Cumulative contribution DAPC (without Aethionemeae)	Lineage differen-tiation	Phylogenetic signal (Moran's I)	Phylogenetic signal (Moran's I *P*-value)
**A_01**	0.9135	0.7218	0.0526	0.0180		-0.0258	0.8921
**A_02**	0.5064	0.4097	0.0413	0.0360		-0.0125	0.0929
**B_03**	0.5385	0.5149	0.2813	0.2745	I/III, II/III	-0.0025	0.0130
**B_04**	0.6106	0.4276	0.0949	0.0837	I/II	-0.0085	0.0460
**C_05**	0.5577	0.5203	0.1266	0.0074		-0.0154	0.1499
**D_06**	0.8846	0.6588	0.0449	0.0043		-0.0210	0.4845
**D_07**	0.5808	0.3831	0.0370	0.0169		-0.0146	0.1429
**D_08**	0.7500	0.5976	0.0404	0.0283		-0.0256	0.8841
**D_09**	0.6731	0.4464	0.0090	0.0012		-0.0234	0.7043
**D_10**	0.5462	0.3683	0.0281	0.0235		-0.0145	0.1349
**D_11**	0.5096	0.5000	0.0238	0.0086		-0.0196	0.3227
**E_12**	0.5897	0.4059	0.0401	0.0326		-0.0149	0.1608
**E_13**	0.4615	0.2997	0.0820	0.0756		-0.0140	0.1309
**E_14**	0.5385	0.3809	0.1060	0.0897		-0.0180	0.2737
**E_15**	0.7244	0.5091	0.1388	0.0204		-0.0190	0.3447
**E_16**	0.5256	0.3882	0.0841	0.0095		-0.0124	0.0909
**E_17**	0.2837	0.2533	0.0054	0.0069		-0.0277	0.9690
**E_18**	0.5913	0.4010	0.2237	0.1611	II/III	-0.0062	0.0380
**E_19**	0.5673	0.5203	0.0601	0.0566		-0.0133	0.1279
**E_20**	0.2933	0.2605	0.0136	0.0041		-0.0161	0.2218
**E_21**	0.4679	0.3719	0.0565	0.0221		-0.0215	0.5225
**E_22**	0.5769	0.5179	0.2036	0.1091		-0.0162	0.1838
**E_23**	0.5577	0.5113	0.1649	0.1562		-0.0078	0.0340
**F_24**	0.7981	0.5914	0.0627	0.0805		-0.0157	0.1678
**F_25**	0.6538	0.4138	0.0603	0.0524		-0.0142	0.1409
**F_26**	0.5705	0.3904	0.0138	0.0086		-0.0166	0.2448
**F_27**	0.5385	0.3726	0.0673	0.0723	I/II	-0.0093	0.0599
**F_28**	0.6635	0.5509	0.0819	0.0699		-0.0143	0.1399
**F_29**	0.7404	0.5836	0.1227	0.1061		-0.0201	0.4186
**F_30**	0.5641	0.3831	0.1945	0.0254	I/III, II/III	-0.0069	0.0360
**F_31**	0.6827	0.5326	0.0518	0.0449		-0.0103	0.0689
**F_32**	0.7163	0.4737	0.0298	0.0359	I/II	-0.0179	0.2717
**F_33**	0.7308	0.4692	0.1577	0.1471	I/III, II/III	-0.0194	0.3437
**F_34**	0.7885	0.6040	0.0731	0.0430	I/II	-0.0077	0.0400
**F_35**	0.3654	0.2452	0.0041	0.0008		-0.0133	0.1269
**F_36**	0.7404	0.5797	0.0817	0.0538	II/III	-0.0171	0.2338
**F_37**	0.8462	0.6434	0.0399	0.0131		-0.0197	0.3856

Mean disparity, DAPC contribution, lineage differentiation and phylogenetic signal for all 37 characters. The top five characters, where character states can be used to discriminate between lineages (pairwise comparison), are A_01, D_06, F_24, F_34 and F_37 for disparity calculated from tribes and A_01, D_06, D_08, F_34 and F_37 for disparity calculated from genus data. The highest DAPC contribution was found in B_03, E_18, E_22, E_23 and F_30 when including Aethionemeae in the analysis and B_03, E_18, E_22, E_23 and F_33 when excluding this tribe. Phylogenetic signal in disparity using Moran's I[123] was detected in six characters with significant *P*-values, namely B_03, B_04, E_18, E_23, F_30 and F_34. Lineage differentiation is often associated with high DAPC contribution and with phylogenetic signal, but medium disparity values.

Supplementary Table 4. Lineage differences.

	Lineages I, II, III (Koch & Al-Shehbaz)				Lineages I, II, expII, III (Franzke *et al.*)				Lineages I, II, III, IV, V (Nikolov *et al.*)			
	**n**	**df**	**X^2**	***P*-value**	**n**	**df**	**X^2**	***P*-value**	**n**	**df**	**X^2**	***P*-value**
**Stem group age**	50	2	9.3924	0.0091	50	3	19.3572	0.0002	42	4	14.2069	0.0067
**Crown group age**	48	2	7.2791	0.0263	48	3	12.056	0.0072	41	4	9.5113	0.0495
**Lag-phase**	48	2	1.4054	0.4952	48	3	1.9019	0.593	41	4	1.4767	0.8308
**Number of genera**	50	2	0.2345	0.8894	50	3	4.1527	0.2454	42	4	9.7448	0.0450
**Number of species**	50	2	0.044	0.9782	50	3	4.3783	0.2234	42	4	9.9355	0.0415
**Mean genome size**	39	2	18.2506	0.0001	39	3	18.4389	0.0004	31	4	18.9928	0.0008
**Genome size variation**	30	2	4.1355	0.1265	30	3	4.1902	0.2416	17	4	4.1440	0.3869
**Disparity from genera**	50	2	4.8943	0.0865	50	3	4.9249	0.1774	42	4	9.5980	0.0478
**Disparity direct**	50	2	0.042	0.9792	50	3	2.6083	0.456	42	4	6.8370	0.1448

Kruskal-Wallis rank-sum tests for differences between lineages. Three different lineage assignments were tested, with either three[9], four[10] or five[5] lineages. Not all tribes were assigned to a lineage in the latter and were excluded from the analyses. The first diverging tribe Aethionemeae, which is not part of any lineage, was excluded from all analyses. Pairwise Wilcoxon rank-sum tests for significant tests are given in Supplementary Tables 5-7.

Supplementary Table 5. Lineage differences.

	**n**	**I/II**	**I/III**	**II/III**
**Stem group age**	50	0.017	0.056	1
**Crown group age**	48	0.029	0.405	1
**Mean genome size**	39	0.018	0.002	0.007

Pairwise Wilcoxon rank-sum test for differences between three lineages[9] with significant differences detected in Kruskal-Wallis test (Supplementary Table 4). *P*-values from two-sided tests with Bonferroni correction are given for pairwise comparisons.

Supplementary Table 6. Lineage differences.

	**n**	**I/II**	**I/expII**	**I/III**	**II/expII**	**II/III**	**expII/III**
**Stem group age**	50	1	0.003	0.112	0.019	0.061	1
**Crown group age**	48	1	0.012	0.811	0.351	0.686	1
**Mean genome size**	39	0.164	0.116	0.004	1	0.229	0.02

Pairwise Wilcoxon rank-sum test for differences between four lineages[10] with significant differences detected in Kruskal-Wallis test (Supplementary Table 4). *P*-values from two-sided tests with Bonferroni correction are given for pairwise comparisons.

Supplementary Table 7. Lineage differences.

	**n**	**I/II**	**I /III**	**I/IV**	**I/V**	**II/III**	**II/IV**	**II/V**	**III/IV**	**III/V**	**IV/V**
**Stem group age**	42	1	0.11	0.29	0.12	1	0.6	1	0.65	1	0.47
**Crown group age**		1	0.98	0.26	1	1	0.38	1	0.71	1	0.6
**Number of genera**	42	1	1	0.28	1	1	1	0.21	1	1	0.46
**Number of species**	42	1	1	0.42	0.76	1	0.26	0.3	0.71	1	0.56
**Mean genome size**	31	1	0.0082	0.4637	0.6685	0.0284	1	1	1	1	1

Pairwise Wilcoxon rank-sum test for differences between five lineages[5] with significant differences detected in Kruskal-Wallis test (Supplementary Table 4). *P*-values from two-sided tests with Bonferroni correction are given for pairwise comparisons. Because of the small sample size in some lineages, particularly in lineage IV with only two tribes, only few tests were significant.

Supplementary Table 8. Association of tribal level data.

			P-value								
		n	Stem group age	Crown group age	Lag-phase	Number of genera	Number of species	Mean genome size	Genome size variation	Disparity from genera	Disparity direct
Adjusted R^2	**Stem group age**	51	-	0.0004	0.0078	0.6680	0.0974	0.8179	0.9213	0.3920	0.1671
	**Crown group age**	49	0.2190	-	0.0000	0.0611	0.0052	0.4577	0.6645	0.6096	0.0006
	**Lag-phase**	49	0.1227	0.3819	-	0.0086	0.0966	0.3011	0.7053	0.2494	0.0034
	**Number of genera**	51	-0.0165	0.0529	0.1197	-	0.0000	0.4975	0.1573	0.0400	0.0000
	**Number of species**	51	0.0358	0.1364	0.0376	0.2873	-	0.9945	0.0831	0.7454	0.0000
	**Mean genome size**	40	-0.0249	-0.0113	0.0025	-0.0138	-0.0263	-	0.8417	0.8459	0.8732
	**Genome size variation**	31	-0.0341	-0.0277	-0.0293	0.0356	0.0690	-0.0330	-	0.5108	0.0159
	**Disparity from genera**	51	-0.0051	-0.0156	0.0074	0.0646	-0.0182	-0.0253	-0.0189	-	0.3259
	**Disparity direct**	51	0.0190	0.2075	0.1510	0.4176	0.4202	-0.0256	0.1563	-0.0003	-

Phylogenetically Independent Contrasts[124] (PIC) was used to account for phylogenetic dependency of data. Adjusted R^2 (lower part) and *P*-value (upper part) are given.

Supplementary Table 9. Mesopolyploidization and diversification rate shifts.

		Mesopolyploidization/WGD				Significant rate shift			
	n	n (WGD)	n (no WGD)	F	*P*-value	n (rate shift)	n (no rate shift)	F	*P*-value
**Stem group age**	51	11	40	0.1813	0.67	9	42	2.2148	0.138
**Crown group age**	49	11	38	0.0493	0.847	9	40	4.4201	0.044
**Lag-phase**	49	11	38	0.0598	0.82	9	40	0.4898	0.491
**Number of genera**	51	11	40	4.4848	0.049	9	42	2.1233	0.169
**Number of species**	51	11	40	0.1858	0.642	9	42	25.0050	0.001
**Mean genome size**	40	9	31	0.0027	0.971	9	31	0.8501	0.348
**Genome size variation**	31	8	23	4.8529	0.092	9	22	1.6441	0.238
**Disparity from genera**	51	11	40	0.8176	0.356	9	42	0.1429	0.732
**Disparity direct**	51	11	40	8.1415	0.01	9	42	6.4449	0.018

Phylogenetic ANOVA for differences between tribes with and without mesopolyploidization/WGD events and significant shifts in diversification rates. For each analysis, we tested for significant differences between tribal data (from Supplementary Table 2) using phylogenetic ANOVA to account for statistical non-independence of data[125] with 1000 simulations and post-hoc comparisons (two-sided); *P*-values were adjusted using Bonferroni correction.

Supplementary Table 10. Disparity and speciation rates.

	**Estimate**	***P*-value**
**A_01**	-0.0204	0.6527
**A_02**	0.1429	0.9146
**B_03**	-0.0825	0.9092
**B_04**	0.0025	0.8671
**C_05**	-0.0740	0.8758
**D_06**	0.0451	0.4297
**D_07**	0.1903	0.4137
**D_08**	-0.0414	0.8776
**D_09**	-0.0107	0.6727
**D_10**	0.2844	0.4211
**D_11**	NA	NA
**E_12**	-0.0399	0.8746
**E_13**	0.0805	0.4190
**E_14**	0.0011	0.6925
**E_15**	0.0099	0.8529
**E_16**	0.0004	0.8666
**E_17**	-0.0305	0.9096
**E_18**	0.0493	0.9125
**E_19**	0.1316	0.4534
**E_20**	-0.0878	0.9105
**E_21**	0.0389	0.9316
**E_22**	0.0575	0.9288
**E_23**	0.0172	0.4842
**F_24**	0.0482	0.4659
**F_25**	0.0761	0.4112
**F_26**	0.0513	0.4609
**F_27**	-0.0145	0.9048
**F_28**	0.2315	0.4400
**F_29**	-0.1449	0.3726
**F_30**	0.0377	0.4614
**F_31**	-0.0447	0.8826
**F_32**	-0.0132	0.6583
**F_33**	-0.1905	0.3942
**F_34**	-0.1062	0.8991
**F_35**	-0.0284	0.6996
**F_36**	-0.0493	0.4674
**F_37**	0.0538	0.9033
**mean**	0.0376	0.8919

Results from traitDependentBAMM analysis for all characters separately and for mean disparity are given. Spearman correlation with two-tailed test was conducted for genus level disparity and using the plastome phylogeny pruned to include only one species per (monophyletic) genus, and estimates as well as *P*-values are given. Character D_11 (‘Leaf thorns’) was not variable in any genus included in our plastome tree, thus no correlation could be estimated. None of the characters showed a significant association between disparity and speciation rate.

Supplementary Table 11. Diversification rates in Brassicaceae tribes.

		Speciation		Extinction		Net diversification	
	**n**	**mean**	**sd**	**mean**	**sd**	**mean**	**sd**
**WGD**	8	0.3666	0.1526	0.2122	0.1895	0.1544	0.0927
**Shift**	7	0.9040	0.4519	0.3489	0.3756	0.5552	0.2759
**Rate shift & WGD**	4	0.5649	0.3106	0.2316	0.2510	0.3332	0.1101
**Neither**	23	0.5023	0.2166	0.3179	0.1762	0.1844	0.1439

Summary of various diversification rates from Huang *et al*.[71] grouped by tribes with WGD, rate shifts, both (WGD and rate shifts) and neither; data is also shown as box plots in Supplementary Figure 13. Rates are given in species/million years.

Supplementary Table 12. Differences in speciation rates.

			P-value			
		**n**	**Rate shift & WGD**	**Neither**	**Rate shift**	**WGD**
**Pairwise t-value**	**Rate shift & WGD**	4	-	1.0000	0.3180	1.0000
	**Neither**	23	-0.4327	-	0.0240	1.0000
	**Rate shift**	7	2.0266	3.4856	-	0.0060
	**WGD**	8	-1.2126	1.2380	-3.8891	-

We tested for significant differences in speciation rates between tribes grouped by the presence of WGDs, shifts in diversification rate, both, or neither, using phylogenetic ANOVA to account for statistical non-independence of data[125] with 1000 simulations and post-hoc comparisons (two-sided); *P*-values were adjusted using Bonferroni correction. Pairwise post-hoc test for phylogenetic ANOVA is shown. F was 6.240589, *P*-value 0.002 in the original phylogenetic ANOVA.

Supplementary Table 13. Differences in extinction rates.

			P-value			
		**n**	**Rate shift & WGD**	**Neither**	**Rate shift**	**WGD**
**Pairwise t-value**	**Rate shift & WGD**	4	-	1.0000	1.0000	1.0000
	**Neither**	23	0.6998	-	1.0000	1.0000
	**Rate shift**	7	0.8218	0.3151	-	1.0000
	**WGD**	8	-0.1392	-1.1313	-1.1600	-

We tested for significant differences in extinction rates between tribes grouped by the presence of WGDs, shifts in diversification rate, both, or neither, using phylogenetic ANOVA to account for statistical non-independence of data[125] with 1000 simulations and post-hoc comparisons (two-sided); *P*-values were adjusted using Bonferroni correction. Pairwise post-hoc test for phylogenetic ANOVA is shown. Significant comparisons ($P < 0.05$) are highlighted in bold. F was 1.394899, *P*-value 0.249 in the original phylogenetic ANOVA.

Supplementary Table 14. Differences in net diversification rates.

			P-value			
		**n**	**Rate shift & WGD**	**Neither**	**Rate shift**	**WGD**
**Pairwise t-value**	**Rate shift & WGD**	4	-	0.6540	0.2280	0.3480
	**Neither**	23	-1.6864	-	0.0060	1.0000
	**Rate shift**	7	2.1735	5.2724	-	0.0060
	**WGD**	8	-1.7924	-0.4484	-4.7531	-

We tested for significant differences in net diversification rates between tribes grouped by the presence of WGDs, shifts in diversification rate, both, or neither, using phylogenetic ANOVA to account for statistical non-independence of data[125] with 1000 simulations and post-hoc comparisons (two-sided); *P*-values were adjusted using Bonferroni correction. Pairwise post-hoc test for phylogenetic ANOVA is shown. F was 10.592749, *P*-value 0.001 in the original phylogenetic ANOVA.

Supplementary Table 15. Diversification rates raw data.

	Patterns	Speciation	Extinction	Net diversification
**Camelineae**	NONE	0.5371	0.3079	0.2291
**Oreophytoneae**	NONE	NA	NA	NA
**Turritideae**	NONE	NA	NA	NA
**Erysimeae**	NONE	0.5095	0.0809	0.4285
**Malcolmieae**	NONE	NA	NA	NA
**Alyssopsideae**	NONE	0.4435	0.389	0.0545
**Microlepidieae**	WGD	0.3096	0.0822	0.2274
**Crucihimalayeae**	NONE	0.785	0.5734	0.2116
**Boechereae**	SHIFT	1.2219	0.2042	1.0177
**Halimolobeae**	NONE	0.7683	0.4654	0.3029
**Physarieae**	BOTH	1.0268	0.5757	0.4511
**Stevenieae**	WGD	0.2301	0.1713	0.0587
**Cardamineae**	SHIFT	0.4324	0.1154	0.317
**Lepidieae**	SHIFT	1.6492	1.1816	0.4677
**Smelowskieae**	NONE	0.6325	0.3256	0.3069
**Yinshanieae**	NONE	0.2743	0.2648	0.0096
**Descurainieae**	SHIFT	0.6524	0.299	0.3534
**Brassiceae**	BOTH	0.4197	0.037	0.3826
**Thelypodieae**	BOTH	0.3577	0.053	0.3046
**Sisymbrieae**	WGD	0.3906	0.1955	0.1951
**Isatideae**	NONE	0.9297	0.528	0.4017
**Calepineae**	NONE	0.1992	0.1712	0.0279
**Eutremeae**	NONE	0.4184	0.2142	0.2042
**Thlaspideae**	NONE	0.2632	0.1229	0.1403
**Arabideae**	SHIFT	1.1809	0.304	0.8769
**Anastaticeae**	WGD	0.3699	0.1213	0.2486
**Megacarpeae**	NONE	0.4434	0.4753	-0.0318
**Iberideae**	WGD	0.2701	0.2408	0.0293
**Hillielleae**	NONE	0.4718	0.5038	-0.0319
**Cochlearieae**	BOTH	0.4552	0.2606	0.1946
**Cremolobeae**	NONE	0.4314	0.3251	0.1063
**Asteae**	NONE	NA	NA	NA
**Scoliaxoneae**	NONE	NA	NA	NA
**Eudemeae**	NONE	0.5636	0.4146	0.149
**Kernereae**	NONE	NA	NA	NA
**Schizopetaleae**	WGD	0.7217	0.6616	0.0601
**Heliophileae**	WGD	0.3415	0.0843	0.2572
**Notothlaspidieae**	NONE	NA	NA	NA
**Coluteocarpeae**	SHIFT	0.6869	0.2337	0.4531
**Conringieae**	NONE	0.1838	0.137	0.0468
**Aphragmeae**	NONE	0.4669	0.4202	0.0468
**Alysseae**	SHIFT	0.5046	0.1041	0.4005
**Biscutelleae**	WGD	0.299	0.1403	0.1587
**Anchonieae**	NONE	0.3678	0.1783	0.1895
**Hesperideae**	NONE	0.8791	0.476	0.4031
**Buniadeae**	NONE	NA	NA	NA
**Euclidieae**	NONE	0.3449	0.0264	0.3185
**Chorisporeae**	NONE	0.4434	0.0771	0.3663
**Shehbazieae**	NONE	NA	NA	NA
**Dontostemoneae**	NONE	0.3271	0.1956	0.1314
**Aethionemeae**	NONE	0.8681	0.6388	0.2294

Speciation, extinction and net diversification rates from Huang *et al.*[71] are shown. The presence of WGDs[63,68], rate shifts[71] (including the one detected in Clade Brassiceae+Thelypodieae+Sisymbrieae detected in this study), both events, or neither, is given as well.

Supplementary Table 16. Fossil calibration.

Split for calibration	Fossil	Minimum age for calibration
Prunus/*Malus*	*Prunus cathybrownae* (Rosales)[126]	48.4 my[127]
Castanea/*Cucumis*	*Bedellia* (Fagales)[128,129]	84 my[127]
Mangifera/*Citrus*	Unnamed (Sapindales)[130]	65 my[131]
Oenothera/*Eucalyptus*	*Esqueiria futabensis* (Myrtales)[132]	88.2 my[131]

Splits for calibration, name and order of the fossil their references are given. Following a previous study using outgroup fossil calibration for Brassicaceae[2], we selected four fossil constraints which are widely used in angiosperm divergence time estimation[127,131] and consistent with Magallón *et al*.[133]. Fossil ages were implemented as minimum age for calibration with a uniform distribution.

Supplementary References

1. Edger, P. P. *et al.* Brassicales phylogeny inferred from 72 plastid genes: A reanalysis of the phylogenetic localization of two paleopolyploid events and origin of novel chemical defenses. *American Journal of Botany* **105**, 463–469 (2018).
2. Hohmann, N., Wolf, E. M., Lysak, M. A. & Koch, M. A. A time-calibrated road map of Brassicaceae species radiation and evolutionary history. *The Plant Cell* **27**, 2770–2784 (2015).
3. Edger, P. P. *et al.* The butterfly plant arms-race escalated by gene and genome duplications. *Proc Natl Acad Sci USA* **112**, 8362–8366 (2015).
4. Guo, X. *et al.* Plastome phylogeny and early diversification of Brassicaceae. *BMC Genomics* **18**, e176 (2017).
5. Nikolov, L. A. *et al.* Resolving the backbone of the Brassicaceae phylogeny for investigating trait diversity. *New Phytologist* (2019) doi:10.1111/nph.15732.
6. Li, H.-T. *et al.* Origin of angiosperms and the puzzle of the Jurassic gap. *Nat. Plants* **5**, 461–470 (2019).
7. Kagale, S. *et al.* Polyploid evolution of the Brassicaceae during the Cenozoic era. *The Plant Cell* **26**, 2777–2791 (2014).
8. Huang, C.-H. *et al.* Resolution of Brassicaceae phylogeny using nuclear genes uncovers nested radiations and supports convergent morphological evolution. *Molecular Biology and Evolution* **33**, 394–412 (2016).
9. Koch, M. A. & Al-Shehbaz, I. A. Molecular systematics and evolution of "wild" crucifers (Brassicaceae or Cruciferae). in *Biology and Breeding of Crucifers* (ed. Gupta, S. K.) 1–19 (CRC Press, 2009).
10. Franzke, A., Lysak, M. A., Al-Shehbaz, I. A., Koch, M. A. & Mummenhoff, K. Cabbage family affairs: the evolutionary history of Brassicaceae. *Trends in Plant Science* **16**, 108–116 (2011).
11. Bailey, C. D. *et al.* Toward a global phylogeny of the Brassicaceae. *Molecular Biology and Evolution* **23**, 2142–2160 (2006).
12. Lysak, M. A., Koch, M. A., Beaulieu, J. M., Meister, A. & Leitch, I. J. The dynamic ups and downs of genome size evolution in Brassicaceae. *Molecular Biology and Evolution* **26**, 85–98 (2009).
13. Koch, M., Al-Shehbaz, I. A. & Mummenhoff, K. Molecular systematics, evolution, and population biology in the mustard family (Brassicaceae). *Annals of the Missouri Botanical Garden* **90**, 151–171 (2003).
14. Koch, M. A., German, D. A., Kiefer, M. & Franzke, A. Database taxonomics as key to modern plant biology. *Trends in Plant Science* **23**, 4–6 (2018).
15. Beilstein, M. A., Nagalingum, N. S., Clements, M. D., Manchester, S. R. & Mathews, S. Dated molecular phylogenies indicate a Miocene origin for *Arabidopsis thaliana*. *Proceedings of the National Academy of Sciences* **107**, 18724–18728 (2010).
16. Couvreur, T. L. P. *et al.* Molecular phylogenetics, temporal diversification, and principles of evolution in the mustard family (Brassicaceae). *Molecular Biology and Evolution* **27**, 55–71 (2010).
17. Franzke, A., German, D., Al-Shehbaz, I. A. & Mummenhoff, K. *Arabidopsis* family ties: molecular phylogeny and age estimates in Brassicaceae. *Taxon* **58**, 425–437 (2009).
18. Koch, M. A. *et al.* BrassiBase: Tools and biological resources to study characters and traits in the Brassicaceae—version 1.1. *Taxon* **61**, 1001–1009 (2012).
19. Mohammadin, S. *et al.* Anatolian origins and diversification of *Aethionema*, the sister lineage of the core Brassicaceae. *American Journal of Botany* **104**, 1042–1054 (2017).
20. Rešetnik, I., Satovic, Z., Schneeweiss, G. M. & Liber, Z. Phylogenetic relationships in Brassicaceae tribe Alysseae inferred from nuclear ribosomal and chloroplast DNA sequence data. *Molecular Phylogenetics and Evolution* **69**, 772–786 (2013).
21. Khosravi, A. R., Mohsenzadeh, S. & Mummenhoff, K. Phylogenetic relationships of old world Brassicaceae from Iran based on nuclear ribosomal DNA sequences. *Biochemical Systematics and Ecology* **37**, 106–115 (2009).
22. Alexander, P. J. *et al.* Molecular phylogenetics and taxonomy of the genus *Boechera* and related genera (Brassicaceae: Boechereae). *Systematic Botany* **38**, 192–209 (2013).
23. Arias, T. & Pires, C. J. A fully resolved chloroplast phylogeny of the brassica crops and wild relatives (Brassicaceae: Brassiceae): Novel clades and potential taxonomic implications. *Taxon* **61**, 980–988 (2012).
24. Clauss, M. J. & Koch, M. A. Poorly known relatives of *Arabidopsis thaliana*. *Trends in Plant Science* **11**, 449–459 (2006).
25. Carlsen, T., Bleeker, W., Hurka, H., Elven, R. & Brochmann, C. Biogeography and phylogeny of *Cardamine* (Brassicaceae). *Annals of the Missouri Botanical Garden* **96**, 215–236 (2009).
26. German, D. A., Grant, J. R., Lysak, M. A. & Al-Shehbaz, I. A. Molecular phylogeny and systematics of the tribe Chorisporeae (Brassicaceae). *Plant Systematics and Evolution* **294**, 65–86 (2011).
27. Koch, M. A. & German, D. A. Taxonomy and systematics are key to biological information: *Arabidopsis*, *Eutrema* (*Thellungiella*), *Noccaea* and *Schrenkiella* (Brassicaceae) as examples. *Frontiers in Plant Science* **4**, e267 (2013).
28. Ali, T. *et al.* Morphology, phylogeny, and taxonomy of *Microthlaspi* (Brassicaceae: Coluteocarpeae) and related genera. *Taxon* **65**, 79–98 (2016).
29. Goodson, B. E., Rehman, S. K. & Jansen, R. K. Molecular systematics and biogeography of *Descurainia* (Brassicaceae) based on nuclear ITS and non-coding chloroplast DNA. *Systematic Botany* **36**, 957–980 (2011).
30. Hao, G. *et al.* An integrative study of evolutionary diversification of *Eutrema* (Eutremeae, Brassicaceae). *Botanical Journal of the Linnean Society* **184**, 204–223 (2017).
31. Bailey, C. D., Al-Shehbaz, I. A. & Rajanikanth, G. Generic limits in tribe Halimolobeae and description of the new genus *Exhalimolobos* (Brassicaceae). *Systematic Botany* **32**, 140–156 (2007).
32. Mummenhoff, K., Al-Shehbaz, I. A., Bakker, F. T., Linder, H. P. & Mühlhausen, A. Phylogeny, morphological evolution, and speciation of endemic Brassicaceae genera in the Cape Flora of southern Africa. *Annals of the Missouri Botanical Garden* **92**, 400–424 (2005).
33. Moazzeni, H., Zarre, S., Al-Shehbaz, I. A. & Mummenhoff, K. Phylogeny of *Isatis* (Brassicaceae) and allied genera based on ITS sequences of nuclear ribosomal DNA and morphological characters. *Flora - Morphology, Distribution, Functional Ecology of Plants* **205**, 337–343 (2010).
34. Mummenhoff, K., Polster, A., Mühlhausen, A. & Theißen, G. *Lepidium* as a model system for studying the evolution of fruit development in Brassicaceae. *Journal of Experimental Botany* **60**, 1503–1513 (2009).
35. Heenan, P. B., Goeke, D. F., Houliston, G. J. & Lysak, M. A. Phylogenetic analyses of ITS and rbcL DNA sequences for sixteen genera of Australian and New Zealand Brassicaceae result in the expansion of the tribe Microlepidieae. *Taxon* **61**, 970–979 (2012).

36. Warwick, S. I., Sauder, C. A., Mayer, M. S. & Al-Shehbaz, I. A. Phylogenetic relationships in the tribes Schizopetaleae and Thelypodieae (Brassicaceae) based on nuclear ribosomal ITS region and plastid ndh F DNA sequences. *Botany* **87**, 961–985 (2009).
37. Bartish, I. V. *et al.* Phylogeny and colonization history of *Pringlea antiscorbutica* (Brassicaceae), an emblematic endemic from the South Indian Ocean Province. *Molecular Phylogenetics and Evolution* **65**, 748–756 (2012).
38. Ivalú Cacho, N., Millie Burrell, A., Pepper, A. E. & Strauss, S. Y. Novel nuclear markers inform the systematics and the evolution of serpentine use in *Streptanthus* and allies (Thelypodieae, Brassicaceae). *Molecular Phylogenetics and Evolution* **72**, 71–81 (2014).
39. Esmailbegi, S. *et al.* Phylogeny and systematics of the tribe Thlaspideae (Brassicaceae) and the recognition of two new genera. *Taxon* **67**, 324–340 (2018).
40. Koch, M. & Al-Shehbaz, I. A. Molecular systematics of the chinese *Yinshania* (Brassicaceae): evidence from plastid and nuclear ITS DNA sequence data. *Annals of the Missouri Botanical Garden* **87**, 246–272 (2000).
41. Koch, M. A. Mid-Miocene divergence of *Ionopsidium* and *Cochlearia* and its impact on the systematics and biogeography of the tribe Cochlearieae (Brassicaceae). *Taxon* **61**, 76–92 (2012).
42. Warwick, S. I., Sauder, C. A., Al-Shehbaz, I. A. & Jacquemoud, F. Phylogenetic relationships in the tribes Anchonieae, Chorisporeae, Euclidieae, and Hesperideae (Brassicaceae) based on nuclear ribosomal ITS DNA sequences. *Annals of the Missouri Botanical Garden* **94**, 56–78 (2007).
43. German, D. A., Friesen, N., Neuffer, B., Al-Shehbaz, I. A. & Hurka, H. Contribution to ITS phylogeny of the Brassicaceae, with special reference to some Asian taxa. *Plant Systematics and Evolution* **283**, 33–56 (2009).
44. Fuentes-Soriano, S. & Al-Shehbaz, I. Phylogenetic relationships of mustards with multiaperturate pollen (Physarieae, Brassicaceae) based on the plastid *ndhF* gene: implications for morphological diversification. *Systematic Botany* **38**, 178–191 (2013).
45. Karl, R. & Koch, M. A. A world-wide perspective on crucifer speciation and evolution: phylogenetics, biogeography and trait evolution in tribe Arabideae. *Annals of Botany* **112**, 983–1001 (2013).
46. Özüdoğru, B., Al-Shehbaz, I. A. & Mummenhoff, K. Tribal assignment of *Heldreichia* Boiss. (Brassicaceae): evidence from nuclear ITS and plastidic ndhF markers. *Plant Systematics and Evolution* **303**, 329–335 (2017).
47. Chen, H., Al-Shehbaz, I. A., Yue, J. & Sun, H. New insights into the taxonomy of tribe Euclidieae (Brassicaceae), evidence from nrITS sequence data. *PhytoKeys* **100**, 125–139 (2018).
48. Friesen, N. *et al.* Dated phylogenies and historical biogeography of *Dontostemon* and *Clausia* (Brassicaceae) mirror the palaeogeographical history of the Eurasian steppe. *Journal of Biogeography* **43**, 738–749 (2016).
49. Warwick, S. I., Al-Shehbaz, I. A., Sauder, C. A., Murray, D. F. & Mummenhoff, K. Phylogeny of *Smelowskia* and related genera (Brassicaceae) based on nuclear ITS DNA and chloroplast trnL intron DNA sequences. *Annals of the Missouri Botanical Garden* **91**, 99–123 (2004).
50. Salariato, D. L., Zuloaga, F. O., Franzke, A., Mummenhoff, K. & Al-Shehbaz, I. A. Diversification patterns in the CES clade (Brassicaceae tribes Cremolobeae, Eudemeae, Schizopetaleae) in Andean South America. *Botanical Journal of the Linnean Society* **181**, 543–566 (2016).
51. Warwick, S. I., Francis, A. & Al-Shehbaz, I. A. Brassicaceae: Species checklist and database on CD-Rom. *Plant Systematics and Evolution* **259**, 249–258 (2006).
52. Warwick, S. I., Mummenhoff, K., Sauder, C. A., Koch, M. A. & Al-Shehbaz, I. A. Closing the gaps: phylogenetic relationships in the Brassicaceae based on DNA sequence data of nuclear ribosomal ITS region. *Plant Systematics and Evolution* **285**, 209–232 (2010).
53. Koch, M. A., Dobeš, C. & Mitchell-Olds, T. Multiple hybrid formation in natural populations: Cconcerted Evolution of the Internal Transcribed Spacer of nuclear ribosomal DNA (ITS) in North American *Arabis divaricarpa* (Brassicaceae). *Molecular Biology and Evolution* **20**, 338–350 (2003).
54. Clarkson, J. J. *et al.* Phylogenetic relationships in *Nicotiana* (Solanaceae) inferred from multiple plastid DNA regions. *Molecular phylogenetics and evolution* **33**, 75–90 (2004).
55. Chase, M. W. *et al.* Molecular systematics, GISH and the origin of hybrid taxa in *Nicotiana* (Solanaceae). *Annals of Botany* **92**, 107–127 (2003).
56. Joly, S., Heenan, P. B. & Lockhart, P. J. A Pleistocene inter-tribal allopolyploidization event precedes the species radiation of *Pachycladon* (Brassicaceae) in New Zealand. *Molecular phylogenetics and evolution* **51**, 365–372 (2009).
57. Forsythe, E. S., Nelson, A. D. L. & Beilstein, M. A. *Biased gene retention in the face of massive nuclear introgression obscures species relationships*. http://biorxiv.org/lookup/doi/10.1101/197087 (2017) doi:10.1101/197087.
58. Zhang, Y.-H. A comparison of chromosome numbers and peroxidase zymograms of *Yinshania* and *Hilliella*. *Journal of plant resources and environment* 02 (1995).
59. Al-Shehbaz, I. A., German, D. A., Karl, R., Jordon-Thaden, I. & Koch, M. A. Nomenclatural adjustments in the tribe Arabideae (Brassicaceae). *Plant Diversity and Evolution* **129**, 71–76 (2011).
60. Koch, M. A., Karl, R., German, D. A. & Al-Shehbaz, I. A. Systematics, taxonomy and biogeography of three new Asian genera of Brassicaceae tribe Arabideae: an ancient distribution circle around the Asian high mountains. *Taxon* **61**, 955–969 (2012).
61. Warwick, S. I., Mummenhoff, K., Sauder, C. A., Koch, M. A. & Al-Shehbaz, I. A. Closing the gaps: phylogenetic relationships in the Brassicaceae based on DNA sequence data of nuclear ribosomal ITS region. *Plant Systematics and Evolution* **285**, 209–232 (2010).
62. German, D. A. & Al-Shehbaz, I. A. A reconsideration of *Pseudofortuynia* and *Tchihatchewia* as synonyms of *Sisymbrium* and *Hesperis*, respectively (Brassicaceae). *Phytotaxa* **334**, 95–98 (2018).
63. Kiefer, C. *et al.* Interspecies association mapping links reduced CG to TG substitution rates to the loss of gene-body methylation. *Nat. Plants* **5**, 846–855 (2019).
64. Hu, H. *et al.* Species delimitation in *Orychophragmus* (Brassicaceae) based on chloroplast and nuclear DNA barcodes. *Taxon* **64**, 714–726 (2015).
65. Toro-Núñez, O., Al-Shehbaz, I. A. & Mort, M. E. Phylogenetic study with nuclear and chloroplast data and ecological niche reveals *Atacama* (Brassicaceae), a new monotypic genus endemic from the Andes of the Atacama Desert, Chile. *Plant Systematics and Evolution* **301**, 1377–1396 (2015).
66. Salariato, D. L., Zuloaga, F. O., Franzke, A., Mummenhoff, K. & Al-Shehbaz, I. A. Diversification patterns in the CES clade (Brassicaceae tribes Cremolobeae, Eudemeae, Schizopetaleae) in Andean South America. *Botanical Journal of the Linnean Society* **181**, 543–566 (2016).

67. Al-Shehbaz, I. A., Beilstein, M. A. & Kellogg, E. A. Systematics and phylogeny of the Brassicaceae (Cruciferae): an overview. *Plant Systematics and Evolution* **259**, 89–120 (2006).
68. Mandáková, T., Li, Z., Barker, M. S. & Lysak, M. A. Diverse genome organization following 13 independent mesopolyploid events in Brassicaceae contrasts with convergent patterns of gene retention. *The Plant Journal* **91**, 3–21 (2017).
69. Salariato, D. L., Zuloaga, F. O., Cano, A. & Al-Shehbaz, I. A. Molecular phylogenetics of tribe Eudemeae (Brassicaceae) and implications for its morphology and distribution. *Molecular Phylogenetics and Evolution* **82**, 43–59 (2015).
70. Salariato, D. L., Zuloaga, F. O. & Al-Shehbaz, I. A. Molecular phylogeny of *Menonvillea* and recognition of the new genus *Aimara* (Brassicaceae: Cremolobeae). *Taxon* **62**, 1220–1234 (2013).
71. Huang, X.-C., German, D. A. & Koch, M. A. Temporal patterns of diversification in Brassicaceae demonstrate decoupling of rate shifts and mesopolyploidization events. *Annals of Botany* mcz123 (2019) doi:10.1093/aob/mcz123.
72. Karl, R. Phylogeny and systematics of the tribe Arabideae with a special focus on the genus *Arabis* L. *PhD thesis, University of Heidelberg* (2012).
73. As‘adī, M., Sajedi, S., Ranjberi, H. F. & Mu’assasa-i Taḥqīqāt-i Ğangalhā wa-Marātiʻ. *Flora of Iran: Brassicaceae / M. Assadi, S. Sajedi, H. Fakhr Ranjberi [und 14 andere]*. (2017).
74. Al-Shehbaz, I. A. A generic and tribal synopsis of the Brassicaceae (Cruciferae). *Taxon* **61**, 931–954 (2012).
75. Kiefer, M. *et al.* BrassiBase: Introduction to a novel knowledge database on Brassicaceae evolution. *Plant and Cell Physiology* **55**, e3 (2014).
76. Al-Shehbaz, I. A. A synopsis of the genus *Noccaea* (Coluteocarpeae, Brassicaceae). *Harvard Papers in Botany* **19**, 25–51 (2014).
77. Al-Shehbaz, I. A. *Chaunanthus torulosus* sp. nov. (Brassicaceae) from Puebla (Mexico) and a synopsis of the genus. *Nordic Journal of Botany* **32**, 133–136 (2014).
78. Al-Shehbaz, I. A. What is *Cochlearia conway* (Brassicaceae)? *Iran J Bot* **20**, 143–145 (2014).
79. Esmailbegi, S. *et al.* A taxonomic revision of the genus *Pseudocamelina* (Brassicaceae, tribe Thlaspideae). *Phytotaxa* **313**, 117–129 (2017).
80. Al-Shehbaz, I. A. *Bengt-jonsellia* (Brassicaceae), a new genus from Madagascar. *Harvard Papers in Botany* **22**, 1–3 (2017).
81. Al-Shehbaz, I. A. & German, D. A. A synopsis of the genus *Parrya* (Brassicaceae). *Kew Bulletin* **68**, 457–475 (2013).
82. Al-Shehbaz, I. A. & German, D. A. Three new genera in the tribe Euclidieae (Brassicaceae). *Novon: A Journal for Botanical Nomenclature* **25**, 12–17 (2016).
83. Al-Shehbaz, I. A., German, D. A., Mummenhoff, K. & Moazzeni, H. Systematics, tribal placements, and synopses of the *Malcolmia* S.L. segregates (Brassicaceae). *Harvard Papers in Botany* **19**, 53–71 (2014).
84. Al-Shehbaz, I. A., Gonzáles, P. & Cano, A. *Englerocharis blanca-leoniae* (Brassicaceae), a new species from Puno, Peru. *Harvard Papers in Botany* **20**, 1–4 (2015).
85. Al-Shehbaz, I. A., Gonzáles, P. & Cano, A. *Weberbauera incisa* (Brassicaceae), a new species from southern Peru. *Novon: A Journal for Botanical Nomenclature* **24**, 6–8 (2015).
86. Al-Shehbaz, I. A., Cano, A. & Trinidad, H. *Dactylocardamum polyspermum* (Brassicaceae), a second remarkable new species from Peru. *Novon: A Journal for Botanical Nomenclature* **25**, 259–262 (2017).
87. Brullo, C., Brullo, S., Galdo, G. G. D. & Ilardi, V. *Brassica trichocarpa* (Brassicaceae), a new species from Sicily. *Phytotaxa* **122**, 45–60 (2013).
88. Chen, H., Deng, T., Yue, J., Al-Shehbaz, I. A. & Sun, H. Molecular phylogeny reveals the non-monophyly of tribe Yinshanieae (Brassicaceae) and description of a new tribe, Hillielleae. *Plant Diversity* **38**, 171–182 (2016).
89. Zhang, Y.-H. Delimitation and revision of *Hilliella* and *Yinshania* (Brassicaceae). *Acta Phytotaxonomica Sinica* **41**, 205–349 (2003).
90. German, D. A. & Friesen, N. W. *Shehbazia* (Shehbazieae, Cruciferae), a new monotypic genus and tribe of hybrid origin from Tibet. *Turczaninowia* **17**, 17–23 (2014).
91. Heenan, P. B., Molloy, B. P. J. & Smissen, R. D. *Cardamine cubita* (Brassicaceae), a new species from New Zealand with a remarkable reduction in floral parts. *Phytotaxa* **140**, 43–50 (2013).
92. Hildebrand, T. J. & Al-Shehbaz, I. A. *Terraria haydenii* (Thelypodieae, Brassicaceae), a new mustard genus and species from the West Desert region of North America’s Great Basin. *Phytotaxa* **323**, 128–142 (2017).
93. Salariato, D. L. & Al-Shehbaz, I. A. <I>Zuloagocardamum</I> (Brassicaceae: Thelypodieae) a new genus from the Andes highlands of northern Argentina. *Systematic Botany* **39**, 563–577 (2014).
94. Salariato, D. L. & Zuloaga, F. O. Taxonomic placement of *Onuris hauthalii* (Brassicaceae: Eudemeae), based on morphology and multilocus species tree analyses, and the recognition of the new genus Alshehbazia. *Kew Bulletin* **70**, (2015).
95. Salariato, D. L., Zuloaga, F. O. & Al-Shehbaz, I. A. A revision of the genus *Menonvillea* (Cremolobeae, Brassicaceae). *Phytotaxa* **162**, 241–2989 (2014).
96. Španiel, S. *et al.* AlyBase: database of names, chromosome numbers, and ploidy levels of Alysseae (Brassicaceae), with a new generic concept of the tribe. *Plant Systematics and Evolution* **301**, 2463–2491 (2015).
97. Yüzbaşioğlu, S., Al-Shehbaz, I. A., Yüzbaşioğlu, E. & Dalyan, E. *Berteroa physocarpa* (Brassicaceae), a new species from NW Turkey based on morphological and molecular data. *Phytotaxa* **305**, 87–96 (2017).
98. Zhou, Y.-Y., Zhang, H.-W., Hu, J.-Q. & Jin, X.-F. *Sinalliaria*, a new genus of Brassicaceae from eastern China, based on morphological and molecular data. *Phytotaxa* **186**, 188–198 (2014).
99. Ziffer-Berger, J., Hanin, N., Fogel, T., Mummenhoff, K. & Barazani, O. Molecular phylogeny indicates polyphyly in *Raphanus* L. (Brassicaceae). *Edinburgh Journal of Botany* **72**, 1–11 (2015).
100. Al-Shehbaz, I. A. *Brassicaceae: Volume 8. Flora of Argentina*. vol. 8 (Instituto de Botánica Darwinion; Instituto Multidisciplinario de Biología Vegetal, 2012).
101. Al-Shehbaz, I. A. *Brassicaceae: Volume 30. Flora of the Pan-Himalaya*. vol. 30 (Cambridge University Press, 2016).
102. Schulz, O. E. Cruciferae. in *Die natürlichen Pflanzenfamilien* (eds. Engler, A. & Harms, H.) vol. 17B 227–658 (Englemann, 1936).
103. von Hayek, A. Entwurf eines Cruciferen-systems auf phylogenetischer Grundlage. *Beihefte Bot Centralblatt* **27**, 127–335 (1911).
104. Janchen, E. Das System der Cruciferen. *Österr. Bot. Zeit* **91**, 1–19 (1942).
105. Appel, O. & Al-Shehbaz, I. A. Cruciferae. in *Flowering Plants · Dicotyledons: Malvales, Capparales and Non-betalain Caryophyllales* (eds. Kubitzki, K. & Bayer, C.) 75–174 (Springer Berlin Heidelberg, 2003). doi:10.1007/978-3-662-07255-4_17.

106. Beilstein, M. A., Al-Shehbaz, I. A. & Kellogg, E. A. Brassicaceae phylogeny and trichome evolution. *Am. J. Bot.* **93**, 607–619 (2006).
107. Fér, T. & Schmickl, R. E. HybPhyloMaker: Target enrichment data analysis from raw reads to species trees. *Evolutionary Bioinformatics* **14**, 1176934317742613 (2018).
108. Bolger, A. M., Lohse, M. & Usadel, B. Trimmomatic: a flexible trimmer for Illumina sequence data. *Bioinformatics* **30**, 2114–2120 (2014).
109. Xu, H. *et al.* FastUniq: a fast de novo duplicates removal tool for paired short reads. *PloS one* **7**, (2012).
110. Langmead, B. & Salzberg, S. L. Fast gapped-read alignment with Bowtie 2. *Nature methods* **9**, 357 (2012).
111. Constantinides, B. & Robertson, D. Kindel: indel-aware consensus for nucleotide sequence alignments. *Journal of Open Source Software* **2**, 282 (2017).
112. Kent, W. J. BLAT—the BLAST-like alignment tool. *Genome research* **12**, 656–664 (2002).
113. Weitemier, K. *et al.* Hyb-Seq: Combining target enrichment and genome skimming for plant phylogenomics. *Applications in Plant Sciences* **2**, 1400042 (2014).
114. Katoh, K. & Standley, D. M. MAFFT multiple sequence alignment software version 7: improvements in performance and usability. *Molecular Biology and Evolution* **30**, 772–780 (2013).
115. Borowiec, M. L. AMAS: a fast tool for alignment manipulation and computing of summary statistics. *PeerJ* **4**, e1660 (2016).
116. Darriba, D. *et al.* ModelTest-NG: a new and scalable tool for the selection of DNA and protein evolutionary models. *Molecular biology and evolution* **37**, 291–294 (2020).
117. Kozlov, A. M., Darriba, D., Flouri, T., Morel, B. & Stamatakis, A. RAxML-NG: a fast, scalable and user-friendly tool for maximum likelihood phylogenetic inference. *Bioinformatics* **35**, 4453–4455 (2019).
118. Pattengale, N. D., Alipour, M., Bininda-Emonds, O. R., Moret, B. M. & Stamatakis, A. How many bootstrap replicates are necessary? *Journal of computational biology* **17**, 337–354 (2010).
119. Schliep, K. P. phangorn: phylogenetic analysis in R. *Bioinformatics* **27**, 592–593 (2011).
120. Huson, D. H. *et al.* Dendroscope: An interactive viewer for large phylogenetic trees. *BMC Bioinformatics* **8**, 460 (2007).
121. Drummond, A. J., Suchard, M. A., Xie, D. & Rambaut, A. Bayesian phylogenetics with BEAUti and the BEAST 1.7. *Molecular Biology and Evolution* **29**, 1969–1973 (2012).
122. Mandáková, T., Hloušková, P., German, D. A. & Lysak, M. A. Monophyletic origin and evolution of the largest crucifer genomes. *Plant Physiology* **174**, 2062–2071 (2017).
123. Gittleman, J. L. & Kot, M. Adaptation: Statistics and a null model for estimating phylogenetic effects. *Systematic Biology* **39**, 227–241 (1990).
124. Felsenstein, J. Phylogenies and the comparative method. *The American Naturalist* **125**, 1–15 (1985).
125. Garland, T., Janis, C. M. & Jones, J. A. Phylogenetic analysis of covariance by computer simulation. *Systematic Biology* **42**, 28 (1993).
126. Benedict, J. C., DeVore, M. L. & Pigg, K. B. *Prunus* and *Oemleria* (Rosaceae) flowers from the late early Eocene republic flora of Northeastern Washington State, U.S.A. *International Journal of Plant Sciences* **172**, 948–958 (2011).
127. Njuguna, W., Liston, A., Cronn, R., Ashman, T.-L. & Bassil, N. Insights into phylogeny, sex function and age of *Fragaria* based on whole chloroplast genome sequencing. *Molecular Phylogenetics and Evolution* **66**, 17–29 (2013).
128. Sims, H. J., Herendeen, P. S., Lupia, R., Christopher, R. A. & Crane, P. R. Fossil flowers with Normapolles pollen from the Upper Cretaceous of southeastern North America. *Review of Palaeobotany and Palynology* **106**, 131–151 (1999).
129. Moore, M. J., Soltis, P. S., Bell, C. D., Burleigh, J. G. & Soltis, D. E. Phylogenetic analysis of 83 plastid genes further resolves the early diversification of eudicots. *Proceedings of the National Academy of Sciences* **107**, 4623–4628 (2010).
130. Knobloch, E. & Mai, D. Monograph of the fruits and seeds in the Cretaceous of Central Europe. *Rozpravy Ústreõdního Ústavu Geologickéh* **47**, 1–219 (1986).
131. Bell, C. D., Soltis, D. E. & Soltis, P. S. The age and diversification of the angiosperms re-revisited. *American Journal of Botany* **97**, 1296–1303 (2010).
132. Takahashi, M., Crane, P. R. & Ando, H. Fossil flowers and associated plant fossils from the Kamikitaba locality (Ashizawa formation, Futaba group, Lower Coniacian, Upper Cretaceous) of Northeast Japan. *Journal of Plant Research* **112**, 187–206 (1999).
133. Magallón, S., Gómez-Acevedo, S., Sánchez-Reyes, L. L. & Hernández-Hernández, T. A metacalibrated time-tree documents the early rise of flowering plant phylogenetic diversity. *New Phytologist* **207**, 437–453 (2015).
