# Supplementary material for: Nested whole-genome duplications coincide with diversification and high morphological disparity in Brassicaceae
Source: Nat Commun. 2020 Jul 30;11:3795. doi: 10.1038/s41467-020-17605-7 (PMC7393125; doi:10.1038/s41467-020-17605-7)
Supplement: Supplementary file 3 — Reporting Summary [file 41467_2020_17605_MOESM3_ESM.pdf]

nature research

Corresponding author(s): Marcus A. Koch

Last updated by author(s): 06/26/2020

Reporting Summary

Nature Research wishes to improve the reproducibility of the work that we publish. This form provides structure for consistency and transparency in reporting. For further information on Nature Research policies, see Authors & Referees and the Editorial Policy Checklist .

Statistics

For all statistical analyses, confirm that the following items are present in the figure legend, table legend, main text, or Methods section.

n/a	Confirmed	
☐	☒	The exact sample size (*n*) for each experimental group/condition, given as a discrete number and unit of measurement
☒	☐	A statement on whether measurements were taken from distinct samples or whether the same sample was measured repeatedly
☐	☒	The statistical test(s) used AND whether they are one- or two-sided<br>*Only common tests should be described solely by name; describe more complex techniques in the Methods section.*
☐	☒	A description of all covariates tested
☐	☒	A description of any assumptions or corrections, such as tests of normality and adjustment for multiple comparisons
☐	☒	A full description of the statistical parameters including central tendency (e.g. means) or other basic estimates (e.g. regression coefficient) AND variation (e.g. standard deviation) or associated estimates of uncertainty (e.g. confidence intervals)
☐	☒	For null hypothesis testing, the test statistic (e.g. *F*, *t*, *r*) with confidence intervals, effect sizes, degrees of freedom and *P* value noted<br>*Give P values as exact values whenever suitable.*
☐	☒	For Bayesian analysis, information on the choice of priors and Markov chain Monte Carlo settings
☒	☐	For hierarchical and complex designs, identification of the appropriate level for tests and full reporting of outcomes
☐	☒	Estimates of effect sizes (e.g. Cohen's *d*, Pearson's *r*), indicating how they were calculated

Our web collection on statistics for biologists contains articles on many of the points above.

Software and code

Policy information about availability of computer code

Data collection	No software was used
Data analysis	The following software was used: R 3.5.2, factoMineR 1.41, NUCLEAR 3.2.4, VISION 2.6.12, Geneious 7.1.7, MAFFT 7.017, Gblocks 0.91b, PartitionFinder 2.1.1, RAxML 8.1.16, ape 3.1-4, BEAST 1.8.4, LogCombiner 2.4.7, TreeAnnotator 2.4.7, FigTree 1.4.1, phylosignal 1.2.1, BAMM 2.5.0, BAMMtools 2.1.6, coda 0.19-2, adegenet 2.0.1, phytools 0.6-60, HybPhyloMaker, Trimmomatic 0.32, FastUniq 1.1, Bowtie 2 2.2.4, Kindel 0.1.4, BLAT 32×1, MAFFT 7.029, AMAS 0.98, ModelTest-NG 0.1.6, RAxML-NG 0.8, phangorn 2.5.5, dendroscope 3.7.2

For manuscripts utilizing custom algorithms or software that are central to the research but not yet described in published literature, software must be made available to editors/reviewers. We strongly encourage code deposition in a community repository (e.g. GitHub). See the Nature Research guidelines for submitting code & software for further information.

Data

Policy information about availability of data

All manuscripts must include a data availability statement. This statement should provide the following information, where applicable:

- Accession codes, unique identifiers, or web links for publicly available datasets
- A list of figures that have associated raw data
- A description of any restrictions on data availability

The plastome sequences generated during the current study are available at ENA/GenBank under accession codes MK637648-MK637830 (see Supplementary Table S14), raw sequencing data is available under ENA/GenBank bioproject PRJEB38700 [https://www.ncbi.nlm.nih.gov/bioproject/PRJEB38700]. The taxonomic and morphological data sets as well as alignments and the dated phylogeny are available at Dryad [https://doi.org/10.5061/dryad.fttdz08pt]. The species checklist is also available at BrassiBase [https://brassibase.cos.uni-heidelberg.de/].
Furthermore, the following data was obtained from databases:
NC_009265 [https://www.ncbi.nlm.nih.gov/nuccore/NC_009265]; NC_009266 [https://www.ncbi.nlm.nih.gov/nuccore/NC_009266]; NC_034367 [https://www.ncbi.nlm.nih.gov/nuccore/NC_034367]; NC_034299.1 [https://www.ncbi.nlm.nih.gov/nuccore/NC_034299.1]; MF169880.1 [https://www.ncbi.nlm.nih.gov/

nuccore/MF169880.1]; NC_009267 [https://www.ncbi.nlm.nih.gov/nuccore/NC_009267]; NC_009274 [https://www.ncbi.nlm.nih.gov/nuccore/NC_009274]; KY912021.1 [https://www.ncbi.nlm.nih.gov/nuccore/KY912021.1]; KY912025.1 [https://www.ncbi.nlm.nih.gov/nuccore/KY912025.1]; NC_035514.1 [https://www.ncbi.nlm.nih.gov/nuccore/NC_035514.1]; NC_035513.1 [https://www.ncbi.nlm.nih.gov/nuccore/NC_035513.1]; KY912030.1 [https://www.ncbi.nlm.nih.gov/nuccore/KY912030.1]; HF934132 [https://www.ncbi.nlm.nih.gov/nuccore/HF934132]; NC_009268 [https://www.ncbi.nlm.nih.gov/nuccore/NC_009268]; NC_009272 [https://www.ncbi.nlm.nih.gov/nuccore/NC_009272]; KY126841.1 [https://www.ncbi.nlm.nih.gov/nuccore/KY126841.1]; NC_016734 [https://www.ncbi.nlm.nih.gov/nuccore/NC_016734]; KJ716483 [https://www.ncbi.nlm.nih.gov/nuccore/KJ716483]; NC_030775.1 [https://www.ncbi.nlm.nih.gov/nuccore/NC_030775.1]; NC_033499.1 [https://www.ncbi.nlm.nih.gov/nuccore/NC_033499.1]; NC_033500.1 [https://www.ncbi.nlm.nih.gov/nuccore/NC_033500.1]; NC_033498.1 [https://www.ncbi.nlm.nih.gov/nuccore/NC_033498.1]; NC_015139.1 [https://www.ncbi.nlm.nih.gov/nuccore/NC_015139.1]; NC_030450.1 [https://www.ncbi.nlm.nih.gov/nuccore/NC_030450.1]; NC_028272.1 [https://www.ncbi.nlm.nih.gov/nuccore/NC_028272.1]; NC_034287.1 [https://www.ncbi.nlm.nih.gov/nuccore/NC_034287.1]; NC_034286.1 [https://www.ncbi.nlm.nih.gov/nuccore/NC_034286.1]; NC_035303.1 [https://www.ncbi.nlm.nih.gov/nuccore/NC_035303.1]; LN877377 [https://www.ncbi.nlm.nih.gov/nuccore/LN877377]; LN877376 [https://www.ncbi.nlm.nih.gov/nuccore/LN877376]; LN877378 [https://www.ncbi.nlm.nih.gov/nuccore/LN877378]; LN877380 [https://www.ncbi.nlm.nih.gov/nuccore/LN877380]; LN877381 [https://www.ncbi.nlm.nih.gov/nuccore/LN877381]; LN877382 [https://www.ncbi.nlm.nih.gov/nuccore/LN877382]; LN877383 [https://www.ncbi.nlm.nih.gov/nuccore/LN877383]; LN877384 [https://www.ncbi.nlm.nih.gov/nuccore/LN877384]; AP000423 [https://www.ncbi.nlm.nih.gov/nuccore/AP000423]; LN877386 [https://www.ncbi.nlm.nih.gov/nuccore/LN877386]; NC_009270 [https://www.ncbi.nlm.nih.gov/nuccore/NC_009270]; LN877385 [https://www.ncbi.nlm.nih.gov/nuccore/LN877385]; NC_028517.1 [https://www.ncbi.nlm.nih.gov/nuccore/NC_028517.1]; NC_009269 [https://www.ncbi.nlm.nih.gov/nuccore/NC_009269]; NC_009275 [https://www.ncbi.nlm.nih.gov/nuccore/NC_009275]; NC_026445 [https://www.ncbi.nlm.nih.gov/nuccore/NC_026445]; NC_026446 [https://www.ncbi.nlm.nih.gov/nuccore/NC_026446]; LN866844 [https://www.ncbi.nlm.nih.gov/nuccore/LN866844]; LN866845 [https://www.ncbi.nlm.nih.gov/nuccore/LN866845]; LN866846 [https://www.ncbi.nlm.nih.gov/nuccore/LN866846]; LN866847 [https://www.ncbi.nlm.nih.gov/nuccore/LN866847]; LN866848 [https://www.ncbi.nlm.nih.gov/nuccore/LN866848]; LT629930 [https://www.ncbi.nlm.nih.gov/nuccore/LT629930]; NC_009271 [https://www.ncbi.nlm.nih.gov/nuccore/NC_009271]; LN877375 [https://www.ncbi.nlm.nih.gov/nuccore/LN877375]; NC_035515.1 [https://www.ncbi.nlm.nih.gov/nuccore/NC_035515.1]; KY912024.1 [https://www.ncbi.nlm.nih.gov/nuccore/KY912024.1]; NC_034361.1 [https://www.ncbi.nlm.nih.gov/nuccore/NC_034361.1]; NC_034359.1 [https://www.ncbi.nlm.nih.gov/nuccore/NC_034359.1]; KT962846 [https://www.ncbi.nlm.nih.gov/nuccore/KT962846]; KT962847 [https://www.ncbi.nlm.nih.gov/nuccore/KT962847]; NC_028728 [https://www.ncbi.nlm.nih.gov/nuccore/NC_028728]; NC_028727 [https://www.ncbi.nlm.nih.gov/nuccore/NC_028727]; LN877374 [https://www.ncbi.nlm.nih.gov/nuccore/LN877374]; NC_035512.1 [https://www.ncbi.nlm.nih.gov/nuccore/NC_035512.1]; NC_009273 [https://www.ncbi.nlm.nih.gov/nuccore/NC_009273]; NC_034363.1 [https://www.ncbi.nlm.nih.gov/nuccore/NC_034363.1]; NC_034360.1 [https://www.ncbi.nlm.nih.gov/nuccore/NC_034360.1]; NC_030516.1 [https://www.ncbi.nlm.nih.gov/nuccore/NC_030516.1]; NC_030515.1 [https://www.ncbi.nlm.nih.gov/nuccore/NC_030515.1]; JQ806762 [https://www.ncbi.nlm.nih.gov/nuccore/JQ806762]; JX205495 [https://www.ncbi.nlm.nih.gov/nuccore/JX205495]; KU170142.1 [https://www.ncbi.nlm.nih.gov/nuccore/KU170142.1]; NC_028726.1 [https://www.ncbi.nlm.nih.gov/nuccore/NC_028726.1]; EU431223 [https://www.ncbi.nlm.nih.gov/nuccore/EU431223]; HQ901196 [https://www.ncbi.nlm.nih.gov/nuccore/HQ901196]; HQ336404 [https://www.ncbi.nlm.nih.gov/nuccore/HQ336404]; KF986530 [https://www.ncbi.nlm.nih.gov/nuccore/KF986530]; DQ864733 [https://www.ncbi.nlm.nih.gov/nuccore/DQ864733]; EF205595 [https://www.ncbi.nlm.nih.gov/nuccore/EF205595]; KF753631 [https://www.ncbi.nlm.nih.gov/nuccore/KF753631]; KC180800 [https://www.ncbi.nlm.nih.gov/nuccore/KC180800]; HM347959 [https://www.ncbi.nlm.nih.gov/nuccore/HM347959]; KC180807 [https://www.ncbi.nlm.nih.gov/nuccore/KC180807]; GQ870669 [https://www.ncbi.nlm.nih.gov/nuccore/GQ870669]; EU262889 [https://www.ncbi.nlm.nih.gov/nuccore/EU262889]; HQ713469 [https://www.ncbi.nlm.nih.gov/nuccore/HQ713469]; JQ031013 [https://www.ncbi.nlm.nih.gov/nuccore/JQ031013]; HM125537 [https://www.ncbi.nlm.nih.gov/nuccore/HM125537]; KF240617 [https://www.ncbi.nlm.nih.gov/nuccore/KF240617]; KF240615 [https://www.ncbi.nlm.nih.gov/nuccore/KF240615]; MK434916 [https://www.ncbi.nlm.nih.gov/nuccore/MK434916]; AP012207 [https://www.ncbi.nlm.nih.gov/nuccore/AP012207]; KC571835 [https://www.ncbi.nlm.nih.gov/nuccore/KC571835]; HQ336405 [https://www.ncbi.nlm.nih.gov/nuccore/HQ336405]; JF345175 [https://www.ncbi.nlm.nih.gov/nuccore/JF345175]; HG931056 [https://www.ncbi.nlm.nih.gov/nuccore/HG931056]; KF753637 [https://www.ncbi.nlm.nih.gov/nuccore/KF753637]; DQ226511 [https://www.ncbi.nlm.nih.gov/nuccore/DQ226511]; KJ001129 [https://www.ncbi.nlm.nih.gov/nuccore/KJ001129]; HQ336406 [https://www.ncbi.nlm.nih.gov/nuccore/HQ336406]; JX970937 [https://www.ncbi.nlm.nih.gov/nuccore/JX970937]; DQ119058 [https://www.ncbi.nlm.nih.gov/nuccore/DQ119058]; HM029370 [https://www.ncbi.nlm.nih.gov/nuccore/HM029370]; KF042344 [https://www.ncbi.nlm.nih.gov/nuccore/KF042344]; KJ476730 [https://www.ncbi.nlm.nih.gov/nuccore/KJ476730]; KF241982 [https://www.ncbi.nlm.nih.gov/nuccore/KF241982]; EU835853 [https://www.ncbi.nlm.nih.gov/nuccore/EU835853]; AP002983 [https://www.ncbi.nlm.nih.gov/nuccore/AP002983]; EU196765 [https://www.ncbi.nlm.nih.gov/nuccore/EU196765]; DQ317523 [https://www.ncbi.nlm.nih.gov/nuccore/DQ317523]; NC_016708 [https://www.ncbi.nlm.nih.gov/nuccore/NC_016708]; KC695666 [https://www.ncbi.nlm.nih.gov/nuccore/KC695666]; NC_024057 [https://www.ncbi.nlm.nih.gov/nuccore/NC_024057]; HQ285842 [https://www.ncbi.nlm.nih.gov/nuccore/HQ285842]; EU117376 [https://www.ncbi.nlm.nih.gov/nuccore/EU117376]; JF937588 [https://www.ncbi.nlm.nih.gov/nuccore/JF937588]; KJ414485 [https://www.ncbi.nlm.nih.gov/nuccore/KJ414485]; KJ414486 [https://www.ncbi.nlm.nih.gov/nuccore/KJ414486]; EF489041 [https://www.ncbi.nlm.nih.gov/nuccore/EF489041]; KJ742926 [https://www.ncbi.nlm.nih.gov/nuccore/KJ742926]; NC_007957 [https://www.ncbi.nlm.nih.gov/nuccore/NC_007957]; doi:10.5061/dryad.kc5b8 [https://doi.org/10.5061/dryad.kc5b8]; doi:10.5061/dryad.kc5b8 [https://doi.org/10.5061/dryad.kc5b8]; doi:10.5061/dryad.kc5b8 [https://doi.org/10.5061/dryad.kc5b8]; PRJNA283303 [https://www.ncbi.nlm.nih.gov/bioproject/PRJNA283303]; doi:10.5061/dryad.kc5b8 [https://doi.org/10.5061/dryad.kc5b8]; doi:10.5061/dryad.kc5b8 [https://doi.org/10.5061/dryad.kc5b8]; doi:10.5061/dryad.kc5b8 [https://doi.org/10.5061/dryad.kc5b8]; doi:10.5061/dryad.kc5b8 [https://doi.org/10.5061/dryad.kc5b8]; doi:10.5061/dryad.kc5b8 [https://doi.org/10.5061/dryad.kc5b8]; doi:10.5061/dryad.kc5b8 [https://doi.org/10.5061/dryad.kc5b8]; doi:10.5061/dryad.kc5b8 [https://doi.org/10.5061/dryad.kc5b8]; doi:10.5061/dryad.kc5b8 [https://doi.org/10.5061/dryad.kc5b8]; doi:10.5061/dryad.kc5b8 [https://doi.org/10.5061/dryad.kc5b8]

Field-specific reporting

Please select the one below that is the best fit for your research. If you are not sure, read the appropriate sections before making your selection.

☐ Life sciences ☐ Behavioural & social sciences ☒ Ecological, evolutionary & environmental sciences

For a reference copy of the document with all sections, see nature.com/documents/nr-reporting-summary-flat.pdf

Ecological, evolutionary & environmental sciences study design

All studies must disclose on these points even when the disclosure is negative.

Study description	We reconstructed a phylogeny for Brassicaceae, using outgroup species from the Rosids. Divergence time was also estimated using this data set. Morphological data for 37 characters with two to five discrete character states was scored for all 351 genera of Brassicaceae, and lineage differences were assessed. Morphological disparity was calculated on genus and tribal (monophyletic clades of varying number of genera) level, and contribution of individual characters to distinguish lineages was analyzed. Association with genome sizes, taxonomic richness, stem/crown group age of tribes was estimated. Differences of these values between tribes

	having undergone tribe specific rate shifts or mesopolyploidization events, as well as differences in diversification rate were evaluated. Differences of tribal values between lineages were also assessed.<br>Sample sizes for all but plastome analyses were determined by the number of tribes (51) and genera (351) - complete sampling of all Brassicaceae species. As a result, a complete species checklist of almost 4000 species is released with this contribution.
Research sample	We assembled plastomes for 178 samples from 176 Brassicaceae species for phylogenetic reconstruction, as well as five species from different Brassicales families, aiming to cover the deepest phylogenetic nodes within all tribes of Brassicaceae, including existing data (see Data Availability section).<br>For morphological characters, data for all species of Brassicaceae was assembled on genus level.
Sampling strategy	Sampling for plastid phylogeny aimed to represent all tribes, and within tribes the oldest node.<br>For morphological characters, data for all species of Brassicaceae was assembled on genus level.
Data collection	D.A.G. and M.A.K. collected morphological data from 2011 onwards; herbaria throughout Europe and Asia, e.g. ALTB, B, BM, E, G, GAT, H, HBG, HEID, JE, LE, LI, M, MW, OSBU, P, W, WU were visited and literature on species and genus descriptions was consulted. E.W. extracted DNA for next generation sequencing in early 2016. Morphological data was collected manually through inspection by eye and using a microscope/binocular, if appropriate. Data on family-wide diversification rates were obtained from literature (Huang et al. 2019).
Timing and spatial scale	Samples for plastid phylogeny were obtained from herbarium vouchers, and included material collected worldwide.<br>Morphological variation was scored from literature and original voucher material, and thereby is based on data collected during the past two centuries. Morphological data over the entire Brassicaceae family was scored from 2012 until end of 2018 (see Data collection). Sequence data for phylogenetic analyses were generated from 2016 to 2018.
Data exclusions	No data was excluded from the analyses.
Reproducibility	Not applicable. Original vouchers from which DNA was extracted are documented and accessible through the respective herbaria.
Randomization	Not applicable. No randomization in this manuscript as samples were not allocated into experimental groups.
Blinding	Not applicable. Blinding was not relevant to this study, which focused on plant family-wide phylogenetic data and diversity patterns. Data were not allocated into groups.

Did the study involve field work? ☐ Yes ☒ No

Reporting for specific materials, systems and methods

We require information from authors about some types of materials, experimental systems and methods used in many studies. Here, indicate whether each material, system or method listed is relevant to your study. If you are not sure if a list item applies to your research, read the appropriate section before selecting a response.

Materials & experimental systems

n/a	Involved in the study
☒	☐ Antibodies
☒	☐ Eukaryotic cell lines
☒	☐ Palaeontology
☒	☐ Animals and other organisms
☒	☐ Human research participants
☒	☐ Clinical data

Methods

n/a	Involved in the study
☒	☐ ChIP-seq
☒	☐ Flow cytometry
☒	☐ MRI-based neuroimaging
