# Supplementary material for: Nested whole-genome duplications coincide with diversification and high morphological disparity in Brassicaceae
Source: Nat Commun. 2020 Jul 30;11:3795. doi: 10.1038/s41467-020-17605-7 (PMC7393125; doi:10.1038/s41467-020-17605-7)
Supplement: Supplementary file 4 — Description of Additional Supplementary Files [file 41467_2020_17605_MOESM4_ESM.pdf]

Description of Additional Supplementary Files

File name: Supplementary Data 1

Description: detailed accession table with all respective information
