# Supplementary material for: Nested whole-genome duplications coincide with diversification and high morphological disparity in Brassicaceae
Source: Nat Commun. 2020 Jul 30;11:3795. doi: 10.1038/s41467-020-17605-7 (PMC7393125; doi:10.1038/s41467-020-17605-7)
Supplement: Supplementary file 2 — Peer Review [file 41467_2020_17605_MOESM2_ESM.pdf]

Reviewers' comments:

Reviewer #1 (Remarks to the Author):

This an interesting and unique attempt to evaluate morphological disparity across the mustard family (Brassicaceae) using a family-wide plastome phylogeny. The phylogeny is the most comprehensive plastome phylogeny published for the crucifers. I also highly evaluate the attempt to evaluate the evolutionary significance of whole-genome duplications for speciation rate shifts and net diversification rates across the Brassicaceae.I have only a few minor comments summarized in my review attached.

Reviewer #2 (Remarks to the Author):

Review of "Nested whole-genome duplication coincide with diversification and high morphological disparity over geological times in the Brassicaceae"

This work by Walden et al presents a detailed phylogenetic analysis of the genus and tribes across Brassicaceae, addressing the morphological space realized by diversifying taxa with a focus on the role of whole genome duplication (WGD). This is a comprehensive and timely contribution to the field of plant evolution, bringing fresh insights on current debates regarding interactions between WGDs and diversification. The amount of data here integrated within a fine phylogenetic network is impressive and paves the way towards future work on forms and function in this model family for evolutionary studies.
The manuscript is well written, with detailed descriptions yielding confidence in sound data and approaches. Although some paragraphs may be streamlined to improve the impact and avoid unnecessary redundancy (e.g. between results and methods), the presentation is convincing and likely to raise interest among plant scientists and evolutionary biologists.
Here below, I will expend on a few issues that would benefit from further clarifications and that may strengthen the links between data and their interpretations.

Detailed comments
1. The end of the abstract is frustrating as it summarizes insights such as gain and loss of characters and genome modularity that are mostly discussed as perspectives rather than part of core results presented here. Such perspectives would be better discussed as such in the main text (also see comment 7), while not being mentioned in the abstract.
2. The introduction starts by introducing the process of WGD and then discusses 'polyploidy'. As the later concept also encompasses reticulate evolution (i.e. allopolyploidy) and given that this work is focused on maternal relationships based on plastid loci (i.e. cannot identify reticulations), some effort should be brought here (L.64) and throughout the manuscript to unify the terminology and make sure that readers are not being confused.
3. Although I acknowledge that the authors here loyally summarize (L. 79ff) the 'WGD radiation lag-time model' as originally presented, the ground for such a chain of event remains unclear. In particular, following e.g. Wagner (2017, The White-Knight hypothesis, or does the environment limit innovations?, Tr EcolEvol 32:131–140), why should 'key traits or innovations' be expected to evolve early and not later (e.g. at dispersal/radiation stages)? While the model here under scrutiny subordinates evolutionary radiation to shifts in distribution ranges and (possibly) environmental changes, this study does not address such factors and it remains unclear to what extent present results support or reject this model. I would recommend to clarify the background, and maybe use this 'lag-time model' only as a metaphor to enlighten results from L. 410 on.
4. Related to the previous comment, factors underlying 'disparity' should ideally be teased apart. WGD supporting reproductive isolation, it may promote the accumulation of morphological differences (e.g. L. 55) and thus support diversification as here evaluated (e.g. L. 283 and L. 293). The combination of factors driving disparity should be further quantified and discussed, and possibly integrated with views of a 'lag-time model' following WGD; at least verbally (e.g. L. 410).

5. Available insights regrading mesopolyploidy events should be further introduced and discussed. In particular, it remains unclear to what extent such events discovered in 11 tribes (L. 105) are representative of mesopolyploidy across Brassicaceae. Were the 40 remaining tribes demonstrated as lacking WGD events? Corresponding clarifications should be brought in as to support unbiased interpretations L. 170 (incl. Fig. 1) as well as specific contrasts based on 'WGD vs no WGD' (L. 292ff). On top of justifications, Fig. 3 should present sample sizes and map WGD events on the phylogenetic tree. When it comes to corresponding issues in the discussion (L. 397ff), it would indeed be great to know more about those 129 genera 'affected by additional mesopolyploidization' as well as the ca. 222 remaining. Similar justification may help to support the particularly high WGD rate exhibited by Brassicaceae.
6. L. 175ff: Correlation between crown group ages here inferred and available from prior studies based on nuclear loci is based on only 24 data points. As such dating is associated with uncertainty, it remains unclear how estimates of 'lag-phase between clade divergence and diversification' are here affected. Corresponding results should be presented with tractable confidence intervals (i.e. Fig. S3 and Table S1).
7. L. 427-447: This section reads as perspectives regarding chromosomal reshuffling and modular evolution in Brassicaceae. Such speculation is reasonably well-anchored into literature and should be highlighted as such. The presented work however hardly considers 'environmental change' (also see comment 3) or analyses 'pulsating way' (not defined) and perspectives at the very end (L. 442-447) may look disconnected from here-brought insights. Future work should probably consider the role of abiotic and biotic factors on the evolution of morphological and molecular phenotypes, and I agree with the authors that how they integrate with WGD and diversification through time is a fascinating question. I would recommend rewording.
8. In my opinion, Figure 4 is superfluous. Panels (b) and (c) illustrate a verbal model that is not analyzed in details and the core text should be sufficient. Insights for panel (a) would better fit the phylogenetic tree presented in Fig. 3, where both WGD events and rate shifts could easily be mapped. As also mentioned in comment 5, I would recommend to revise Fig. 3 and remove Fig. 4.

Minor comments
--The first paragraph (L. 40-54) anchors this work in macro-evolution and defines 'disparity' based on a few references. Such introduction could be conveyed in half its current length without compromising the understanding.
--Clarifications should be brought in regarding the here-used taxonomic system. L. 98 introduces 51 tribes, whereas L. 173 and L. 184 discuss the analysis of 52 tribes. Some justification of 51 tribes is only brought in L.189 (and more specifically in Supplementary Text 2), which is confusing in a linear reading. An earlier justification would help.
--L.128ff: I personally enjoy introductions ending with a brief statement on main conclusions as to prepare the reader for forthcoming results. Here, L.128-138 represent a lengthy discussion that would better fit that section and that should be streamlined before results have been presented.
--L.154-157: Instead of the expected results, prior studies with similar aims but less comprehensive sampling are discussed This should be introduced before, as to justify objectives of this study.
--L. 218: Those '12 and 24, respectively' are unclear, being described more carefully only at L. 465, and may here be skipped.
--L. 236: The focus of main results is on tribes. Description of methodological validations of proxies at different levels could be mostly brought in the Methods section to here privilege a streamlined presentation. As to further ease the reading of that paragraph, I would suggest to further unify supplementary figures presenting contrasts based on different lineage assignments and therefore support conclusion with a single item.
--L. 247: The kind of 'phylogenetic signal' not detected here should be spelled out here.
--L. 347: 'consequently' is here unclear to me.
--L. 349: I would suggest to skip leaf morphology in high-alpine Brassicaceae. Although possibly interesting and worth further investigation, this sounds out of scope here.
--Figure 2 is nice and may further benefit from integration of panels (a) and (b) using similar ordering of morphological traits. The current presentation using classified morphological traits (i.e. A to F

categories) in panel (a) is seemingly neither natural nor bringing much insights as regards to panel (b). As an exemple, the stem category is depauperate as stem leaves are treated with other, mostly rosette leaves and I have found no clear justification in Supplementary Text 3. Please, clarify in the core text and possibly make it more reader-friendly.
--Legend of Fig. 2 should make it clear that the DAPC is based on tribal disparity.

Reviewer #3 (Remarks to the Author):

This is a very impressive and uniquely detailed reassessment of the phylogeny and classification of Brassicaceae, onto which is superposed an analysis of evolutionary trends in morphological disparity.

Such a powerful analysis, however, suffers from the inadequate definition or characterization of two fundamental concepts:

(i) diversification rate shifts are treated as causal processes with possible effect on morphological disparity. For example, "We show that iterative WGDs in concert with diversification rate shifts positively influenced morphological disparity" (lines 31-33) and even in the title of a section: "Rate shifts and polyploidization as drivers of morphological disparity and diversification" (line 278). But rate shifts are patterns rather than processes, thus cannot be the cause of something, but only correlated with something.
(ii) throughout the article, 'disparity' (admittedly, not an easy concept to define) is used in a less than strictly consistent way. The most critical point (line 332) is highlighted on the annotated ms. returned with these comments.

A number of minor suggestions are also pasted on the annotated ms.

Alessandro Minelli

Reviewer #4 (Remarks to the Author):

see attached file

Reviewer 1

Walden et al. „Nested whole-genome duplications coincide with diversification and high morphological disparity over geological times in the Brassicaceae"

Abstract

I was wondering about „positively" (and „negatively")….if the term is the best to describe increased morphological disparity. I was puzzled by „a modular manner" and „modularized Brassicaceae genomes". I do not know what this is.

Introduction

Does „At-α preceding the divergence of the basal Aethionemeae" mean that all other non-Aethionemeae crucifers diverged from the Aethionemeae. The current wording implies that at-alpha was key only for the Aethionemeae and that all other crucifer clades evolved from this tribe or even did not experience at-alpha.

line 105. „so called mesopolyploidization events" Please add a ref. (31) for the first usage of the term.

l. 108: add the relevant reference here and where appropriate – Mandakova, Lysak (Curr Opin Plant Biol. 2018; 42:55-65).

l. 125: „We then show that mean morphological disparity has increased after WGD, but rather than affecting the entire family, the signal is seen on tribal level only, whereas major evolutionary lineages do not display significantly different disparity values throughout" I was not sure how to understand this sentence: if a mesopolyploidization events are post-dating the Alpha-WGD, these should not affect the entire family (= no wonder) and if there were no meso-WGDs specific for lineages (super-tribes), this should be said in addition to "do not display significantly different disparity values"

l. 136: the sentence starting "Our study thus highlights…" I consider this statement to be a simplification and over-interpretation. It is really hard to believe that not a single study showed the importance of WGD or WGDs for evolutionary success of a (plant) lineage over more than 30 my…

Results

I failed to identify any argumentation why plastome-based phylogeny was used instead of a nuclear multi-gene phylogeny/ies, as for example by Nikolov et al. (New Phytol). Considering presumably prevailing maternal inheritance of plastid DNA in Brassicales, the used phylogeny essentially represents only the maternally inherited relationships. It should be acknowledged or explained why plastome-based phylogenetics was preferred.

l.182. Although I acknwledge the importance of assigning genera to tribes as well as other details given here, I question whether this part should not be moved to the Supplement, as the focus of the paper is different.

l. 208. This paragraph should not be placed in the Results section; please move it to Introduction.

*l. 218, "*a high number of dimensions were needed to explain at least 60% of the variation (12 and 24, respectively),…" Please revise this part to elucidate what is meant by "12 and 24, respectively".

Figure 1 and corresponding analyses/interpretation. While the ref. is given for mesopolyploidization events (legend of Fig. 1), I did not identified a WGD specific for Stevenieae in Mandakova et al. On the contrary, WGDs in Leavenworthia (Caradmineae) and Schizopetaleae were not considered. Authors should explain these inconsistencies.

l. 270: „The three lineages I, II and III were well-separated in the resulting scatterplot (Fig. 2d), however splitting lineage II further27,29 272 resulted in overlapping groups (Supplementary Figure

S9).” Please explain to a reader why lineage II should be further split and why lineage I and III not; this is unclear in the given context.

l. 282-286: Here “association” is used three times. However, it is not apparent what kind of association is meant here, i.e weak/strong or statistically significant?

l. 288: This is true but an attempt was made for the tribe Microlepidieae (Mol Ecol 26, 2017).

Discussion

I was puzzled by the very last sentence as it seems to negate the preceding sentence on nested WGDs and previously stated facts. If WGDs are evolutionary significant and Lineage III does not contain any WGDs, how the named processes are not significantly different among lineages? If there are not apparent differences between the three lineages, then the lack of nested WGDs in Lineage III must be compensated by increased speciation rates and/or decreased extiction rates…

Line 427 onwards.

To say that an ancestral Brassicaceae genome had 8 chromosomes is a simplified statement as the Alpha tetraploid certainly had not 8 chromosome pairs and this number was almost certainly not characteristic for a diploidized ancestral genome of Lineage I/II/III. Moreover, the most recent ancestral genome model introduces 8 chromosomes and only 22 blocks (i.e. the number of blocks is somewaht arbitrary). Genomic blocks are not rearranged only in mesopolyploid genomes, but equally in paleotetraploid genomes, such as ancestors of Arabidopsis thaliana, Camelina, Turritis, etc.

„This may facilitate future studies…“ I was not able to understand what is meant by „This“. The entire paragraph should be either omitted or substantially rephrased to avoid the current usage of engigmatic terms and implying that we were transported several decades back to the past. I assume that the authors simply mean that we can sequence „all“ the Brassicaceae genomes, assemble these and then compare in detail positions of the key genes and their regulatory sequences. If doing so, we should be able to understand what genes are responsible for the (not) observed morphological disparities and how important is the position of these genes within the (epi)genome. If any gene-rich DNA segment breaks or is moved (inversion, traslocation) to another genomic position, the genes will occur in a new environment and their expression can be modulated/altered. This means that we do not need to stay with a rough system of genomic blocks, but we have tools (sequencing and sequence alignments) to get to the roots of these processes.

I was not able to read phylogenetic trees presented as supplementary figures. Please make sure that you provide the highest possible resolution of these figures or, alternatively, a link allowing to evaluate all the essential information.

Reviewer 4

Walden et al. have compiled a beautiful set of data that are indeed important for Brassicaceae and angiosperm research. The morphological data were previously available but they are analyzed in a useful and interesting way here. The plastid genomes (newly added to existing sets) will have wonderful utility for future work on Brassicaceae plastome and genome evolution generally. However, I do have some serious concerns related to the main points that form the focus of the manuscript. The main issues are noted below.

Major concerns:

My primary concern related to this paper is that the two key elements, the confident resolution of phylogenetic relationships and confident dating of nodes, are not currently available for Brassicaceae. Both are absolutely required for this study, but they remain quite underdeveloped for the family. The lack of these two elements largely prevents, in my view, well supported results relating to the roles of WGD and divergence on Brassicaceae. Despite these issues, there are many papers on this topic – in fact this article is second major contribution on the topic from 2019 from the same lead authors using a fair bit of the same data and similar conclusions (see "Temporal patterns of diversification in Brassicaceae demonstrate decoupling of rate shifts and mesopolyploidization events" in Annals of Botany 2019). A well-resolved and well-sampled phylogeny (based on nuclear data) for Brassicaceae will certainly be coming in the next few years, so I believe we are not far from seeing that problem resolved (major steps forward already exist but are ignored in the manuscript). The issues related to precise dating of Brassicaceae phylogeny may never be resolved (because of a lack of fossil evidence), so… it is really up to the editors to decide if this manuscript is significant or not for the journal. Below is a brief elaboration on these major concerns. But I ask myself, why keep writing papers on this until at least one of these two issues has been resolved in a serious way – the conclusions drawn are questionable and the advances minor relative to what has already been published on the Brassicaceae and these topics.

1) **Is the phylogeny presented what is required for the work at hand?** While this is an exceptional plastid gene-tree – something new for the Brassicaceae, there is a serious question about whether a single plastid gene-tree is what is necessary to address the fundamental diversification in the family. Time and time again, the Brassicaceae plastid phylogenies have differed considerably from bipartentally inherited nuclear phylogenies. This issue has most recently been raised in a fairly comprehensive 1800+ exon-based tribal phylogeny of the Brassicaceae (Nikolov et al – feb 2019) – that is cursorily disregard in manuscript under review. The fact that a massive sampling of biparentally inherited nuclear estimates, including a comprehensive analysis of the conflicts noted therein, differ from what is a single locus plastid phylogeny (though there are 60 genes in the plastid, there is no biological reason any should track different phylogenetic history – so the plastid is a single locus), highlight more than a difference between "maternal" and "paternal" histories (as implied in this manuscript). There appears to be a more serious systematic issue in regard to these differences, and modern phylogenetics approaches do not accept the "single locus" estimates as the best solution when conflicts like this arise. In contrast, this paper, in 2019, relies entirely on that single locus plastid phylogeny. They try to make some excuses (lines 152-160) why the available nuclear phylogenies aren't appropriate … but the arguments are weak and even potentially misleading. This issue worries me. Furthermore, after dismissing the biparentally inheritated nuclear data that clearly demonstrate there is conflict that will be difficulty to resolve among some major

R

lineages of Brassiceae (which is not a surprising find), the authors here make the bold "follow on" statement "Our phylogenetic analysis resulted in a fully resolved maternal topology which is summarized…". Selecting the single best tree as an argument for why this result is "better" and suited for this study isn't appropriate in my mind. There is well known conflict and issues with Brassicaceae phylogeny and this manuscript ignores this important element of Brassicaceae evolutionary history. Instead the manuscript suggests they have somehow solved this massive issues. These issues have not been solved.
Other issues with the phylogeny: 1) It seems extremely unlikely that 60 cpDNA genes, including the diversity of protein coding, tRNA, and rRNA genes, all share the very same model of evolution for phylogenetic analysis… as noted in the methods. 2) How were poorly supported nodes treated from the phylogeny? 3) Line 533 – the use of N's to replace missing data in phylogenetic analysis is not technically sound (this is well documented in the literature) - "Ns" are interpreted as a present base (as an A, G, C, T, or polymorphism) – not as missing values That said, there don't seem to be shockingly few N characters in the matrix… there is less than 0.02% Ns in this analysis (based on the date matrix provided). That alone seems quite odd. If missing data were replaced with Ns, then indels were removed with GBLOCKs, there should still be quite a few Ns in matrix. The methods and resulting matrix don't seem to make sense here.

2) **What fossil/calibration information is needed for this work?** It's important to point out that there are no Brassicaceae (or even tribal Brassicales) calibration points used in this study – all calibrations are outgroup based calibrations from quite divergent lineages. The manuscript is quite clear on this. This isn't an oversight of the authors, it reflects the reality for Brassicaceae presently – there are only a few, quite controversial, potential calibrations points known to help date lineages within the order or family. So the authors have avoided these. But to set this in context, the approach taken would be akin to only using fern/fern allies/gymnosperms to try and accurately date divergences in the Angiosperms (see figure below)… in my view this is a serious problem for a study that absolutely requires robust calibration points to deal with variation and rate heterogenetity. I am unaware of such an approach having been applied to any other tribe or family and suspect that those who study the pitfalls of dating may seriously question a result from such distant calibration points.
As an example of the importance… dating approaches try to deal with rate heterogeneity, but the methods require ample well-distributed calibration points to have any hope of working effectively. Having a study with "outgroup only" calibration points is quite dubious to me. The figure below visually illustrates the issue… this is a quick RAxML tree from the dataset provided by the authors. One can easily see the degree of divergence between in the outgroups (and their calibration points) and the ingroup of interest. Even within the ingroup, there is either insufficient sampling or considerable rate heterogeneity. All of these are issues raise serious questions about robust dating procedures. The figure below was developed because Supplementary figure S1 (which is a rough equivalent to this figure) is illegible in the form provided (you can zoom in but not read anything – the same is true for Figure S2 – not remotely readable in the version provided). I suspect the lack of readability had to do with a conversion issue at the journal rather than the authors original version.

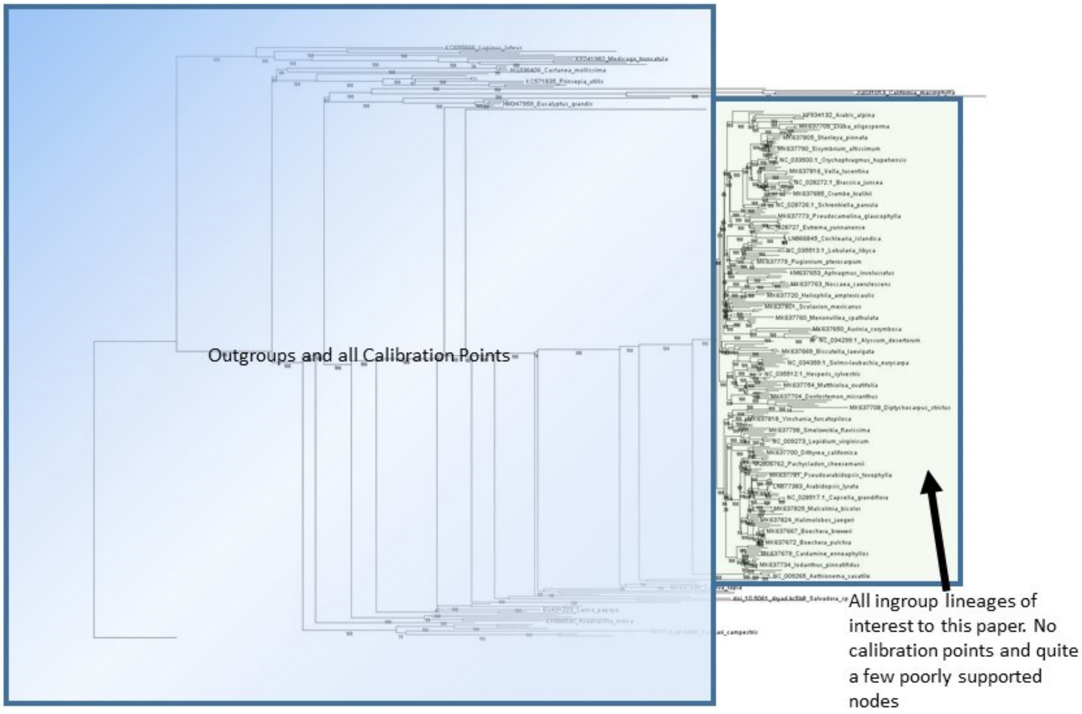

When we consider that the manuscript combines potentially inaccurate phylogenetic resolution, ignores known phylogenetic conflict, and applies questionable calibration points… I have reservations about how these beautiful data (the plastid genomes in particular) are being applied. There are so many wonderful directions to go in for these data… this wasn't one of them in my mind.

General concerns that are more easily resolved:

How have the authors dealt with poorly supported nodes – they report on line 161 '"a fully resolved maternal phylogeny", but the methods applied will always give a fully resolved tree. That doesn't mean there aren't poorly supported nodes therein. Based on the quick tree above, there are poorly supported nodes therein (but I can't read Supp Figs1-2).

Line 169-170. The manuscript notes that their cpDNA findings are congruent with nuclear genome estimates. This isn't accurate and even contradicts what is stated in the introduction of the manuscript. The studies referenced in the manuscript for "congruence" didn't use "nuclear genome" estimates in any modern sense. The two papers that have actually developed a "nuclear

genome" estimate are not cited and significant elements are not congruent. The papers cited by these authors are as follows:

25 – this paper is plastid phylogeny…
32 – this paper is a plastid phylogeny with some a single nuclear locus (ITS)
36 – plastid phylogeny
37 – This paper includes RNA-seq data, but that data was not used for phylogenetics. The tree used in this paper was a single plastid gene (ndhF) plastid with one nuclear locus (phyA)

Lines 194-196. The authors directly note that they have made, what sound like, formal taxonomic changes to 860 species. I don't think they have really done this. I don't see such a vast formal taxonomic in the paper or supplementary materials? Please clarify.

Line 212 – there has been much discussion about the potential phylogenetic utility of Brassicaceae characters. Please provide citation of prior work in some way to support the statements.

Line 235 – what does "realization" of morphological characters really mean? I found this fairly confusing. Can you please define more clearly somewhere?

Line 249 – In numerous places terms are used inconsistently in the manuscript. For example, the morphological matrix has characters for "Duration". But in the main manuscript this is sometimes called "Duration", other times referred to as "life cycle" (line 249) and sometimes called "life form" (line 327). Please be consistent to avoid confusion with this an other terminology.

Line 261-263. This discussion of phylogenetic signal is quite confusing and unclear. If "it" isn't referring to characters, what is "it" referring to? What is "it" later in the sentence.

Line 280 – awkward sentence. Species and genus richness can be represented by numbers, but they aren't numbers as written in the sentence.

Line 313 – please add "Brassicaceae" to the sentence. It sounds like a very general statement, but I think the manuscript is referring to Brassicaceae specifically at the beginning of this paragraph.

Line 327-328 – Sentence doesn't make sense to me

Line 331 – Poorly argued topic. Just because characters have homoplasy doesn't remotely mean they cannot be used phylogeny reconstruction. If that were true, most all molecular data would be useless as well. The issue being discussed is more likely a combination of limited character space (low information content) combined with high homoplasy. Please clarify and make a clear argument.

Line 333 – similar pattern to what? This was unclear.

Start at line 342- I find a fair bit of the Discussion unclear on the main points. For example – what is the point of the second paragraph of the Discussion (lines 342-379)? It's fundamentally

a list of examples without context? It would be much better to indicate what the topic is and how these examples illustrate some critical point.

Line 348. As I recall, this reference (#35) does not support what is stated in this manuscript. I'm fairly sure that paper only demonstrated loss of RCO in A. thaliana, not "repeated loss throughout the family". Please check this citation really supports what is said – my apologies if my memory fails me on this point.

There are a number of places in the manuscript where the authors broadly refer to "homoplasy" as being important. Homoplasy is a general term non-specifically covers important mechanisms (e.g., parallelism and convergence). I think that more development of text related to these underlying mechanisms is warranted (rather than use of a blanket less-specific term "homoplasy"). The mansuscript is ulitimately hitting on important issues of parallelism and convergence… but the text never reaches that level of clarity.

Line 370 – why start this paragraph with a specific statement about character f27? Why not give the reader a clue as to what the paragraph will be focused on (the topic of the paragraph)? This could help make the Discussion easier to follow.

Line 391-393. Perhaps the sentence is correctly written, but I found it quite confusing. If I were to rewrite it, I believe it literally says "speciation rates are lowest after WGDs because of considerably lower extinction rates after WGDs"? Is this accurate? Seems odd to conclude that "low extinction leads to low speciation"?

Line 427 – what is "this" – please be more specific to avoid the reader having to interpret exactly what is meant.

Line 438 – I like this paragraph in general, but here the use of "homoplasy" again isn't useful. Homoplasy is a reflection of other more specific topics and it would be far better to talk about those than blanket term "homoplasy" (e.g., parallelism and convergence).

Lines 442-447. I didn't find this concluding paragraph to be very convincing. It's a big broad statement and conclusion. I would suggest either remove this or develop the topic into a meaningful paragraph or more worthy of the magnitude implied. Since this seems to be the major "conclusion" – I think much more could be done to tie the ideas into the context of what is already known on the topic. Note that there isn't any discussion on the existing literature that relates to the development of the model presented by the authors.

Line 459 – why is geography relevant to this paper? I don't see it in any data matrix?

Section beginning line 482. How much of this data was used in the early 2019 papers (Huang et al 2019 and Nikolov et al 2019)? I don't think this is all new data. Please be precise.

Why didn't the group develop a ribosomal phylogeny from all this beautiful skimming data? I suspect it would show considerable conflict, that is important but ignored. Developing the nrDNA phylogeny is pretty standardard with genome skimming data like this.

Line 533. Another example of potentially confusing choice of terms. The authors refer to protein coding genes, tRNA, and rRNA as "coding genes". It is generally accepted that "coding genes", as a term, is an abbreviation for "protein coding genes". tRNA and rRNA are not "coding genes" to most readers. Probably best to just say they used "60 genes, including 43 protein coding, 14 tRNA, etc."

Line 643 – several lines earlier the authors state that the Bivonaeeae wasn't sampled… now it says it was sampled? Please clarify and correct.

Line 646 – "respective numbers" of what? What is this?

Line 663 – lack of resolution from one locus isn't acceptable as evidence for a "hard polytomy"… This is a place to talk about what the recent nuclear genomic phylogenies have found here. Ignoring other data and calling this a "hard polytomy", based on plastid tree, doesn't make sense.

Line 675 – insert "morphological" before "dispartity"

Supplementary Figs S1 and S2 are completely unreadable – even when zooming.

Reviewers' comments:

Reviewer #1 (Remarks to the Author):

This an interesting and unique attempt to evaluate morphological disparity across the mustard family (Brassicaceae) using a family-wide plastome phylogeny. The phylogeny is the most comprehensive plastome phylogeny published for the crucifers. I also highly evaluate the attempt to evaluate the evolutionary significance of whole-genome duplications for speciation rate shifts and net diversification rates across the Brassicaceae. I have only a few minor comments summarized in my review attached:

Abstract

I was wondering about „positively" (and „negatively")….if the term is the best to describe increased morphological disparity. I was puzzled by „a modular manner" and „modularized Brassicaceae genomes". I do not know what this is.

We rephrased the sentence with 'positively' to emphasize the correlative rather than causal nature of this association.

We removed the genome structure parts from the abstract (following also reviewer 2's comment). The reviewers are right to comment on this, since we did not analyze genome structure.

Introduction

Does „At-α preceding the divergence of the basal Aethionemeae" mean that all other non-Aethionemeae crucifers diverged from the Aethionemeae. The current wording implies that at-alpha was key only for the Aethionemeae and that all other crucifer clades evolved from this tribe or even did not experience at-alpha.

Yes, the wording was misleading. We rephrased it to make it clearer.

line 105. „so called mesopolyploidization events" Please add a ref. (31) for the first usage of the term.

Yes, we added this reference here.

l. 108: add the relevant reference here and where appropriate – Mandakova, Lysak (Curr Opin Plant Biol. 2018; 42:55-65).

Yes, we now cite this paper.

l. 125: „We then show that mean morphological disparity has increased after WGD, but rather than affecting the entire family, the signal is seen on tribal level only, whereas major evolutionary lineages do not display significantly different disparity values throughout" I was not sure how to understand this sentence: if a mesopolyploidization events are post-dating the Alpha-WGD, these should not affect the entire family (= no wonder) and if there were no meso-WGDs specific for lineages (supertribes), this should be said in addition to "do not display significantly different disparity values"

This paragraph has been rephrased.

l. 136: the sentence starting "Our study thus highlights…" I consider this statement to be a simplification and over-interpretation. It is really hard to believe that not a single study showed the importance of WGD or WGDs for evolutionary success of a (plant) lineage over more than 30 my…

Yes, this was not expressed clearly. We rephrased to 'Our study thus highlights *the evolution of morphological diversity as* a so far underexplored aspect of the general importance of WGDs for evolutionary success of a lineage over more than 30 million years.'

Results

I failed to identify any argumentation why plastome-based phylogeny was used instead of a nuclear multi-gene phylogeny/ies, as for example by Nikolov et al. (New Phytol). Considering presumably prevailing maternal inheritance of plastid DNA in Brassicales, the used phylogeny essentially represents only the maternally inherited relationships. It should be acknowledged or explained why plastome-based phylogenetics was preferred.

Our main reasons for preferring plastome-based phylogenetics were divergence time estimates (lacking in Nikolov et al.), availability of samples from difficult-to-access tribes (genome skimming from herbarium material), in particular for complete stem/crown group age estimates. While our approach indeed only considers the maternal phylogeny, our analyses are mostly conducted on tribal/supertribal level (thus a conflicting backbone phylogeny is of minor impact). In addition, the backbone is also not completely resolved in Nikolov et al. (support for many deeper nodes is low).

However, we agree with the reviewer that justification should be given, and therefore added a short paragraph explaining our reasoning at the beginning of the results section.

l.182. Although I acknowledge the importance of assigning genera to tribes as well as other details given here, I question whether this part should not be moved to the Supplement, as the focus of the paper is different.

We shortened this paragraph in the main manuscript considerably.

l. 208. This paragraph should not be placed in the Results section; please move it to Introduction.

OK, we moved it to the introduction.

l. 218, "a high number of dimensions were needed to explain at least 60% of the variation (12 and 24, respectively),…" Please revise this part to elucidate what is meant by "12 and 24, respectively".

Following the comment by reviewer 2 regarding this paragraph, we removed this part from the results section entirely (while keeping it in the methods section) to allow for better readability.

Figure 1 and corresponding analyses/interpretation. While the ref. is given for mesopolyploidization events (legend of Fig. 1), I did not identified a WGD specific for Stevenieae in Mandakova et al. On the contrary, WGDs in Leavenworthia (Caradmineae) and Schizopetaleae were not considered. Authors should explain these inconsistencies.

Indeed, the WGD in Stevenieae was not identified in Mandáková et al., (2017, Plant J) but in Kiefer et al. (2019, Nat Plants); we added the reference whenever the analyzed mesopolyploidization events are mentioned in the text. *Leavenworthia* (Cardamineae) was not considered, because this WGD is most likely restricted to the genus *Leavenworthia* – it is not shared with *Cardamine*. The WGD in *Schizopetalon* was originally not considered because we had restricted our analysis on tribes where genomic evidence for the WGD was available. However, we now include Schizopetaleae in the tribes with WGDs; this had no impact on significance in phylogenetic ANOVAs.

l. 270: „The three lineages I, II and III were well-separated in the resulting scatterplot (Fig. 2d), however splitting lineage II further27,29 272 resulted in overlapping groups (Supplementary Figure S9)." Please explain to a reader why lineage II should be further split and why lineage I and III not; this is unclear in the given context.

We analyzed the data following three different lineage assignments to acknowledge that our phylogeny and the splitting of Brassicaceae into basal Aethionemeae and three major lineages is not the only possible way to group Brassicaceae tribes. The differences between authors

exclusively concern lineage II, which is split into two (Franzke et al.) or three (Nikolov et al.) lineages, while lineage I and III are generally not split further.

We rephrased the sentence to make this more obvious to the reader: '…however splitting lineage II further following lineage assignment proposed by Franzke et al. and Nikolov et al. resulted in overlapping groups…'.

l. 282-286: Here "association" is used three times. However, it is not apparent what kind of association is meant here, i.e weak/strong or statistically significant?

Statistically significant ($p < 0.05$); we changed 'association' to 'correlation' whenever appropriate, but kept 'association' when one of the variables was binary (e.g. WGD vs. no WGD).

l. 288: This is true but an attempt was made for the tribe Microlepidieae (Mol Ecol 26, 2017).

Yes. We now specifically mention tribe Microlepidieae and cite this paper.

Discussion

I was puzzled by the very last sentence as it seems to negate the preceding sentence on nested WGDs and previously stated facts. If WGDs are evolutionary significant and Lineage III does not contain any WGDs, how the named processes are not significantly different among lineages? If there are not apparent differences between the three lineages, then the lack of nested WGDs in Lineage III must be compensated by increased speciation rates and/or decreased extinction rates…

This is correct and the half-sentence "significantly different among lineages …" was misleading. We deleted this sentence.

Line 427 onwards. To say that an ancestral Brassicaceae genome had 8 chromosomes is a simplified statement as the Alpha tetraploid certainly had not 8 chromosome pairs and this number was almost certainly not characteristic for a diploidized ancestral genome of Lineage I/II/III. Moreover, the most recent ancestral genome model introduces 8 chromosomes and only 22 blocks (i.e. the number of blocks is somewhat arbitrary). Genomic blocks are not rearranged only in mesopolyploid genomes, but equally in paleotetraploid genomes, such as ancestors of Arabidopsis thaliana, Camelina, Turritis, etc.

Yes, it's 22 blocks according to Lysak et al. (2016). We updated this. Following the meaningful comment of the reviewer we deleted the speculation about ancestral chromosome number. It does not contribute to the chapter.

„This may facilitate future studies…" I was not able to understand what is meant by „This". The entire paragraph should be either omitted or substantially rephrased to avoid the current usage of enigmatic terms and implying that we were transported several decades back to the past. I assume that the authors simply mean that we can sequence „all" the Brassicaceae genomes, assemble these and then compare in detail positions of the key genes and their regulatory sequences. If doing so, we should be able to understand what genes are responsible for the (not) observed morphological disparities and how important is the position of these genes within the (epi)genome. If any gene-rich DNA segment breaks or is moved (inversion, translocation) to another genomic position, the genes will occur in a new environment and their expression can be modulated/altered. This means that we do not need to stay with a rough system of genomic blocks, but we have tools (sequencing and sequence alignments) to get to the roots of these processes.

Yes, we shortened this sentence accordingly.

I was not able to read phylogenetic trees presented as supplementary figures. Please make sure that you provide the highest possible resolution of these figures or, alternatively, a link allowing to evaluate all the essential information.

We have included the pdfs on the google drive for supplementary files. We also now provide a higher quality pdf of the supplements.

Reviewer #2 (Remarks to the Author):

Review of "Nested whole-genome duplication coincides with diversification and high morphological disparity over geological times in the Brassicaceae"

This work by Walden et al presents a detailed phylogenetic analysis of the genus and tribes across Brassicaceae, addressing the morphological space realized by diversifying taxa with a focus on the role of whole genome duplication (WGD). This is a comprehensive and timely contribution to the field of plant evolution, bringing fresh insights on current debates regarding interactions between WGDs and diversification. The amount of data here integrated within a fine phylogenetic network is impressive and paves the way towards future work on forms and function in this model family for evolutionary studies.

The manuscript is well written, with detailed descriptions yielding confidence in sound data and approaches. Although some paragraphs may be streamlined to improve the impact and avoid unnecessary redundancy (e.g. between results and methods), the presentation is convincing and likely to raise interest among plant scientists and evolutionary biologists.

Here below, I will expend on a few issues that would benefit from further clarifications and that may strengthen the links between data and their interpretations.

Detailed comments

1. The end of the abstract is frustrating as it summarizes insights such as gain and loss of characters and genome modularity that are mostly discussed as perspectives rather than part of core results presented here. Such perspectives would be better discussed as such in the main text (also see comment 7), while not being mentioned in the abstract.

We removed the mentioning of genome modularity from the abstract.

2. The introduction starts by introducing the process of WGD and then discusses 'polyploidy'. As the later concept also encompasses reticulate evolution (i.e. allopolyploidy) and given that this work is focused on maternal relationships based on plastid loci (i.e. cannot identify reticulations), some effort should be brought here (L.64) and throughout the manuscript to unify the terminology and make sure that readers are not being confused.

We added a sentence here to introduce that both, auto- and allopolyploidy contributes to WGD – but most often it is not explicitly stated for paleopolyploids if they are auto- or allopolyploids.

It is correct that we cannot elaborate on this issue and we added another sentence accordingly.

But just one thought here:

If past WGDs were largely driven by allopolyploidization, then there is no "true" cladogenetic tree and our approach to use the plastome as maternal perspective should be an adequate way to analyze the data. Alternatively, if past WGDs were largely the result of autopolyploidization then we must not consider complex reticulate scenarios and may rely also on the presented phylogeny (considering, of course, comments provided e.g. by reviewer 4).

3. Although I acknowledge that the authors here loyally summarize (L. 79ff) the 'WGD radiation lag-time model' as originally presented, the ground for such a chain of event remains unclear. In particular, following e.g. Wagner (2017, The White-Knight hypothesis, or does the environment limit innovations?, Tr EcolEvol 32:131–140), why should 'key traits or innovations' be expected to evolve early and not

later (e.g. at dispersal/radiation stages)? While the model here under scrutiny subordinates evolutionary radiation to shifts in distribution ranges and (possibly) environmental changes, this study does not address such factors and it remains unclear to what extent present results support or reject this model. I would recommend to clarify the background, and maybe use this 'lag-time model' only as a metaphor to enlighten results from L. 410 on.

The reviewer is right and we shortened the summary of the WGD radiation lag-time model in the introduction following this suggestion.

4. Related to the previous comment, factors underlying 'disparity' should ideally be teased apart. WGD supporting reproductive isolation, it may promote the accumulation of morphological differences (e.g. L. 55) and thus support diversification as here evaluated (e.g. L. 283 and L. 293). The combination of factors driving disparity should be further quantified and discussed, and possibly integrated with views of a 'lag-time model' following WGD; at least verbally (e.g. L. 410).

This is a meaningful comment, however, we do not see a reliable way to quantify and subsequently test significance of combining factors (such as "environmental major change") in a more numerical/statistical way (e.g. because case-number is not sufficient, and most often we still do not have a precise idea about the spatial-geographic context on tribal level) aside what we have done and presented herein. Therefore, we followed the suggestion and comment on these aspects with views of a "lag-time model" as suggested by the reviewer with few words and mentioned the restrictions of our work accordingly.

5. Available insights regrading mesopolyploidy events should be further introduced and discussed. In particular, it remains unclear to what extent such events discovered in 11 tribes (L. 105) are representative of mesopolyploidy across Brassicaceae. Were the 40 remaining tribes demonstrated as lacking WGD events? Corresponding clarifications should be brought in as to support unbiased interpretations L. 170 (incl. Fig. 1) as well as specific contrasts based on 'WGD vs no WGD' (L. 292ff). On top of justifications, Fig. 3 should present sample sizes and map WGD events on the phylogenetic tree. When it comes to corresponding issues in the discussion (L. 397ff), it would indeed be great to know more about those 129 genera 'affected by additional mesopolyploidization' as well as the ca. 222 remaining. Similar justification may help to support the particularly high WGD rate exhibited by Brassicaceae.

We reworked Fig. 3 to include a) sample sizes for comparisons of disparity in tribes with/without WGDs and rate shifts displayed as boxplots; b) number of genera (sampled in our subset tree and total) per tribe, as well as mesopolyploidization events and rate shifts (species number based) mapped on the phylogenetic tree. Note that while these 11 mesopolyploidization events are presumed to be old and occurring at or near the base of the tribes, the exact age and position is often unknown – we therefore prefer to show mesopolyploidization next to rather than on the tree. The rate shifts detected in 9 tribes (following Huang et al., 2019) are also displayed next to the tree, as our plastome tree contains only a subset of Brassicaceae species, and the branches where rate shifts occurred are not always present in our tree.

We furthermore added a paragraph describing how representative the detected mesopolyploidizations most likely are (in the methods section): '*Mesopolyploidization events were found at or near the origin of eleven tribes (excluding the WGD detected in Leavenworthia, Cardamineae, as it is restricted to this genus and not shared with the rest of the tribe) and shown to be absent in at least twenty tribes, most notably in all analyzed tribes of lineage III. For a number of other tribes, low base chromosome numbers and genome sizes are consistent with a lack of mesopolyploidization, while for few, neither data nor material for cytogenetic or sequence-based analyses is available*'. The summarized information and evidence are also given in a Suppl. Table in the appendix. A short notice is also provided with the Introduction.

Additionally, we rephrased the sentence in the discussion 'In total *130* genera are *potentially* affected by *one of the* additional *eleven* mesopolyploidizations'.

6. L. 175ff: Correlation between crown group ages here inferred and available from prior studies based on nuclear loci is based on only 24 data points. As such dating is associated with uncertainty, it remains unclear how estimates of 'lag-phase between clade divergence and diversification' are here affected. Corresponding results should be presented with tractable confidence intervals (i.e. Fig. S3 and Table S1).

We agree with the reviewer and added upper and lower 95% HPD intervals to Fig. S3 and Table S1.

7. L. 427-447: This section reads as perspectives regarding chromosomal reshuffling and modular evolution in Brassicaceae. Such speculation is reasonably well-anchored into literature and should be highlighted as such. The presented work however hardly considers 'environmental change' (also see comment 3) or analyses 'pulsating way' (not defined) and perspectives at the very end (L. 442-447) may look disconnected from here-brought insights. Future work should probably consider the role of abiotic and biotic factors on the evolution of morphological and molecular phenotypes, and I agree with the authors that how they integrate with WGD and diversification through time is a fascinating question. I would recommend rewording.

The reviewer is right, and we re-phrased this speculative paragraph, which is not substantiated by our data in a strict sense.

8. In my opinion, Figure 4 is superfluous. Panels (b) and (c) illustrate a verbal model that is not analyzed in details and the core text should be sufficient. Insights for panel (a) would better fit the phylogenetic tree presented in Fig. 3, where both WGD events and rate shifts could easily be mapped. As also mentioned in comment 5, I would recommend to revise Fig. 3 and remove Fig. 4.

We restructured Figure 3 following these suggestions, and now show WGDs and rate shifts next to the phylogenetic tree. Figure 4 was removed since this, indeed, reflects a "verbal model", which we cannot test explicitly.

Minor comments

--The first paragraph (L. 40-54) anchors this work in macro-evolution and defines 'disparity' based on a few references. Such introduction could be conveyed in half its current length without compromising the understanding.

We do think that it is plausible to introduce the text as it stands. It is also reviewer 3 asking to define/explain disparity and, therefore, setting the context. We also feel that it is important to make a link to the animal field, since in higher plants there is still very limited information on this topic. We hope that the reviewer agrees. Otherwise, of course, we can shorten the paragraph.

--Clarifications should be brought in regarding the here-used taxonomic system. L. 98 introduces 51 tribes, whereas L. 173 and L. 184 discuss the analysis of 52 tribes. Some justification of 51 tribes is only brought in L.189 (and more specifically in Supplementary Text 2), which is confusing in a linear reading. An earlier justification would help.

This is a good point. As it is not crucial to our conclusions, we now moved all mention of tribe Bivonaeeae and its inclusion into Brassiceae to the Supplemental texts and consistently use a taxonomic system of 51 tribes in the main text.

--L.128ff: I personally enjoy introductions ending with a brief statement on main conclusions as to prepare the reader for forthcoming results. Here, L.128-138 represent a lengthy discussion that would better fit that section and that should be streamlined before results have been presented.

We shortened this paragraph accordingly, and we re-phrased it in a way keeping the main conclusion as the reviewer suggested. Indeed, as it was originally presented it should be integrated with the discussion (e.g. summary statement).

--L.154-157: Instead of the expected results, prior studies with similar aims but less comprehensive sampling are discussed This should be introduced before, as to justify objectives of this study.

Yes, we moved this into the introduction section.

--L. 218: Those '12 and 24, respectively' are unclear, being described more carefully only at L. 465, and may here be skipped.

Yes, we omit the details here now.

--L. 236: The focus of main results is on tribes. Description of methodological validations of proxies at different levels could be mostly brought in the Methods section to here privilege a streamlined presentation. As to further ease the reading of that paragraph, I would suggest to further unify supplementary figures presenting contrasts based on different lineage assignments and therefore support conclusion with a single item.

Yes. We omit the details here, as they are described in the methods section, and combined the Supplementary Figures following the reviewer's suggestion.

--L. 247: The kind of 'phylogenetic signal' not detected here should be spelled out here.

We specified the phylogenetic signal analyzed here (Moran's I).

--L. 347: 'consequently' is here unclear to me.

We rephrased this sentence to 'Consistent with the high levels of homoplasy in leaf shape (character D10), we did not detect phylogenetic signal or lineage differentiation in this character.'

--L. 349: I would suggest to skip leaf morphology in high-alpine Brassicaceae. Although possibly interesting and worth further investigation, this sounds out of scope here.

We agree that the context was not clear. We want to demonstrate that for some of the characters there is independent evidence for its relevance in plant fitness. We shortened and re-phrased.

--Figure 2 is nice and may further benefit from integration of panels (a) and (b) using similar ordering of morphological traits. The current presentation using classified morphological traits (i.e. A to F categories) in panel (a) is seemingly neither natural nor bringing much insights as regards to panel (b). As an example, the stem category is depauperate as stem leaves are treated with other, mostly rosette leaves and I have found no clear justification in Supplementary Text 3. Please, clarify in the core text and possibly make it more reader-friendly.

We reordered Fig. 2a to match Fig. 2b. This also helps illustrate the pattern of high pairwise difference in characters with medium disparity described in the first paragraph of the discussion. We kept the admittedly somewhat arbitrary grouping into categories A to F, which has been stated with the Suppl. Text accordingly. The reviewer is right, but we think that it is easier for the reader to follow a character and its states. We added a short notice with the main text as it is found with the Supplement.

--Legend of Fig. 2 should make it clear that the DAPC is based on tribal disparity.

Yes. Clarified now.

Reviewer #3 (Remarks to the Author):

This is a very impressive and uniquely detailed reassessment of the phylogeny and classification of Brassicaceae, onto which is superposed an analysis of evolutionary trends in morphological disparity.

Such a powerful analysis, however, suffers from the inadequate definition or characterization of two fundamental concepts:

(i) diversification rate shifts are treated as causal processes with possible effect on morphological disparity. For example, “We show that iterative WGDs in concert with diversification rate shifts positively influenced morphological disparity” (lines 31-33) and even in the title of a section: “Rate shifts and polyploidization as drivers of morphological disparity and diversification” (line 278). But rate shifts are patterns rather than processes, thus cannot be the cause of something, but only correlated with something.

Yes, the reviewer is correct. We revised all occurrences of ‘diversification rate shifts’ to make the correlative nature of this association more obvious.

(ii) throughout the article, ‘disparity’ (admittedly, not an easy concept to define) is used in a less than strictly consistent way. The most critical point (line 332) is highlighted on the annotated ms. returned with these comments.

Disparity is indeed difficult to define, in part because there are many ways to calculate disparity. In the introduction section, we introduce the term with a simple, very general definition. In the later parts of the manuscript, we always mean the specific measure of disparity we calculate (number of observed states in the taxon in question/number of total states in Brassicaceae). We added a note regarding the use of the term at the first mention of disparity in the results section.

A number of minor suggestions are also pasted on the annotated ms.

L. 41: living organism -> land plant

Yes.

L. 42: factor in -> product of

Yes.

L. 46. The causal link is not clear

We added a half-sentence to clarify this link.

L. 50. See also Minelli A. (2016) Species diversity vs. morphological disparity in the light of evolutionary developmental biology. Annals of Botany 117:795-809.

Indeed. We now also cite this paper.

L. 50: animals -> animal clades

Yes.

L. 61. The causal link is not clear

Yes.

L. 66: value -> potential

Yes.

L. 99. In the tree used in figure 2, Aethionemeae do not branch basally as sister to all remaining Brassicaceae. It may be sensible to use here too figure 1 tree or at least to provide comment/explanation.

Aethionemeae do indeed branch basally to all remaining Brassicaceae. However, there is an additional row of 'unplaced genera' below all other Brassicaceae, and we had not added space between Aethionemeae and lineage III, which made it difficult to see. We now added this space to make the groups more easily distinguishable.

L. 126: WGD -> WGDs

Yes.

L. 249: highest -> most widely distributed

Here, 'disparity' is used as the measure for morphological diversity we calculate. In this sense, highest refers to the calculated value and is more accurate than most widely distributed, which would probably be more appropriate if disparity was calculated from continuous data. See also our answer to comment (ii) above.

L. 332-333. Strictly speaking, this is not low disparity, if two or more well distinct character states occur in a clade, e.g. a tribe, but high predictability of character state, in the sense of information theory.

Indeed. But see comment just above.

L. 368: additional -> alternative

Yes.

L. 372: Which features? Please specify.

We rephrased to '*diverse* pericarp features*, such as corkiness, hooks, barbs and wings*'.

L. 375: Differentiation -> difference

Yes.

Reviewer #4 (Remarks to the Author):

Walden et al. have compiled a beautiful set of data that are indeed important for Brassicaceae and angiosperm research. The morphological data were previously available but they are analyzed in a useful and interesting way here. The plastid genomes (newly added to existing sets) will have wonderful utility for future work on Brassicaceae plastome and genome evolution generally. However, I do have some serious concerns related to the main points that form the focus of the manuscript. The main issues are noted below.

The reviewer states that "*morphological data were previously available*". We explained the workflow in detail with the appendix. And the morphological data were not available before. Of course, there are descriptions of genera and species in floras, keys, etc., but we had not only to collect all this information, we also had to carefully inspect and correct that. Furthermore, we had to re-define nearly any set of character states. This work needed nearly eight years. It is true that the basis of the idea was an earlier and preliminary "interactive electronical key", and of course we also used all data provided with this key designed by Ihsan Al-Shehbaz, who is one of our long-term collaborators (btw: he was celebrating his 80th birthday last year, and, if possible and adequate and being successful to provide an acceptable revision, we aim to dedicate this contribution to him). But a simple "key to genera" is not a morphological character and character state matrix. The matrix was and is not available to the public.

Furthermore, we also worked on this "interactive key" and improved it to be used with BrassiBase and to contribute to enabling researchers to determine genera more accurately.

The wording is also somehow misleading if saying "*newly added (plastomes) to existing sets*". The large majority of Brassicaceae plastomes are not only new, but also fully assembled and annotated at high quality. It is true for all outgroups that we used already published data including also some Brassicaceae plastomes. However, we had to rely on material with accessible and correctly identified herbarium vouchers (e.g. Hohmann et al. 2015), which is often limited using GenBank data. The percentage of already published Brassicaceae plastomes in our study is 28% (71 out of 250). And we continued to sample taxa, which are often very difficult to find in collections (e.g. for taxonomic reasons, differently named, etc.).

It should be also mentioned, that with the Nikolov et al. (2019) paper a smaller plastid tree was shown (congruent to our data), but this was not based on entire and fully assembled plastomes (nine plastomes assembled as in "full circle" therein?).

Major concerns:

My primary concern related to this paper is that the two key elements, the confident resolution of phylogenetic relationships and confident dating of nodes, are not currently available for Brassicaceae. Both are absolutely required for this study, but they remain quite underdeveloped for the family. The lack of these two elements largely prevents, in my view, well supported results relating to the roles of WGD and divergence on Brassicaceae. Despite these issues, there are many papers on this topic – in fact this article is second major contribution on the topic from 2019 from the same lead authors using a fair bit of the same data and similar conclusions (see "Temporal patterns of diversification in Brassicaceae demonstrate decoupling of rate shifts and mesopolyploidization events" in Annals of Botany 2019). A well-resolved and well-sampled phylogeny (based on nuclear data) for Brassicaceae will certainly be coming in the next few years, so I believe we are not far from seeing that problem resolved (major steps forward already exist but are ignored in the manuscript). The issues related to precise dating of Brassicaceae phylogeny may never be resolved (because of a lack of fossil evidence), so… it is really up to the editors to decide if this manuscript is significant or not for the journal. Below is a brief elaboration on these major concerns. But I ask myself, why keep writing papers on this until at least one of these two issues has been resolved in a serious way – the conclusions drawn are questionable and the advances minor relative to what has already been published on the Brassicaceae and these topics.

Our paper from 2019 in Annals of Botany clearly focused on diversification patterns *within* tribes and we compared them accordingly. Datasets fully relied on tribal-wide ITS data and neither taxonomy nor morphology was in focus. The herein presented manuscript clearly has morphological disparity in focus. Therefore, the comment "*advances are minor*" is not true. Nobody has evaluated the entire morphospace of the family before, and nobody provided the taxonomic framework (up-to-date species check-list and critical circumscription of genera) as a

prerequisite for adequate morphological descriptions as in the given context. Furthermore, we expect that all given information is indeed of "major impact" for the scientific community – at least for those working with Brassicaceae.

1) Is the phylogeny presented what is required for the work at hand? While this is an exceptional plastid gene-tree – something new for the Brassicaceae, there is a serious question about whether a single plastid gene-tree is what is necessary to address the fundamental diversification in the family. Time and time again, the Brassicaceae plastid phylogenies have differed considerably from biparentally inherited nuclear phylogenies. This issue has most recently been raised in a fairly comprehensive 1800+ exon-based tribal phylogeny of the Brassicaceae (Nikolov et al – feb 2019) – that is cursorily disregard in manuscript under review.

We explicitly mentioned and discussed the Nikolov et al. (2019) paper, and it appears 8 times in citations with the main manuscript. The same is true for the appendix. Furthermore, we used the evolutionary lineages as defined in Nikolov et al. (2019) to compare them with alternative/other (sub)groupings (Appendix Figures S6 and S8, Tables S3 and S6). However, usage of this phylogeny for our study is limited, because it is neither complete nor time-calibrated. Mentioning in this context that there will be a comprehensive phylogeny available in few years is also highly misleading. The problem is not to include many species. The problem is to get access to reliable material from any deeper node and crucial sister taxa. The presented phylogeny is a consequent result of such an laborious attempt, since we aimed to recover any deeper node in any given tribe. We totally agree with the reviewer, that actually no large-scale phylogeny (neither from the nuclear genome nor from the plastome) is the ultimate answer, and, therefore, we restricted our conclusions and analyses on taxonomy and data aggregation not biasing our conclusions accordingly.

We agree with the reviewer that we should mention this more clearly within the main manuscript – we updated this with the "Results" section, where it fits at best.

The fact that a massive sampling of biparentally inherited nuclear estimates, including a comprehensive analysis of the conflicts noted therein, differ from what is a single locus plastid phylogeny (though there are 60 genes in the plastid, there is no biological reason any should track different phylogenetic history – so the plastid is a single locus), highlight more than a difference between "maternal" and "paternal" histories (as implied in this manuscript). There appears to be a more serious systematic issue in regard to these differences, and modern phylogenetics approaches do not accept the "single locus" estimates as the best solution when conflicts like this arise. In contrast, this paper, in 2019, relies entirely on that single locus plastid phylogeny. They try to make some excuses (lines 152-160) why the available nuclear phylogenies aren't appropriate … but the arguments are weak and even potentially misleading. This issue worries me. Furthermore, after dismissing the biparentally inherited nuclear data that clearly demonstrate there is conflict that will be difficulty to resolve among some major lineages of Brassiceae (which is not a surprising find), the authors here make the bold "follow on" statement "Our phylogenetic analysis resulted in a fully resolved maternal topology which is summarized…". Selecting the single best tree as an argument for why this result is "better" and suited for this study isn't appropriate in my mind. There is well known conflict and issues with Brassicaceae phylogeny and this manuscript ignores this important element of Brassicaceae evolutionary history. Instead the manuscript suggests they have somehow solved these massive issues. These issues have not been solved.

(1) With this manuscript we present a first and comprehensive species checklist, and we had to decide at the very beginning, on the best phylogenetic tool to solve the many open taxonomic issues and problems (covering as many genera as possible and providing highly resolved phylogenies, while being able to work accurately on tribal level). We fully achieved this goal, while inspecting all earlier published systematic-taxonomic work and evaluating case by case.

(2) The taxonomic framework (species and genera) has been the prerequisite to score and prepare a final consistent morphomatrix.

(3) Finally, we analyzed data most often on tribal level and using comparative statistics; and our given plastome phylogeny was basically used to test for influence of phylogenetic signal in the datasets. However, for those tests we cannot use any other phylogeny, because those represent a subset of our data only, and they are not time-calibrated. So it is not "to make some excuses" as reviewer 4 phrased it. However, the reviewer is correct if questioning the use of alternative phylogenetic scenarios. This has been extensively performed, including not only the Nikolov et al. clades, but we also include now a totally new dataset comprising the entire nrDNA cistron (see below). Our results are stable and consistent.

Curious finding after inspecting relevant papers again: In the Nikolov et al. (2019) paper it is stated with Table S9 in the Appendix: "Accession information of marker genes used for infratribal placement" … *ndh*F and *rbc*L: these are genes from the plastome, and it seems that even here authors had to be somehow pragmatic in this respect.

In our revision, we made it more explicit, that the plastome phylogeny is, of course, not the ultimate family phylogeny – but it is an ultimate plastome backbone phylogeny. However, it was never ever our intention to present a final phylogenetic hypothesis on family level. Second, we were able to extract from our genome skimming data the entire nrDNA operon (nuclear ribosomal RNA) of about 8000 bp and to run some analyses. Predictably, the resulting tree is not resolved among tribes [since in the past a fraction of the region (ITS1 and ITS2) has often been used on tribal level systematics, but failed on family level (e.g. Bailey et al. 2006)]. But this analysis also highlights the same problem as with large-scale genomic data and further highlights individual cases (tribes) for which detailed analyses should be done in future.

Other issues with the phylogeny:

1) It seems extremely unlikely that 60 cpDNA genes, including the diversity of protein coding, tRNA, and rRNA genes, all share the very same model of evolution for phylogenetic analysis… as noted in the methods.

It was in fact surprising that a single partition (and thus a single best substitution model) turned out to be the best scheme for the given dataset. Yet, as described in the respective methods section (line 541 – 546) we used PartitionFinder for the selection of the best partitioning strategy, as also commonly applied in other large-scale phylogenetic analyses such as in e.g. Guo et al. (2017), Nikolov et al. (2019), Soltis et al. (2019). The analysis was performed twice in order to confirm the resulting partitioning. Both analyses revealed the same best-fitting partitioning scheme which was therefore used in the following analysis.

Furthermore, since our plastome tree topology for Brassicaceae-relationships is not different from any other presented (smaller) plastome tree published so far, we did not change the manuscript.

2) How were poorly supported nodes treated from the phylogeny?

Support values are shown in Fig. S1. Tribes are generally highly supported (most crown nodes have 100% bootstrap support), and support values for the three major lineages are similarly high (99-100%). These are the nodes that are relevant for most analyses conducted in our study. Lower support values are found within lineages at splits between tribes, in particular in lineage II – this is presumably a result of the rapid diversification of lineages around 20 mya. For our analyses, we furthermore extracted only one stem group age for all tribes of the CES clade, as resolution is low, presumably due to a rapid radiation at the origin of these tribes.

3) Line 533 – the use of N's to replace missing data in phylogenetic analysis is not technically sound (this is well documented in the literature) - "Ns" are interpreted as a present base (as an A, G, C, T, or polymorphism) – not as missing values That said, there don't seem to be shockingly few N characters in the matrix… there is less than 0.02% Ns in this analysis (based on the date matrix provided). That alone seems quite odd. If missing data were replaced with Ns, then indels were removed with GBLOCKs, there should still be quite a few Ns in matrix. The methods and resulting matrix don't seem to make sense here.

As stated in Line 529, "In general, only genes present in all taxa were considered for phylogenetic data analysis". This means that any gene containing missing data in any of the samples was excluded completely from the full dataset as we are well aware of the problematic effects of missing data in phylogenetic analyses. Yet "in order to maximize available sequence information <u>within</u> Brassicaceae" (line 530), we made two exceptions for genes *rrn*16 (alignment position 25,102 - 26,517, 1416 bp) and *trn*F-GAA (alignment position 28,456 – 28,519, 64 bp) as these genes were missing only in one outgroup sample respectively, namely in *Tovaria pendula* (rrn16) and *Setchellanthus caeruleus* (*trn*F) from the Capparales (line 531). Therefore, and as also described in line 532, these are the only cases where missing data were replaced with Ns in the matrix. This is why the matrix only contains a total of 1480 Ns, which is indeed less than 0.02%.
But to make these points even clearer, we added some few explanatory remarks in the Material & Methods section.

2) What fossil/calibration information is needed for this work? It's important to point out that there are no Brassicaceae (or even tribal Brassicales) calibration points used in this study – all calibrations are outgroup based calibrations from quite divergent lineages. The manuscript is quite clear on this. This isn't an oversight of the authors, it reflects the reality for Brassicaceae presently – there are only a few, quite controversial, potential calibrations points known to help date lineages within the order or family. So the authors have avoided these. But to set this in context, the approach taken would be akin to only using fern/fern allies/gymnosperms to try and accurately date divergences in the Angiosperms (see figure below)… in my view this is a serious problem for a study that absolutely requires robust calibration points to deal with variation and rate heterogeneity. I am unaware of such an approach having been applied to any other tribe or family and suspect that those who study the pitfalls of dating may seriously question a result from such distant calibration points. As an example of the importance… dating approaches try to deal with rate heterogeneity, but the methods require ample well-distributed calibration points to have any hope of working effectively. Having a study with "outgroup only" calibration points is quite dubious to me. The figure below visually illustrates the issue… this is a quick RAxML tree from the dataset provided by the authors. One can easily see the degree of divergence between in the outgroups (and their calibration points) and the ingroup of interest. Even within the ingroup, there is either insufficient sampling or considerable rate heterogeneity. All of these are issues raise serious questions about robust dating procedures. The figure below was developed because Supplementary figure S1 (which is a rough equivalent to this figure) is illegible in the form provided (you can zoom in but not read anything – the same is true for Figure S2 – not remotely readable in the version provided). I suspect the lack of readability had to do with a conversion issue at the journal rather than the authors original version. When we consider that the manuscript combines potentially inaccurate phylogenetic resolution, ignores known phylogenetic conflict, and applies questionable calibration points… I have reservations about how these beautiful data (the plastid genomes in particular) are being applied. There are so many wonderful directions to go in for these data… this wasn't one of them in my mind.

Dating issue: Our manuscript is the first to provide reliable and "consistent within one study" divergence time estimates for stem and crown group ages of any given tribe. Of course, those may change in future depending on datasets (e.g. nuclear data analyzed within a coalescent framework may be expected to provide slightly different numbers, e.g. simply because of the time of "full coalescent" to be defined and evaluated), but this will have a biological meaning in terms of the mode of separation of lineages. There are <u>no</u> primary calibration points within Brassicaceae, and any fossil evidence (including the "*Thlaspi*" fossil) has turned out to be doubtful and as the reviewer claimed, "*The issues related to precise dating of Brassicaceae phylogeny may never be resolved*". Therefore, we feel very happy with our divergence time

results providing a dataset, which is consistent within a given study and which is largely in agreement with accumulating divergence time estimates from within the family (also showing convergence of values extracted from datasets from different genomes).
Furthermore, since we do not analyze our data in the context of environmental change (and linking dates with, e.g., transitions during geological epochs) none of our conclusions is flawed.
We now also provide a higher resolution pdf of the Supplements to make the figures more readable.

General concerns that are more easily resolved:

How have the authors dealt with poorly supported nodes – they report on line 161 '"a fully resolved maternal phylogeny", but the methods applied will always give a fully resolved tree. That doesn't mean there aren't poorly supported nodes therein. Based on the quick tree above, there are poorly supported nodes therein (but I can't read Supp Figs1-2).

We agree and rephrased to 'well supported'.

Line 169-170. The manuscript notes that their cpDNA findings are congruent with nuclear genome estimates. This isn't accurate and even contradicts what is stated in the introduction of the manuscript. The studies referenced in the manuscript for "congruence" didn't use "nuclear genome" estimates in any modern sense. The two papers that have actually developed a "nuclear genome" estimate are not cited and significant elements are not congruent. The papers cited by these authors are as follows:

25 – this paper is plastid phylogeny…

32 – this paper is a plastid phylogeny with some a single nuclear locus (ITS)

36 – plastid phylogeny

37 – This paper includes RNA-seq data, but that data was not used for phylogenetics. The tree used in this paper was a single plastid gene (ndhF) plastid with one nuclear locus (phyA)

Here, we referred to the time estimates, not the tree topology, and therefore do not include nuclear phylogeny papers without divergence times (e.g. Nikolov et al., 2019). We rephrased to 'This is consistent with *time* estimates based on data from both plastid and nuclear genomes' to make this more obvious to the reader.

Lines 194-196. The authors directly note that they have made, what sound like, formal taxonomic changes to 860 species. I don't think they have really done this. I don't see such a vast formal taxonomic in the paper or supplementary materials? Please clarify.

The species checklist we provide does not comprise formal taxonomic changes to these species, but it has been a critical evaluation of any meaningful synonym used in the past. There are various databases such as PlantList, IPNI, or Tropicos (and others) often suggesting "accepted names" and placing taxa (in this case species) in various and different genera. The number of 860 species which were subject to changes was calculated according to an earlier checklist from 2006 (2006 (Warwick et al., Plant Systematics and Evolution).

However, in order to decide what synonym (this means in what "taxonomic" context) should be used we had to consult not only a simple list of synonyms, but we had to critically evaluate thousands of vouchers, visited many herbaria and inspected the many phylogenetic analyses.

We did not list here all the publications that resulted from the formal taxonomic work, but we added a list of manuscripts at the end of this letter presented by our group in Heidelberg during the course of this project. The list is neither complete nor should it be integrated into this manuscript – it is just to demonstrate the immense taxonomic work.

We think we clearly described what we have done within the supplement, but the reviewer is right that with the main text the given information might be misleading. We have improved this.

Line 212 – there has been much discussion about the potential phylogenetic utility of Brassicaceae characters. Please provide citation of prior work in some way to support the statements.

We moved this to the introduction section, where it is integrated into the paragraph on homoplasy and convergent evolution in morphological characters in Brassicaceae.

Line 235 – what does “realization” of morphological characters really mean? I found this fairly confusing. Can you please define more clearly somewhere?

We rephrased to ‘disparity elaborates on the *presence* of possible character states within a defined taxonomical unit’.

Line 249 – In numerous places terms are used inconsistently in the manuscript. For example, the morphological matrix has characters for “Duration”. But in the main manuscript this is sometimes called “Duration”, other times referred to as “life cycle” (line 249) and sometimes called “life form” (line 327). Please be consistent to avoid confusion with this another terminology.

We changed life cycle to duration.

Line 261-263. This discussion of phylogenetic signal is quite confusing and unclear. If “it” isn’t referring to characters, what is “it” referring to? What is “it” later in the sentence.

We rephrased this sentence.

Line 280 – awkward sentence. Species and genus richness can be represented by numbers, but they aren’t numbers as written in the sentence.

Yes, we changed it to ‘richness’.

Line 313 – please add “Brassicaceae” to the sentence. It sounds like a very general statement, but I think the manuscript is referring to Brassicaceae specifically at the beginning of this paragraph.

Yes.

Line 327-328 – Sentence doesn’t make sense to me

We rephrased to ‘Characters with *a* high *mean* disparity occur in multiple states across the phylogeny (*i.e. multiple states are present in a tribe, such as seen for* life form), generally have a low contribution to lineage differentiation and low pairwise differences between lineages, and are devoid of phylogenetic signal.’

Line 331 – Poorly argued topic. Just because characters have homoplasy doesn’t remotely mean they cannot be used phylogeny reconstruction. If that were true, most all molecular data would be useless as well. The issue being discussed is more likely a combination of limited character space (low information content) combined with high homoplasy. Please clarify and make a clear argument.

We rephrased to ‘high degrees of homoplasy are hindering for phylogenetic reconstruction’.

Line 333 – similar pattern to what? This was unclear.

We specified to ‘similar pattern *of low contribution to lineage differentiation, low pairwise differences and lack of phylogenetic signal*’.

Start at line 342- I find a fair bit of the Discussion unclear on the main points. For example – what is the point of the second paragraph of the Discussion (lines 342-379)? It’s fundamentally a list of examples without context? It would be much better to indicate what the topic is and how these examples illustrate some critical point.

We rephrased fair bits of the discussion.

Line 348. As I recall, this reference (#35) does not support what is stated in this manuscript. I'm fairly sure that paper only demonstrated loss of RCO in A. thaliana, not "repeated loss throughout the family". Please check this citation really supports what is said – my apologies if my memory fails me on this point.

Yes. We adjusted the reference, Vlad et al. is now cited some words earlier, and Kiefer et al. for the repeated loss throughout the family.

There are a number of places in the manuscript where the authors broadly refer to "homoplasy" as being important. Homoplasy is a general term non-specifically covers important mechanisms (e.g., parallelism and convergence). I think that more development of text related to these underlying mechanisms is warranted (rather than use of a blanket less-specific term "homoplasy"). The manuscript is ultimately hitting on important issues of parallelism and convergence… but the text never reaches that level of clarity.

The reviewer is of course correct in their assessment that homoplasy is quite a non-specific term, and rather describes the resulting pattern than the underlying processes. However, as we are not able to distinguish between the mechanisms leading to the observed pattern (and this is miles beyond the scope of our manuscript!), we feel that the, admittedly, more ambiguous term homoplasy is more appropriate.

Line 370 – why start this paragraph with a specific statement about character f27? Why not give the reader a clue as to what the paragraph will be focused on (the topic of the paragraph)? This could help make the Discussion easier to follow.

We agree and re-arranged the sentences.

Line 391-393. Perhaps the sentence is correctly written, but I found it quite confusing. If I were to rewrite it, I believe it literally says "speciation rates are lowest after WGDs because of considerably lower extinction rates after WGDs"? Is this accurate? Seems odd to conclude that "low extinction leads to low speciation"?

Correct. This was supposed to be 'Net diversification rates were lowest in tribes with WGDs due to low extinction rates after WGDs'. We rephrased this sentence.

Line 427 – what is "this" – please be more specific to avoid the reader having to interpret exactly what is meant.

Indeed, and consistently with the other reviewers we completely shortened this paragraph to avoid any interpretations and speculations not substantiated by our data.

Line 438 – I like this paragraph in general, but here the use of "homoplasy" again isn't useful. Homoplasy is a reflection of other more specific topics and it would be far better to talk about those than blanket term "homoplasy" (e.g., parallelism and convergence).

See our reply to the comment about homoplasy above.

Lines 442-447. I didn't find this concluding paragraph to be very convincing. It's a big broad statement and conclusion. I would suggest either remove this or develop the topic into a meaningful paragraph or more worthy of the magnitude implied. Since this seems to be the major "conclusion" – I think much more could be done to tie the ideas into the context of what is already known on the topic. Note that there isn't any discussion on the existing literature that relates to the development of the model presented by the authors.

Same as above - and consistent with the other reviewers: we completely shortened this paragraph to avoid any interpretations and speculations not substantiated by our data.

Line 459 – why is geography relevant to this paper? I don't see it in any data matrix? Section beginning line 482. How much of this data was used in the early 2019 papers (Huang et al 2019 and Nikolov et al 2019)? I don't think this is all new data. Please be precise.

Geography is an important character to determine genera with the interactive key, where it is included as a "character". The morphomatrix we analyze statistically is based on this interactive key, following streamlining of characters (e.g. decreasing the number of characters for better analyzability). We now removed the part of this sentence describing the interactive key, so as not to confuse the reader.

Why didn't the group develop a ribosomal phylogeny from all this beautiful skimming data? I suspect it would show considerable conflict, that is important but ignored. Developing the nrDNA phylogeny is pretty standard with genome skimming data like this.

We constructed a phylogeny from the nuclear encoded rDNA for all samples where we generated new genome skimming data. As could be expected, deeper nodes are not resolved and not much information can be gained from the tree. Nevertheless, we show the results in the Suppl. Fig. S3 and S14, and discuss the results in Suppl. Text S1.

Line 533. Another example of potentially confusing choice of terms. The authors refer to protein coding genes, tRNA, and rRNA as "coding genes". It is generally accepted that "coding genes", as a term, is an abbreviation for "protein coding genes". tRNA and rRNA are not "coding genes" to most readers. Probably best to just say they used "60 genes, including 43 protein coding, 14 tRNA, etc."

Yes. We changed this sentence to 'A total of 60 genes remained, including 43 protein-coding genes, 14 tRNAs and three rRNAs'.

Line 643 – several lines earlier the authors state that the Bivonaeeae wasn't sampled… now it says it was sampled? Please clarify and correct.

Indeed, this was a poorly chosen example for a tribe with only a single species or genus. We now only mention Shehbazieae and Aphragmeae.

Line 646 – "respective numbers" of what? What is this?

Yes, this was not well phrased; changed to 'time estimates'.

Line 663 – lack of resolution from one locus isn't acceptable as evidence for a "hard polytomy"… This is a place to talk about what the recent nuclear genomic phylogenies have found here. Ignoring other data and calling this a "hard polytomy", based on plastid tree, doesn't make sense.

Changed to 'polytomy'.

Line 675 – insert "morphological" before "disparity"

In this context, we mean the specific measure of disparity we calculate here, and therefore did not change it (see also our answer to reviewer 3).

Supplementary Figs S1 and S2 are completely unreadable – even when zooming.

We have included the pdfs on the google drive for supplementary files. We also now provide a higher quality pdf of the supplements.

Taxonomic and systematic contributions provided by D.A. German during the project phase (including formal taxonomy, plus new species descriptions provided by M.A. Koch), in which most of our own new taxonomic data (species checklist) as prerequisite of the morphomatrix was collected – as mentioned above much more work was conducted identifying correct names and synonyms for the roughly 4000 species and deciding what name (synonym) has to be accepted.

This is a calculation for our work from 2013-2017:
New species – 2
New genera – 5
New combinations [incl. few replacement names] (species including few subspecies) – 130
New synonyms (species) – 35
New synonyms (genera) – 1
Typifications (mainly lecto-, few neotypifications; all ranks from a form to a genus) – ~100

2017

German D. A., Koch M. A. 2017. *Eutrema salsugineum* (Cruciferae) new to Mexico: a surprising generic record for the flora of Middle America. – PhytoKeys 76: 13–21. DOI: 10.3897/phytokeys.76.9731

Koch M. A., Karl R., German D. A. 2017. An underexplored biodiversity of Eastern Mediterranean biota: Systematics and evolutionary history of the genus *Aubrieta* (Brassicaceae). – Annals of Botany 119 (1): 39–57. DOI: 10.1093/aob/mcw204

German D. A. 2017. What is *Cochlearia venusta* (Cruciferae)? – Phytotaxa 297 (3): 295–298. DOI: 10.11646/phytotaxa.297.3.12

Dönmez A.A., Ugurlu Aydin Z., Koch M.A. (2017) *Aubrieta al-shehbazii* (Brassicaceae), a new species from Central Turkey. PhytoTaxa 299 (1): 103-110.

Koch M.A., **Grosser, J.** (2017) East Asian *Arabis* species (Brassicaceae) exemplify past hybridization and subsequent emergence of three main evolutionary lineages in East Asia, America and the amphiberingian region. Bot. J. Linn. Society 184 (2): 224–237. https://doi.org/10.1093/botlinnean/box020.

2016

Friesen N., German D. A., Hurka H., Herden T., Oyuntsetseg B., Neuffer B. 2016. Dated phylogenies and historical biogeography of *Dontostemon* and *Clausia* (Brassicaceae) mirror the palaeogeographic history of the Eurasian steppe. – Journal of Biogeography 43 (4): 738–749. DOI: 10.1111/jbi.12658

German D. A., Tekin M., Španiel S., Marhold K., Al-Shehbaz I. A. 2016. A brief taxonomic revision of *Physoptychis* (*Alysseae*, Brassicaceae). – Phytotaxa 258 (1): 75–82. DOI: 10.11646/phytotaxa.258.1.5

Al-Shehbaz I. A., German D. A. 2016. Three new genera in the tribe *Euclidieae* (Brassicaceae). – Novon 25 (1): 12–17. DOI: 10.3417/2016015

German D. A. 2016. Taxonomic notes on miscellaneous Cruciferae. – Turczaninowia 19, 4: 129–135. DOI: 10.14258/turczaninowia.19.4.17

Kadereit J.W., Albach D.C., Ehrendorfer F., Galbany-Casals M., Garcia-Jacas N., Gehrke B., Kadereit G., Kilian N., Klein J.T., Koch M.A., Kropf M., Oberprieler C., Pirie M.D., Ritz C., Röser M., Spalik K., Susanna A., Weigend M., Welk E., Wesche K., Zhang L-B., Dillenberger M.S. (2016) Which changes are needed to render all genera of the German flora monophyletic? Willdenowia 46 (1): 39-91.

2015

German D. A. 2015. Cruciferae (Brassicaceae): Alternative treatment for the "Conspectus of the vascular plants of Mongolia". – Turczaninowia 18, 2: 39–67. DOI: 10.14258/turczaninowia.18.2.4

German D. A., Al-Shehbaz I. A. 2015. Typification of miscellaneous Brassicaceae (Cruciferae) from Central and Middle Asia. – Phytotaxa 221 (1): 57–65. DOI: 10.11646/phytotaxa.221.1.5

German D. A., Al-Shehbaz I. A. 2015. (2369) Proposal to conserve the name *Sisymbrium pumilum* Stephan, non Lam. (*Calymmatium pumilum*, *Olimarabidopsis pumila*, *Thellungiella pumila*) (Cruciferae). – Taxon 64 (4): 849–850. DOI: 10.12705/644

Španiel S., Kempa M., Salmerón-Sánchez E., Fuertes-Aguilar J., Mota J. F., Al-Shehbaz I. A., German D. A., Olšavská K., Šingliarová B., Zozomová-Lihová J., Marhold K. 2015. AlyBase – database of names, chromosome numbers, and ploidy levels of Alysseae, with new generic concept of the tribe // Plant Systematics and Evolution 301 (10): 2463–2491. DOI: 10.1007/s00606-015-1257-3

Yüzbasioglu S., Koch M.A., **Al-Shehbaz I.A.** (2015) Proof of a knowledge database concept: *Aubrieta ekimii* (Brassicaceae), a new species from NW Anatolia (Turkey) - morphological and molecular support. Plant Systematics and Evolution. DOI: 10.1007/s00606-015-1212-3.

2014

Kiefer M., Schmickl R., German D. A., Mandáková T., Lysak M. A., Al-Shehbaz I. A., Franzke A., Mummenhoff K., Stamatakis A., Koch M. A. 2014. BrassiBase: introduction to a novel knowledge database on Brassicaceae evolution. – Plant and Cell Physiology 55 (1): e3. DOI:10.1093/pcp/pct158

German D. A. 2014. Notes on taxonomy of *Erysimum* (*Erysimeae*, Cruciferae) of Russia and adjacent states. I. *Erysimum collinum* and *Erysimum hajastanicum*. – Turczaninowia 17, 1: 10–32. DOI: 10.14258/turczaninowia.17.1.3

German D. A. 2014. New synonyms and combinations in Eurasian Brassicaceae (Cruciferae). – Phytotaxa 173 (1): 31–40. DOI: 10.11646/phytotaxa.173.1.2

Ebel A. L., German D. A., Kupriyanov A. N., Khrustaleva I. A. 2014. Review of mustards (Brassicaceae) of the Kazakh melkosopochnik flora. – Animadversiones Systematicae ex Herbario Kryloviano Universitatis Tomskensis 109: 20–38 (in Russian).

Al-Shehbaz I. A., German D. A., Moazzeni H., Mummenhoff K. 2014. Systematics, tribal placements, and synopses of the *Malcolmia* s. l. segregates (Brassicaceae). – Harvard Papers in Botany 19 (1): 53–71. DOI: 10.3100/hpib.v19iss1.2014.n4

Moazzeni H., Zarre Sh., Pfeil B. E., Bertrand Y. J. K., German D. A., Al-Shehbaz I. A., Mummenhoff K., Oxelman B. 2014. Phylogenetic perspectives on diversification, biogeography and character evolution in the species-rich genus *Erysimum* (*Erysimeae*; Brassicaceae) based on a

densely sampled ITS approach. – Botanical Journal of the Linnean Society 175 (4): 497–522. DOI: 10.1111/boj.12184

Moazzeni H., Zarre Sh., Assadi M., Joharchi M. R., German D. A. 2014. *Erysimum hezarense*, a new species and *Rhammatophyllum gaudanense*, a new record of Brassicaceae from Iran. – Phytotaxa 175 (5): 241–248. DOI: 10.11646/phytotaxa.175.5.1

German D. A. 2014. Revised typifications and nomenclatural notes in N Eurasian Cruciferae. – Willdenowia 44 (3): 351–361. DOI: 10.3372/wi.44.44305

German D. A. 2014. Taxonomic remarks on some Asian *Lepidium* s. l. (Brassicaceae). – Phytotaxa 186 (2): 97–105. DOI: 10.11646/phytotaxa.176.2.4

Al-Shehbaz I. A., German D. A. 2014. A synopsis of the genus *Braya* (Brassicaceae). – Harvard Papers in Botany 19 (2): 161–174. DOI: 10.3100/hpib.v19iss2.2014.n1

German D. A., Friesen N. W. 2014. *Shehbazia* (*Shehbazieae*, Cruciferae), a new monotypic genus and tribe of hybrid origin from Tibet. – Turczaninowia,– Vol. 17, 4: 17–23 (in Russian). DOI: 10.14258/turczaninowia.17.4.3

German D. A. 2014. Some new and revised typifications in N Eurasian Cruciferae. – Turczaninowia 17, 4: 29–41. DOI: 10.14258/turczaninowia.17.4.6

Neuffer B., Hurka H., Friesen N., German D. A., Franzke A. 2014. Evolutionary history of the genus *Capsella* (Brassicaceae) – *Capsella orientalis*, new for Mongolia. – Mongolian Journal of Biological Sciences 12 (1–2): 3–18.

KARL R., KOCH M.A. (2014) Phylogenetic signatures of adaptation: The *Arabis hirsuta* species aggregate (Brassicaceae) revisited. Perspectives in Plant Ecology, Evolution and Systematics (PPEES). DOI: 10.1016/j.ppees.2014.06.001.

2013

German D. A. 2013. (2148) Proposal to reject the name *Hesperis rupestris* (Cruciferae). – Taxon 62 (3): 633–634.

Al-Shehbaz I. A., German D. A. 2013. A synopsis of the genus *Parrya* (Brassicaceae). – Kew Bulletin 68 (3): 457–475. DOI: 10.1007/S12225-013-9450-4

Koch M. A., German D. A. 2013. Taxonomy and systematics are key to biological information: *Arabidopsis*, *Eutrema* (*Thellungiella*), *Noccaea* and *Schrenkiella* (Brassicaceae) as examples. – Frontiers in Plant Science 4: 267. DOI: 10.3389/fpls.2013.00267

German D. A. 2013. (2182) Proposal to conserve the name *Alyssum canescens* (*Ptilotrichum canescens*, *Stevenia canescens*) (Cruciferae) with a conserved type. – Taxon 62 (4): 836–837 DOI: 10.12705/624.18

German D. A., Lazkov G. A., Tojibaev K. Sh., Neveraev U. A. 2013. New data on diversity and geography of the mustards (Cruciferae) in Kyrgyzstan and Uzbekistan. – Botanical Journal (Moscow & St. Petersburg) 98 (9): 1166–1175 (in Russian).

REVIEWERS' COMMENTS:

Reviewer #1 (Remarks to the Author):

Authors satisfactorily implemented my concerns, I do not have any major issues related to the revised ms. version.

I am wondering about the title. Do not you consider "over geological times" to be redundant? I mean, are there other than geological times in the given context? I expect the authors aimed to avoid saying "during (genome) evolution of"...

Reviewer #2 (Remarks to the Author):

This is the revised version of a manuscript that I reviewed a while ago for Nature Communication.
I acknowledge that the authors have considerably clarified their presentation and accordingly here submitted a study of general interest. The manuscript reads well and I congratulate the authors on a nice and stimulating study that is rather typical of this journal.
I have two relatively minor points that could be addressed before publication.
Despite requests, detailed phylogenetic trees presented as Supplementary Information (i.e. Fig. S2) is still impossible to read; which make the paper difficult to fully evaluate. Now that the authors have included new data based on a nuclear locus, this become crucial as the new tree points to discrepancies with presented plastid data and/or prior work (e.g. Camelinae here showing an unexpected split of Arabidopsis vs Capsella). A stronger presentation and further justifications would help the reader being fully convinced of the strengths of the plastid dataset.
I guess that it would also be valuable to (at least) verbally justify how speciation vs extinction rates were estimated/compared in the light of Louca & Pennell. 2020. Nature 580: 502–505. In particular, readers may appreciate know more about the strength of association with WGD across likely scenarios of speciation/extinction. In other words, it would be greatly appreciated that some comments are added about the robustness of conclusions such as (L. 446) “These interactions increase speciation and decrease extinction rates and result in a rather constant and high net diversification, which is consistent with a lack of any downshift of diversification rate”.
Christian Parisod

Reviewer #3 (Remarks to the Author):

In the revised version the AA. have satisfactorily addressed the critical points raised on the original submission.

Reviewer #4 (Remarks to the Author):

see attached pdf

Walden et al. Reviewer 4 – secondary review

I have read the revised manuscript, prior reviewer comments, and author response letter. I have also reread the criteria for publication in Nature Reviews. My initial review and secondary review are grounded in the required criteria for publication in Nature Communications. The authors seem to have taken some of my comments in the primary review personally, I am sorry for that. That was not my intention. Fundamentally I have attempted to provide the review(s), under the guidelines presented by the journal, to the best of my ability. Overall, the rewrite of the paper, following extensive revisions by the authors is a considerable improvement on logical flow, readability, and consistency. They have done a good job here. Do I think this article should be published somewhere? Of course! Does it fit the criteria for publication in Nature Communications, sadly I don't think so. My general reasons for this remain the same and are noted below.

If, in the future, I wish to recommend a paper to my students or colleagues on the topic of resolving critical phylogenetic relationships to investigate the distribution of WGD events and the potential impact of these duplication on morphological variation and diversification, I do not think this paper is the one I would recommend. While the authors provide beautiful data that are individually major advances for Brassicaceae research, the fundamental study system is sufficiently complicated by a combination of issues (some beyond the author's control) that I find it hard to consider this a model example for general research on these topics.

First, a plastid maternal (fundamentally single locus assembly) isn't the modern norm for species-tree estimates needed to address the questions at hand. The Brassicaceae has displayed extensive incongruence between nuclear and plastid derived relationships, an issue that has reared its head among closely related species, within tribes, and among tribes in various examples. This beautiful plastid phylogeny wasn't the appropriate choice for the work in my view. Accurate phylogenetic resolution among species (not among plastids) is required for this work and is questionable because of the phylogeny.

Second, a long somewhat fruitless debate has illustrated the difficulty in generating a time calibrated phylogeny for the family. There are no reliable Brassicaceae fossils. In the response the authors actively say this may be an unsolvable issue and that time calibration is problematic. I agree. So why they then say "we are very happy" with our divergence estimates? How can we be happy with dating when there are no ingroup fossils to date with? Yes, they have done the best with what is available, but the system is quite suboptimal for questions that require accurate divergence estimates.

Lastly, some other responses to reviewer comments don't make a lot of sense to me (further discussion below). So in my view, the paper has many strong elements (mostly the generation of beautiful useful data for Brassicaceae) research, but I don't think the study questions and implemented with that data represent the model that Nature Communications strives to promote in limited publication space.

I do think these views are simply my own and erroneous. The aforementioned concerns are in fact very nicely highlighted in a recent review article by Clark and Donogue ("Whole genome duplication and

plant macroevolution" Trends in Plant Evolution, 2018, 23:933-945). There are four critical bullet points on the Highlights page that precedes the article in the journal. Here are two of those four points:

"The absolute timing of WGD events remains poorly constrained and poorly understood, but many hypotheses regarding the role of WGD in plant evolution depend on precise estimates."

> Precise accurate estimate are unlikely to have been available in the single best plastid only tree primarily relied on by Walden et al.

"WGD as a driver of plant morphological diversity is an appealing hypothesis, but requires a framework which can quantify morphological variation between lineages and through time."

> Walden et al. even admit that the time element for Brassicaceae is a serious problem. Accurate resolution between lineages is also required (as noted by the previous bullet point).

Below are additional explanations of concerns:

Reviewers, 1, 2, and 4 all highlighted concerns with the use of maternal only phylogeny for this work. The author responses to this issue vary by section and reviewer, ultimately becoming a little confusing to me. I think the authors are interpreting these comments as "why didn't you just use Nikolov data" rather than "why didn't the work apply data like that of Nikolov et al." These sorts of data are needed for more accurate species trees estimates, they are amenable to rare samples from herbarium specimens, etc, etc. The author's responses to these issues aren't logical to me. For example, one primary reason stated for not using nuclear data is that the Nikolov phylogeny wasn't time calibrate… Why does that matter? Nobody is arguing that the Nikolov data alone are what were needed – rather an extention of those data or a similar data source (including increased taxon sampling). Furthermore, even if the Nikolov data were sufficient, what sort of response is "the tree wasn't calibrated"? The data are available; just calibrate it if you need that information.

Furthermore, in the author's response they note that a prior tree, the cpDNA tree of Nikolov et al. is smaller but congruent with their tree. This is entirely true, but it seems to miss the obvious and critical point. Nikolov and many others have highlighted considerable incongruence between plastid and nuclear phylogenetic estimates across taxonomic levels in the family. Arguing that congruence in plastid estimates (a single genetic locus) is therefore good and negates the need for a good species tree from many nuclear loci doesn't address the issue.

When it comes to using additional plastid information for the plastid phylogeny, I would want to use all available useful plastid genomes as part of my plan. Seems logical… However, the authors strongly argue that other plastid genomes (>50) (from sources like Nikolov et al.) are not useful because they haven't been circularized, aren't vouchered, etc. This makes no sense whatsoever for the phylogenetic study. Many of those plastid genomes (including all from Nikolov) are from well vouchered material, and the vast majority (at least 50) include all the gene space actually used by Walden et al. By this I mean Walden et al. ***only use the genic portion of the genome to build the phylogeny.*** The full

circularized genome isn't relevant for either study, the rest of the genome was discarded by both sets of authors. That said, these circularized genomes are a wonderful resource for other research downstream, but the factors has no impact on the current results of the analyses conducted and discussed herein.

Perhaps mostly importantly, my prior review extensively discussed "outgroup only" calibration points for this study. The authors made little attempt to really address the issue and convince us that this isn't an important issue. They state, in the rebuttal, they agree that a confidently inferred time calibrated Brassicaceae phylogeny may not be possible but that they are "very happy" with their calibration. I don't understand that argument. I would recommend that the editors ask an expert on calibrating phylogenies who does not work with Brassicaceae if this was good practice.

In response to concerns about selecting the same model for all data partitions, the authors don't provide much to improve confidence. They were also "surprised" by the finding that mRNA, tRNA, and protein coding sequences all share the same best model… but then argue this is standard and "it is the same tool used by three other papers". I believe the three other papers were only looking at protein coding genes, but this manuscript is combining protein coding sequences, tRNA genes, and rRNA. It is unlikely that these three classes of genes primarily only differ in rate heterogeneity as suggested by partition finder. So I still have concerns here, but it probably doesn't represent a fatal error of the work, it's just unlikely a "best practice", just because partition finder says it is so.

Reviewer #1 (Remarks to the Author): Authors satisfactorily implemented my concerns, I do not have any major issues related to the revised ms. version. I am wondering about the title. Do not you consider "over geological times" to be redundant? I mean, are there other than geological times in the given context? I expect the authors aimed to avoid saying "during (genome) evolution of"...

Thanks for the positive statement and the suggestion (how) to shorten the title, which is fully in agreement with editor's comments.

Reviewer #2 (Remarks to the Author):
This is the revised version of a manuscript that I reviewed a while ago for Nature Communication. I acknowledge that the authors have considerably clarified their presentation and accordingly here submitted a study of general interest. The manuscript reads well and I congratulate the authors on a nice and stimulating study that is rather typical of this journal. I have two relatively minor points that could be addressed before publication.
Despite requests, detailed phylogenetic trees presented as Supplementary Information (i.e. Fig. S2) is still impossible to read; which make the paper difficult to fully evaluate. Now that the authors have included new data based on a nuclear locus, this become crucial as the new tree points to discrepancies with presented plastid data and/or prior work (e.g. Camelinae here showing an unexpected split of Arabidopsis vs Capsella). A stronger presentation and further justifications would help the reader being fully convinced of the strengths of the plastid dataset.

The Arabidopsis-Capsella split has been cited herein (Forsythe et al. 2017, no. 38) and it marks exactly one single and first examples for which incongruencies among plastome and nuclear genome derived phylogenies are best explained by massive and biased gene transfer from one lineage (or tribe) to another, and which cannot be resolved with classical tree-building methods, but it needs unravelling the individual evolutionary history. Herein rDNA data – as explained - do not resolve among tribes – but helps substantially to define monophyletic groups on tribal level and mark complex and complicated evolutionary histories. We think that all the respective information is best placed with Supplementary Note 1. Here we also mentioned and explained various examples and details, e.g. the Camelineae case (ref. 57), as mentioned and asked for by the reviewer. There is not sufficient space to place and discuss this in more detail with the main manuscript.

As for tree resolution in the Suppl. Information: This seems to be a problem with the PDF generating tool of the Editorial Manager. The WORD-Document (and original figures) is fine, same is true for our PDF-file when converting using ADOBE Professional. We will take care with the current resubmission.

I guess that it would also be valuable to (at least) verbally justify how speciation vs extinction rates were estimated/compared in the light of Louca & Pennell. 2020. Nature 580: 502–505. In particular, readers may appreciate know more about the strength of association with WGD across likely scenarios of speciation/extinction. In other words, it would be greatly appreciated that some comments are added about the robustness of conclusions such as (L. 446) "These interactions increase speciation and decrease extinction rates and result in a rather constant and high net diversification, which is consistent with a lack of any downshift of diversification rate".

Indeed, the reviewer is fully right that there is a long lasting and still pending discussion on robustness of rate estimates. We used for our analyses "association between tribal level data" published data (Huang et al. 2019; ref 34) and added a short explanation with the Material & Methods section.

We also provided a critical sentence as suggested by the reviewer in the Discussion section to point towards this issue. And we removed the ending sentence with the discussion referring to those rates.

Christian Parisod

Reviewer #3 (Remarks to the Author):

In the revised version the AA. have satisfactorily addressed the critical points raised on the original submission.

Thanks for the positive statement

Reviewer #4 (Remarks to the Author):
see attached pdf

As for reviewer 4 the original and earlier provided concerns have been recapitulated, and we appreciate the detailed discussion and comments. And as the reviewer said, some of them are out of our own control.

1) Inaccurate phylogenetic resolution: I assume the reviewer refers here to the "true species tree" – because the phylogenies are highly resolved. This is an issue that cannot be solved until a family-wide phylogenetic tree based on nuclear genomes is available, compared with other lines of evidence (such as plastome data) and until all information is incorporated into a description of the evolutionary history of individual evolutionary lineages, tribes and clades (which may not be possible to be presented with a simple cladogenetic tree). But actually, this is all speculative, and we highly appreciate the suggestion to include a short paragraph in the Discussion section pointing towards the limits of our study, but also highlighting future challenges and perspectives.

2) Divergence time estimates: Indeed, this is an "endless" debate, and since in the past many flaws have been published, we aimed to set estimates into an accurate context. This is what we have done herein – providing estimates, which are consistent within one single study. This is also why we stated "we are happy". We also mentioned that for this reason we did not present any interpretation of given environments 20, 25 or 30 million years ago, but restrict our analyses to time intervals only (lag phase, etc.). Consequently, we also did not provide any speculation about absolute timing of WGD events, although previous papers provided numbers here! So, we fully agree with reviewer 4.

There are few other comments, which may need a short reply:

Usage of other plastome data: The plastome data, for example, of Nikolov et al. (2019) have not been used, because they were not available at the time of our data analyses. Additionally, those data are not needed, because they are covered with our sampling space. Furthermore, we aimed at providing data of the highest quality, and, therefore, we decided at the very beginning of the project to present high quality, fully annotated circular plastome data. This will allow any research in future to work with the data in a very convenient and easy way. The same arguments are true for few other data sets. Detailed comments and explanations have been provided with our earlier letters.

The prediction of partionfinder for model choice has been documented and explained. Guo et al. (2017) concluded for example for a smaller but also family-wide plastome data sets: "Regardless of the data partition strategy in our ML analysis, the majority of relationships across the family were consistent and well supported." And also in their analyses "unpartitioning" had the highest likelihood scores (Table S5). [Guo X. et al. (2017) Plastome phylogeny and early diversification of Brassicaceae. BMC Genomics 18: e176.].
